# Comparative Study on the Effect of Phenolics and Their Antioxidant Potential of Freeze-Dried Australian Beach-Cast Seaweed Species upon Different Extraction Methodologies

**DOI:** 10.3390/ph16050773

**Published:** 2023-05-22

**Authors:** Vigasini Subbiah, Faezeh Ebrahimi, Osman T. Agar, Frank R. Dunshea, Colin J. Barrow, Hafiz A. R. Suleria

**Affiliations:** 1 Centre for Sustainable Bioproducts, Deakin University, Waurn Ponds, VIC 3217, Australia; vsubbiah@deakin.edu.au (V.S.); colin.barrow@deakin.edu.au (C.J.B.); 2 School of Agriculture, Food and Ecosystem Sciences, Faculty of Science, The University of Melbourne, Parkville, VIC 3010, Australia; ebrahimif@student.unimelb.edu.au (F.E.); osman.agar@unimelb.edu.au (O.T.A.); fdunshea@unimelb.edu.au (F.R.D.); 3 Faculty of Biological Sciences, The University of Leeds, Leeds LS2 9JKT, UK

**Keywords:** seaweeds, freeze-drying, conventional extraction, ultrasonication, phenolic compounds, antioxidant activity, LC-ESI-QTOF-MS/MS, HPLC-PDA

## Abstract

Brown seaweed is rich in phenolic compounds and has established health benefits. However, the phenolics present in Australian beach-cast seaweed are still unclear. This study investigated the effect of ultrasonication and conventional methodologies using four different solvents on free and bound phenolics of freeze-dried brown seaweed species obtained from the southeast Australian shoreline. The phenolic content and their antioxidant potential were determined using in vitro assays followed by identification and characterization by LC-ESI-QTOF-MS/MS and quantified by HPLC-PDA. The *Cystophora* sp. displayed high total phenolic content (TPC) and phlorotannin content (FDA) when extracted using 70% ethanol (ultrasonication method). *Cystophora* sp., also exhibited strong antioxidant potential in various assays, such as DPPH, ABTS, and FRAP in 70% acetone through ultrasonication. TAC is highly correlated to FRAP, ABTS, and RPA (*p* < 0.05) in both extraction methodologies. LC-ESI-QTOF-MS/MS analysis identified 94 and 104 compounds in ultrasound and conventional methodologies, respectively. HPLC-PDA quantification showed phenolic acids to be higher for samples extracted using the ultrasonication methodology. Our findings could facilitate the development of nutraceuticals, pharmaceuticals, and functional foods from beach-cast seaweed.

## 1. Introduction

Marine biological resources have come under increased attention in the past decade due to their natural bioactive compounds having potential as leads for the development of new functional foods or drugs. Seaweeds, which are part of aquatic ecosystems, have functional properties that make them useful compounds for various industries, including food, cosmeceuticals, bio-stimulant, animal feed, and fertilizer [1,2]. Seaweeds are rich in non-nutritional compounds that are beneficial to human health, such as phenolics compounds [3]. These compounds are secondary metabolites that are produced as part of the seaweed’s defence mechanism, via the shikimate/phenylpropanoid pathway [4]. Due to the presence of phenolic compounds, seaweeds can have some ability to improve the immune system, protect against radiation, and contribute to the treatment of chronic diseases, including cardiovascular disease, obesity, cancer, and diabetes, as well as neuroprotective diseases including epilepsy, Parkinson’s, autism, and Alzheimer’s [5]. 

In Australia, large amounts of seaweed biomass accumulate on the shores due to storms, winds, and currents, which results in seaweed becoming detached and washed to the shores. The seaweed on the shore can cause strong odour and release greenhouse gases. However, this seaweed could potentially be utilised as a biomass for the production of functional ingredients to develop the latest biotechnological applications [6]. Seaweeds collected from the shore are dried before being used in industrial processing or nutritional evaluation [7], since dried seaweeds can be stored for years without extensive loss of their functional compounds [8]. Researchers have found that freeze-drying is an excellent method for producing high-quality dried products. Cruces et al. [9] reported that freeze drying has the ability to retain high antioxidant potential. 

Phenolic compounds exist in free and bound forms. Free phenolics can be extracted easily with solvents, while bound phenolics require various extraction methods, including chemical, biological, and physical methods, as they are entrapped within plant cells [2,10]. The conventional methods used in the extraction are percolation, maceration, heat reflux, and Soxhlet apparatus extractions for extracting phenolic compounds. However, conventional methods can consume large amounts of solvents, produce low yields, and require operating at high temperature that can damage bioactive compounds during extraction [11]. An alternative to conventional methods is ultrasound-assisted extraction, which involves the breakdown of bubbles and disrupts the plant cell walls which thus increases the mass transfer of intracellular components into the solvent [12]. Several studies including Rodrigues et al. [13], Kadam et al. [14] and Hassan, Pham, and Nguyen [15] have used ultrasound extraction in their studies. Ummat et al. [16] conducted a comparative study between ultrasound and conventional extraction of phenolic compounds, phlorotannins, and their antioxidant activities. The ultrasound extraction of the sample produced a higher yield of total phenolics, phlorotannins, flavonoids, and exhibited stronger antioxidant potential. 

Various organic solvents can be used for the extraction of phenolic compounds, but the recovery of these compounds depends on the solubility of the phenolics in the solvent. The polarity of the solvent also plays a critical role in enhancing the solubility of phenolics, making it challenging to develop a standardised methodology to extract all phenolics from seaweeds [17]. The evaluation of phenolic compounds can be completed using different spectrophotometric-based in vitro assays [18], including total phenolic content (TPC), total flavonoid content (TFC), total tannin content (TCT), 2,4-dimethoxybenzaldehyde (DMBA), Prussian blue assay (PBA), and Folin–Denis assay (FDA). The antioxidant activity of the phenolic compounds can be assessed using various in vitro methods, such as 2,2′-diphenyl-1-picrylhydrazyl (DPPH) antioxidant assay, 2,2’azino-bis-3-ethylbenzothiazoline-6-sulfonic acid (ABTS), reducing power assay (RPA), hydroxyl radical scavenging activity (·OH-RSA), ferrous ion chelating activity (FICA), and ferric reducing-antioxidant power (FRAP) assay [19]. Phenolic compounds can be characterised and identified using liquid chromatography coupled with electrospray-ionisation quadrupole time-of-flight mass spectrometry (LC-ESI-QTOF-MS/MS). The compounds are quantified using high performance liquid chromatography (HPLC) combined with photodiode array detector (PDA). According to Zhong et al. [20], the major phenolic compounds identified from eight different seaweed species were gallic acid, cinnamoyl glucose, chlorogenic acid, caffeic acid, and coumaric acid. 

In this study, we estimated the total phenolics including free and bound phenolics and their antioxidant potential in freeze-dried samples using two different extraction methodologies that were conventional and ultrasonication extraction, with four different solvents. The phenolics were identified and characterised via LC-ESI-QTOF-MS/MS and the phenolic compounds were quantified using HPLC-PDA. This study has provided information on the phenolic content and antioxidant properties of the freeze-dried seaweeds and thus promotes and provides evidence toward further investigation of their application in food and pharmaceutical industries.

## 2. Results and Discussion

### 2.1. Total Phenolics, Flavonoids Tannin and Phlorotannin Content

Bioactive compounds are natural metabolites that are very common in the plant kingdom and provide numerous benefits. Phenolic compounds in particular have attracted great interest in industries ranging from food production to pharmaceuticals due to their protective roles in human health [21]. In this study, total phenolics, flavonoids, tannin and phlorotannin content are shown in Table 1 which combines data for free and bound (Tables S1 and S2) phenolics in the seaweed samples, including *Cystophora* sp., *Phyllospora comosa*, *Sargassum* sp., *Ecklonia radiata*, *Durvillaea* sp. There were estimated by ultrasonication assisted extraction (UAE) and conventional extraction using different solvents (70% acetone, 70% methanol, 70% ethanol, and absolute ethyl acetate). 

The total phenolic content (TPC) among the seaweed species revealed a significant difference (*p* < 0.05), according to the Tukey statistical analysis (Table 1). Further data analysis indicates ultrasonication to be a more effective methodology for extracting total phenolic content (TPC) than the conventional method. Among the species, *Cystophora* sp. showed significant TPC values (*p* < 0.05) when extracted with 70% acetone (19.35 mg GAE/g, ultrasonication; 18.27 mg GAE/g, conventional), 70% ethanol (28.92 mg GAE/g, ultrasonication; 15.49 mg GAE/g, conventional), and 70% methanol (22.74 mg GAE/g, ultrasonication; 18.59 mg GAE/g, conventional), while negligible amounts were found in absolute ethyl acetate (1.66 mg GAE/g, ultrasonication; 0.96 mg GAE/g, conventional). In ultrasonication extracts, phenolic content ranged from 0.34 mg GAE/g (*Sargassum* sp., absolute ethyl acetate) to 28.92 mg GAE/g (*Cystophora* sp., 70% ethanol), whereas in conventional extraction, phenolic content ranged from 0.09 mg GAE/g (*Durvillaea* sp., absolute ethyl acetate) to 18.59 mg GAE/g (*Cystophora* sp., 70% methanol). The higher phenolic content from ultrasonication might be due to the ability to break the cell walls and facilitate extraction of phenolic compounds [22]. Previously, *Carpophyllum plumosum* (sub-tropical region, New Zealand) was identified to have significantly higher values (*p* < 0.05) than *Phyllospora comosa* (temperate region, Tasmania) using conventional methodology, which is consistent with our study [23]. In another study, the Australian seaweeds collected from Bateau Bay demonstrated the total phenolic content of *Sargassum vestitum* (141.91 mg GAE/g), *Sargassum linearifolium* (47.06 mg GAE/g), *Phyllospora comosa* (67.78 mg GAE/g), and *Sargassum podacanthum* (43.13 mg GAE/g) extracted using the ultrasonication method with 70% ethanol [24]. The variation in phenolic content could be due to differences in seaweed species and sampling locations [25]. According to our study, ultrasonication extraction generally produces higher TPC values compared to conventional extraction. The 70% ethanol is the most effective extracting solvent for most seaweed samples, especially when used with ultrasonication extraction. The 70% methanol and 70% acetone produce moderate TPC values, regardless of the extraction methodologies used. Absolute ethyl acetate produced the lowest TPC values for all seaweed samples, regardless of the extraction methods used.

Flavonoids are a widespread and diverse group of natural compounds. These compounds possess biological activities including radical scavenging activity [26]. The total flavonoids content of the seaweed samples was determined using the aluminium chloride method. A significant difference (*p* < 0.05) in the total flavonoid content was observed among the extraction, species, and solvents in our study. The total flavonoid content ranged between 0.67 mg QE/g (*Sargassum* sp., 70% acetone) to 18.23 mg QE/g (*Durvillaea* sp., absolute ethyl acetate) in ultrasonication whereas the conventional extraction ranged from 0.04 mg QE/g (*Durvillaea* sp., 70% methanol) to 6.52 mg QE/g (*Phyllospora comosa*, absolute ethyl acetate). The total flavonoid content was high in absolute ethyl acetate extracted by the conventional methodology of *Phyllospora comosa* (6.52 mg QE/g), *Durvillaea* sp. (6.35 mg QE/g) and *Sargassum* sp. (6.35 mg QE/g) whereas the ultrasound extraction of absolute ethyl acetate (*Durvillaea* sp., 18.23 mg QE/g) and 70% methanol solvent (*Ecklonia radiata*, 11.15 mg QE/g) exhibited higher flavonoid content. The findings of this investigation reveal that ultrasound extraction resulted in higher flavonoid concentration, possibly partly due to degradation of the flavonoid compound during long processing times during conventional methodology [27]. Our study estimated the flavonoid content extracted by the solvent 70% ethanol of *Sargassum* sp., to be 2.79 mg QE/g (ultrasonication) and 0.96 mg QE/g (conventional) whereas another seaweed reported the ethanol extracted from *Sargassum* sp., to be 42.6 mg QE/g [28]. The difference in the flavonoid content is likely due to the different collection regions of the *Sargassum* sp. 

In our study, the total tannin content ranged from 0.27 mg CE/g (*Sargassum* sp., 70% acetone) to 2.86 mg CE/g (*Phyllospora comosa*, 70% methanol) in ultrasonication method whereas in conventional method the total tannin content ranged from 0.02 mg CE/g (*Phyllospora comosa*, 70% acetone) to 27.54 mg CE/g (*Ecklonia radiata*, absolute ethyl acetate). Tannin content was not observed in solvents other than ethyl acetate during the conventional extraction of *Ecklonia radiata*. Tannin content was observed only in 70% methanol and 70% ethanol solvents in the ultrasonication method. However, surprisingly, the highest tannin content among all samples and methods was obtained from conventional extraction with absolute ethyl acetate of *Ecklonia radiata*. The tannin content from the brown seaweed *Ecklonia radiata* (27.54 mg CE/g, absolute ethyl acetate) was significantly higher than (*p* < 0.05) *Cystophora* sp. (2.55 mg CE/g, 70% methanol), *Durvillaea* sp. (1.61 mg CE/g, 70% methanol), *Sargassum* sp. (0.43 mg CE/g, 70% methanol) and *Phyllospora cosmosa* (0.34 mg CE/g, 70% methanol) when extracted by conventional method, whereas the brown seaweed, *Phyllospora comosa* (2.86 mg, 70 % methanol) was significantly higher than (*p* < 0.05) *Durvillaea* sp. (2.12 mg CE/g, 70% methanol), *Ecklonia radiata* (1.29 mg CE/g, 70% acetone), *Cystophora* sp. (0.94 mg CE/g, 70% ethanol), *Sargassum* sp. (0.27 mg CE/g, 70% acetone) in ultrasonication methodology. The difference in tannin content among different solvents is due to the solubility of tannin in the solvent, as well as the molecular weight and degree of polymerization [29]. In a previous study, conventionally extracted *Sargassum* sp. and *Ecklonia* sp. using 80% ethanol were estimated to have 5.62 µg CE/g and 166.87 µg CE/g, respectively [20]. The difference in tannin content between the two seaweeds may be due to variations in light intensity exposure, size, ultraviolet radiation, species, age, and salinity [25].

The phlorotannin content were estimated using three assays, DMBA, FDA, and PBA. In the DMBA assay, the absolute ethyl acetate extracted ultrasonication methodology of *Ecklonia radiata* (8.29 PGE mg/g) and *Durvillaea* sp. (8.22 PGE mg/g) were significantly higher compared to other solvents and species. The highest concentration of phlorotannin was extracted using conventional methodology from absolute ethyl acetate *Ecklonia radiata* (6.57 PGE mg/g), followed by *Sargassum* sp. (6.32 PGE mg/g) and *Phyllospora comosa* (6.01 PGE mg/g). In PBA, ultrasonication extraction of *Phyllospora comosa* (12.62 PGE mg/g, 70% methanol) yielded higher phlorotannin content than *Cytosphora* sp. (8.61 PGE mg/g, 70% methanol) and *Cytosphora* sp. (8.11 PGE mg/g, 80% ethanol). In comparison, conventional extraction resulted in the highest phlorotannin content for *Cytosphora* sp. (7.35 PGE mg/g, 70% methanol), followed by *Sargassum* sp. (7.03 PGE mg/g, 70% methanol), *Phyllospora comosa* (6.95 PGE mg/g, 70% ethanol), *Cytosphora* sp. (6.2 PGE mg/g, 70% ethanol), and *Durvillaea* sp. (6.19 PGE mg/g, 70% methanol). In the FDA, 70% ethanol extracted *Cystophora* sp. (14.2 PGE mg/g) exhibited the highest presence of phlorotannin in ultrasonication, followed by *Phyllospora comosa* (8.57 PGE mg/g, 70% acetone) and *Sargassum* sp. (7.64 PGE mg/g, 70% acetone). However, conventional extraction of *Durvillaea* sp. with 70% acetone resulted in a higher phlorotannin content (20.04 PGE mg/g) than ultrasonication. Overall, ultrasonication extracted more phlorotannin compounds, and the solvents used to extract phlorotannin content varied among the phlorotannin assays. The reason for this variation might be due to the slightly different mechanisms of the assays, which interact with the target compounds differently and have varying sensitivities [30]. 

The extraction of bioactive compounds from seaweed is an important step in the development of functional foods and nutraceuticals with potential health benefits. The efficiency of extraction methods can vary depending on the sample and the solvent used. The results of this study suggest that ultrasonication is generally a more efficient method of extraction for obtaining higher TPC, TFC, TCT, DMBA, PBA, and FDA values compared to conventional extraction methods. The choice of extraction solvent can also have a significant impact on the chemical composition of each sample.

### 2.2. Antioxidant Potential

Seaweeds have been widely studied for their antioxidative properties due to the presence of phenolic compounds [31]. The study utilised a diverse range of methods, including the DPPH, ABTS, FRAP, FICA, OH-RSA, RPA, and TAC assays, to measure the antioxidant activity in various extracts obtained from five different species of seaweed. In this study, antioxidant potential are shown in Table 2, which is combined data for free and bound (Tables S3 and S4) phenolics in the seaweed samples. The data was analysed using the Tukey statistical analysis method to compare the antioxidant activity of the different extracts. Seaweed is a complex system, and therefore, using several antioxidant assays is necessary to account for the different interactions present. There is no single method that can accurately measure the antioxidant properties of seaweed. Thus, using a combination of assays provides a more comprehensive understanding of the antioxidant capabilities of seaweed [32]. 

One of the most commonly used methods to evaluate antioxidant activity is the DPPH method, which is widely recognised for its effectiveness in assessing the antioxidant potential of various samples. Table 2 shows the DPPH scavenging capacities of five different seaweed species using various solvents and extraction methods. The study found significant variations in DPPH scavenging activity among the species, methods, and solvents used. The conventional extraction method showed similar antioxidant potential as ultrasonication. The highest scavenging activity was found in the 70% acetone extract of *Cystophora* sp. followed by *Sargassum* sp., *Phyllospora comosa*, *Ecklonia radiata*, and *Durvillaea* sp. when using ultrasonication. On the other hand, the highest scavenging activity was found in the 70% acetone extract of *Cystophora* sp., followed by *Ecklonia radiata*, *Phyllospora comosa*, *Sargassum* sp., and *Durvillaea* sp., when using conventional extraction. Although acetone was found to be the most effective solvent for extraction antioxidants in our study, previous research has demonstrated high antioxidant activity in *Ecklonia* sp. using ethanol solvent and ultrasonication extraction [33]. It is important to note that there are no ideal organic solvents that would extract total antioxidants, as phenolics can vary in polarity and also be bound with carbohydrate or protein [34]. Additionally, a previous study has demonstrated the presence of DPPH stable free radical scavenging activity in *Sargassum* sp., which supports the findings of our study [35].

FRAP is an important antioxidant assay used to measure the reducing power of a sample. Table 2 summarises the antioxidant capacities of different seaweeds, which were extracted via conventional and ultrasound methods. Among the species analysed, the 70% acetone extract of *Cystophora* sp. had the highest FRAP value, followed by 70% acetone extract of *Sargassum* sp. and 70% methanol extract of *Sargassum* sp., both obtained from ultrasound extraction. On the other hand, *Cystophora* sp. (33.48 mg TE/g, 70% methanol; 33.45 mg TE/g, 70% ethanol; 32.54 mg TE/g, 70% acetone) had higher antioxidant potential than other species using conventional methodology. Overall, *Cystophora* sp., *Sargassum* sp., and *Ecklonia radiata* had higher antioxidant potential in solvents of 70% methanol, 70% ethanol, and 70% acetone. However, negligible antioxidant potential was detected in the solvent of absolute ethyl acetate. Our study demonstrated lower antioxidant potential in 70% ethanol extracted *Durvillaea* sp. (11.39 mg TE/g, ultrasonication; 5.83 mg TE/g; conventional) when compared with another study that showed the antioxidant potential of *Durvillaea antarctica* extracted with absolute ethanol to be 20.6 mg TE/100 g and 50% ethanol to be 80.3 mg TE/100 g [36]. The reason might be due to difference in species, the location of collection of the seaweed, and environmental conditions [25].

ABTS is a commonly used method for measuring the antioxidant activity of a substance. It measures the ability of an antioxidant to scavenge ABTS radical cations and convert them into a colorless form [37]. In the current study, significant differences (*p* < 0.05) were observed among seaweed species in the ABTS assay. The antioxidant potential ranged from 4.86 mg TE/g (*Sargassum* sp., ethyl acetate) to 64.15 mg TE/g (*Cystophora* sp., 70% acetone) in the ultrasonication method. In contrast, the antioxidant potential ranged from 1.87 mg TE/g (*Durvillaea* sp., ethyl acetate) to 42.5 mg TE/g (*Cystophora* sp., 70% acetone) using the conventional methodology. The antioxidant potential of *Cystophora* sp. was high in both extraction methodologies and in solvents of 70% acetone, 70% methanol, and 70% ethanol. However, a previous study showed that *Durvillaea antarctica* extracts were 0.71 mg TE/100 g (absolute ethanol) and 6.34 mg TE/100 g (50% ethanol) [36] while our study showed higher antioxidant potential in 70% ethanol extract (18.36 mg TE/100 g, ultrasonication; 12.29 mg TE/100 g, conventional). The difference in results might be due to difference in varieties, growing region, extraction solvent, and solute to solvent ratio.

In ·OH-RSA, the ability of seaweed samples to scavenge hydroxyl radicals were measured [38]. Our study found that the ultrasonication method was more effective than the conventional methodology in extracting antioxidants. Among the seaweeds analysed, *Ecklonia radiata* (134.49 mg TE/g, 70% acetone) had the highest antioxidant potential using ultrasonication, followed by *Phyllospora comosa* (65.42 mg TE/g, 70% methanol) and *Cystophora* sp. (62.67 mg TE/g, 70% methanol). However, the 70% methanol extract of *Phyllospora comosa* (26.63 mg TE/g) and the 70% acetone extract of *Sargassum* sp. (23.37 mg TE/g) had higher antioxidant potential than *Durvillaea* sp. (16.80 mg TE/g, 70% methanol), *Ecklonia radiata* (6.75 mg TE/g, 70% methanol), and *Durvillaea* sp. (6.50 mg TE/g, 70% ethanol), when using the conventional methodology. It is worth noting that no antioxidant activity was detected when using absolute ethyl acetate solvent in conventional extraction. Overall, the ultrasonication method exhibited higher antioxidant activity compared to other methods. Among the solvents tested, 70% methanol and 70% acetone were found to have the highest antioxidant potential. 

In the FICA assay, the chelating ability of seaweed samples was measured by the conversion of ferrozine to ferrous ions [39]. In FICA, among the extractions carried out using ultrasonication, the 70% acetone extract of *Phyllospora comosa* showed the highest antioxidant potential (4.14 mg EDTA/g). In the case of conventional extraction, the best antioxidant potential was found in the absolute ethyl acetate extract of *Ecklonia radiata* (4.46 mg EDTA/g, ethyl acetate). According to the results of Table 2, ultrasonication generally showed higher values of antioxidant potential than the conventional method. 

In RPA, the ultrasound extraction method revealed that the order of antioxidant potential were *Cystophora* sp. (29.27 mg TE/g, 70% ethanol), followed by *Sargassum* sp. (28.47 mg TE/g, 70% acetone), *Phyllospora comosa* (25.76 mg TE/g, 70% acetone), *Phyllospora comosa* (20.46 mg TE/g, 70% methanol), *Ecklonia radiata* (19.1 mg TE/g, 70% methanol) and *Sargassum* sp. (18.52 mg TE/g, 70% ethanol). On the other hand, the conventional method yielded lower antioxidant activity, with *Cystophora* sp. (22.01 mg TE/g, 70% ethanol) having the highest potency, followed by *Phyllospora comosa* (11.90 mg TE/g, 70% ethanol), *Ecklonia radiata* (11.43 mg TE/g, 70% ethanol), *Sargassum* sp. (10.55 mg TE/g, 70% acetone), *Ecklonia radiata* (10.17 mg TE/g, 70% methanol), and *Durvillaea* sp. (9.84 mg TE/g, 70% acetone). Therefore, it can be concluded that the ultrasound method is more effective for extracting antioxidants, with 70% acetone, 70% methanol, and 70% ethanol being the recommended solvents for extraction. 

Total Antioxidant Capacity (TAC) is a measure of the ability of a substance to neutralise free radicals and protect against oxidative stress. TAC is often used as an indicator of the antioxidant potential of foods and natural products, such as seaweed. In TAC, the different species of seaweed are considered to have high bioactive compounds such as total phenolics and flavonoid content, which contribute to their total antioxidant activity. The seaweed with the highest TAC was *Cystophora* sp., extracted using ultrasonication method with 70% acetone extraction, followed by *Sargassum* sp. (70% acetone), *Phyllospora comosa* (70% acetone), *Sargassum* sp. (absolute ethyl acetate), and *Ecklonia radiata* (70% acetone). Comparably high TAC was found in *Cystophora* sp. extracted by conventional method of 70% acetone followed by *Cystophora* sp. (70% ethanol), *Sargassum* sp. (70% acetone), *Ecklonia radiata* (70% ethanol), *Ecklonia radiata* (70% methanol), and *Ecklonia radiata* (70% acetone). The solvents that extracted the highest antioxidant potential within the species were 70% acetone, 70% ethanol, and 70% methanol. 

### 2.3. Correlation

Pearson’s correlation analysis was performed to observe the correlations between phenolic compounds and antioxidant potential for both ultrasonication and conventional methodologies (Table 3). In ultrasonication extraction, TPC showed a significant correlation (*p* < 0.05) with FDA, PBA, DPPH, FRAP, ABTS, TAC, and RPA. The correlation between TFC and DMBA is significant (*p* < 0.05) in both conventional and ultrasonication methodologies. The ultrasonication methodology has shown moderate to strong positive correlations between DPPH and FRAP (*r* = 0.657), ABTS (*r* = 0.690), OH-RSA (*r* = 0.474), TAC (*r* = 0.646), and RPA (*r* = 0.480). However, FICA only displayed a weak positive correlation (*r* = 0.406) with DPPH. Stronger positive correlations were found between DPPH and FRAP (*r* = 0.824), ABTS (*r* = 0.809), TAC (*r* = 0.809), and RPA (*r* = 0.688) using the conventional extraction method. It is worth noting that FICA displayed a negative correlation (*r* = −0.376) with DPPH in conventional extraction. Similarly, through conventional extraction, TPC exhibited a high correlation with DPPH, FRAP, ABTS, PBA, and FDA. TFC showed a significant correlation with DMBA (*p* < 0.05), which was similar to ultrasonication. In both conventional and ultrasonication methodologies, FRAP is significantly correlated with ABTS, RPA, and TAC. Previous studies have shown significant correlation between TPC and DPPH in *Acanthophora spicifera* (Rhodophyta) [40]. In another seaweed study, gallic acid has been found to have a positive correlation with DPPH scavenging activity [41]. Matanjun et al. [42] reported that ABTS had strong correlation with FRAP, which supports our study. 

**Figure 1:**
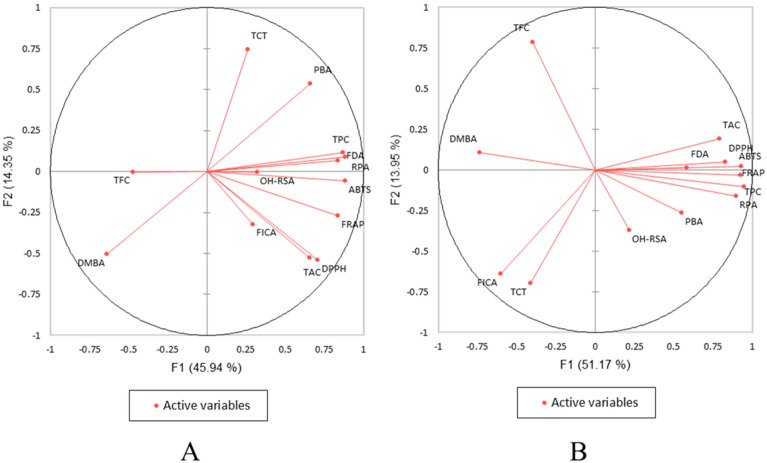
Principal component analysis (PCA) of the phenolic content (TPC, TFC, TCT, DMBA, PBA, FDA) and antioxidant activities (DPPH, ABTS, FRAP, RPA, OH-RSA, TAC) of five brown seaweeds extracted via ultrasonication (**A**) and conventional (**B**) methodologies.

In addition, principal components analysis (PCA, Figure 1) was performed to investigate the overall relationship between the phenolic content and their antioxidant potential extracted via the methodology of conventional and ultrasonication. In ultrasonication, TPC is highly correlated with ABTS, DPPH, TAC, FRAP, and RPA which is same as Pearson’s correlation coefficients (*r*). In conventional methodology, the TPC is highly correlated with PBA, FDA, DPPH, FRAP, ABTS, OH-RSA, and RPA, similar to Pearson’s correlation coefficients (*r*). 

### 2.4. Distribution of Phenolic Compounds in Seaweeds

**Figure 2:**
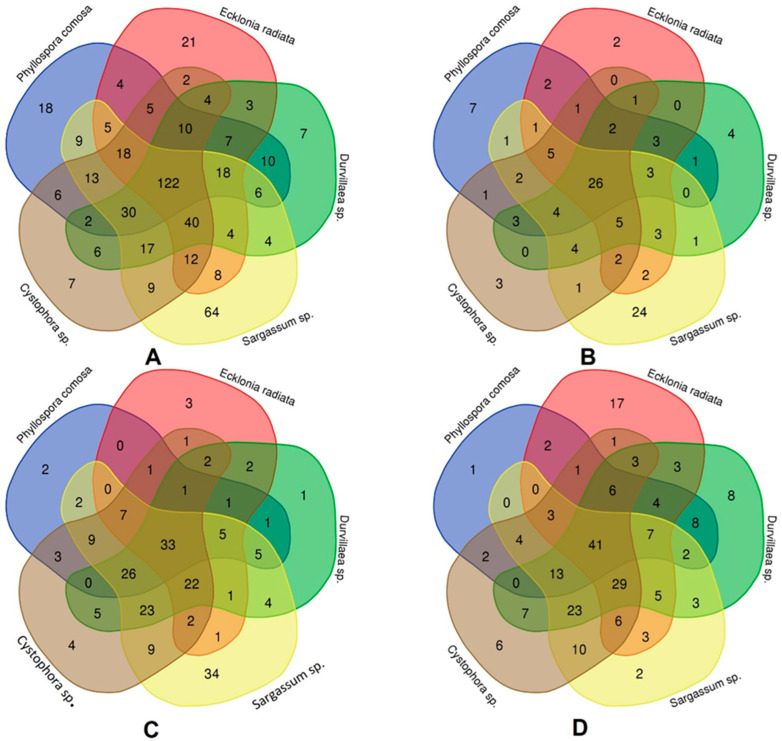
Venn diagram of phenolic compounds presented in different seaweed species. (**A**) shows the relation of total polyphenols among the species (**B**) shows the relation of phenolic acids among the species. (**C**) shows the relation of flavonoids among the species. (**D**) shows the relation between the other polyphenols in different species.

Seaweed contains an extensive range of phenolic compounds in different conjugated forms. A Venn diagram can make it easier to see the distribution of the phenolic compounds. The Venn diagrams (Figure 2) were developed according to the number of phenolic compounds that were detected among the five species of seaweed including *Phyllospora comosa* (blue), *Ecklonia radiata* (red), *Durvillaea* sp. (green), *Sargassum* sp. (yellow), and *Cystophora* sp. (brown). In the Venn diagram (Figure 2A), the unique compounds present in *Cystophora* sp., *Durvillaea* sp., *Phyllospora comosa*, *Ecklonia radiata*, *Sargassum* sp. were 7, 7, 18, 21, and 64, respectively. The maximum overlapping phenolics were 122 among the 5 species of seaweed. The lowest number of phenolics that overlapped were *Cystophora* sp., and *Ecklonia radiata*. A study reported that the seaweed species collected from the shores of Australia and New Zealand, including *Phyllospora* sp., *Ecklonia radiata*, *Durvillaea* sp., *Sargassum* sp., *Cystophora* sp., had high total phenolic content [43]. To support our study, research identified a wide range of phenolic compounds in *Cystophora* sp. and *Sargassum* sp. [44]. In Figure 2B, 26 phenolic acids were common among the 5 seaweed species. The unique compounds in *Ecklonia radiata*, *Cystophora* sp., *Durvillaea* sp., *Phyllospora comosa* and *Sargassum* sp., were 2, 3, 4, 7, and 24, respectively. In Venn diagram (Figure 2C), 33 compounds were common flavonoids among the 5 species of seaweed. The unique flavonoids were 1, 2, 3, 4, and 34 in *Durvillaea* sp., *Phyllospora comosa*, *Ecklonia radiata*, *Cystophora* sp., and *Sargassum* sp., respectively. The highest flavonoids of 26 compounds overlapped with *Phyllospora comosa*, *Durvillaea* sp., *Sargassum* sp., and *Cystophora* sp. The unique other phenolics (Figure 2D) in *Phyllospora comosa*, *Sargassum* sp., *Cystophora* sp., *Durvillaea* sp., and *Ecklonia radiata* were 1, 2, 6, 8, and 17, respectively. The 41 compounds of all 5 seaweed species were overlapped. The highest overlapped compounds were 29, in *Durvillaea* sp., *Ecklonia radiata*, *Sargassum* sp., and *Cystophora* sp.

**Figure 3:**
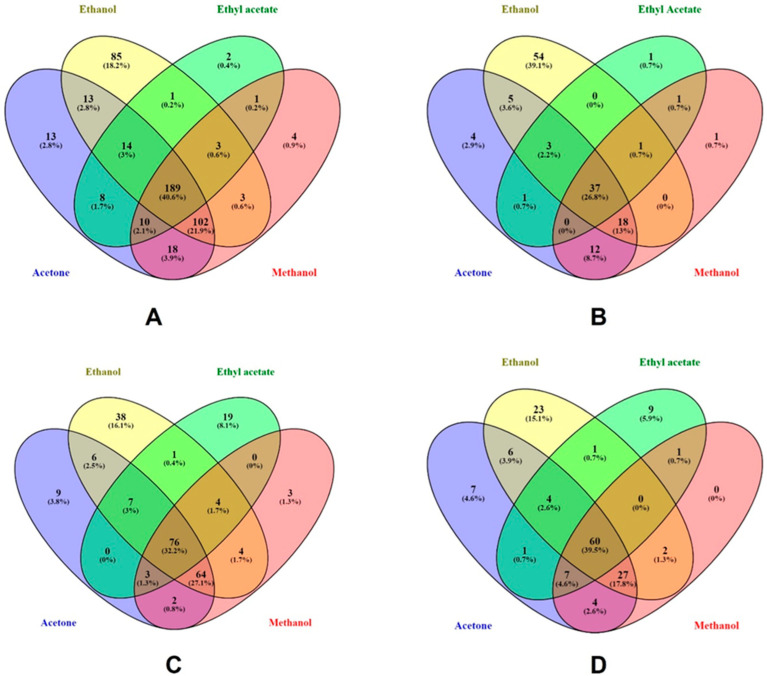
Venn diagram of phenolic compounds presented in different extracted solvents. (**A**) shows the relation of total phenolic compounds present in different solvents used in extraction. (**B**) shows the relations of phenolic acids among the solvents (**C**) shows the relations of flavonoids present in different solvents. (**D**) shows the other polyphenols present in solvents.

The Venn diagrams (Figure 3) were developed to illustrate the number of phenolic compounds extracted using different solvents, including 70% ethanol (yellow), absolute ethyl acetate (green), 70% methanol (red), and 70% acetone (blue). In the Venn diagram of total phenolics (Figure 3A) extracted using different solvents were 2 (0.4%), 4 (0.9%), 13 (2.8%), and 85 (18.2%), of the unique compounds in absolute ethyl acetate, 70% methanol, 70% acetone, and 70% ethanol, respectively. A study supported our results showed ethanol extracted a higher amount of phenolic compounds, when compared with methanol or acetone [45]. The reason might be because ethanol has higher polarity and so improved phenolic solubility [46]. In another study, 80% methanol and 80% ethanol exhibited higher phenolic content [47]. The overlapped compounds among the 4 solvents were 189 (40.6%) compounds. A total of 102 (21.9%) compounds were common among 70% methanol, 70% ethanol, and 70% acetone. The lowest compounds were overlapped with 70% ethanol and absolute ethyl acetate. The phenolic acids that were common between 4 solvents were 37 (26.8%) compounds (Figure 3B). The unique compounds were only high in 70% ethanol solvent consisting of 54 (39.1%) compounds. A total of 18 (13%) compounds were shared between 70% ethanol, 70% methanol, and 70% acetone. A total of 12 (8.7%) compounds were shared between 70% acetone and 70% methanol. The overlapped flavonoids (Figure 3C) among the solvent were 76 (32.2%) compounds. The unique compounds were 3 (1.3%), 9 (3.8%), 19 (8.1%) and 38 (16.1%), in 70% methanol, 70% acetone, absolute ethyl acetate and 70% ethanol, respectively. Flavonoids of 64 compounds (27.1%) were common between 70% ethanol, 70% methanol, and 70% acetone. Another study reported that the ethanol exhibited high flavonoid content and extracted lower levels with acetone, which is consistent with the results of our study [48]. In other polyphenols (Figure 3D), the unique compounds were 7 (4.6%), 9 (5.9%) and 23 (15.1%), in 70% ethanol, absolute ethyl acetate, and 70% acetone, respectively. The overlapped compounds were 60 (39.5%) among the 4 solvents. A total of 27 (17.8%) compounds were shared among the 70% ethanol, 70% methanol, and 70% acetone.

**Figure 4:**
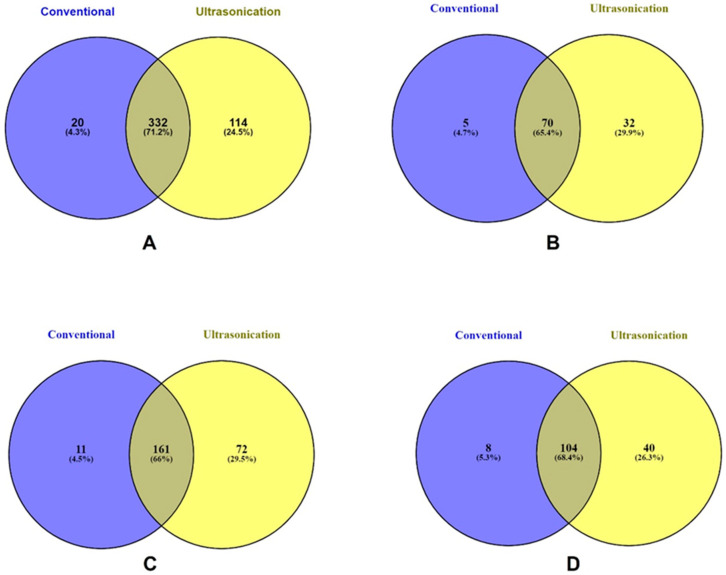
Venn diagram of phenolic compounds extracted via the methodologies of ultrasonication and conventional. (**A**) shows the relations of total polyphenols in different methodologies. (**B**) shows relations of phenolic acids in different methodologies. (**C**) shows relations of flavonoids in different methodologies. (**D**) shows relations of other polyphenols in different methodologies.

The Venn diagrams (Figure 4) were developed according to the number of phenolic compounds extracted via the methodologies of conventional (blue) and ultrasonication (yellow). In the Venn diagram (Figure 4A), the overlapped compounds of conventional and ultrasonication were 332 (71.2%). The unique phenolic compounds were 20 (4.3%) and 114 (24.5%) in conventional and ultrasonication. The phenolic acids (Figure 4B) common to both methodologies were 70 (65.4%) compounds. The phenolic acids unique to conventional and ultrasonication were 5 (4.7%) and 32 (29.9%), respectively. The overlapped flavonoids (Figure 4C) were 161 (66%), unique flavonoids were 11 (4.5%), and 72 (29.5%) in conventional and ultrasonication extraction, respectively. A previous study observed flavonoids were high after ultrasonication extraction, which is consistent with our results [49]. The other polyphenols (Figure 4D) in conventional and ultrasonication were 8 (5.3%) and 40 (26.3%), respectively. However, the unique compounds of total polyphenols were 104 (68.4%) compounds. Ultrasonication methodology extracts low molecular weight compounds by disrupting cell walls and transfer mass of the phenolics. This might be one of the reasons that our study also observed high phenolic compound extracted by ultrasonication [50]. 

**Figure 5:**
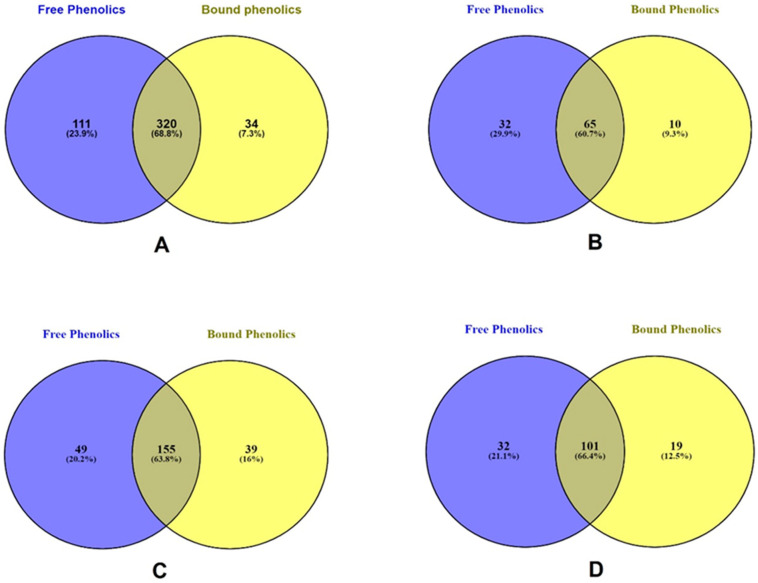
Venn diagram presented in free and bound extraction. (**A**) shows the relations of total phenolic compounds present in free and bound phenolics (**B**) shows the relation of phenolic acids in free and bound phenolics (**C**) shows the relation of flavonoids in free and bound phenolics. (**D**) shows the relation of other polyphenols in free and bound phenolics.

In Figure 5A, the Venn diagram of the bound and free total phenolics of the overlapped phenolics were 320 (68.8%), whereas the bound and free unique total phenolics were 34 (7.3%) and 111 (23.9%), respectively. The overlapped phenolic acids (Figure 5B) were 65 (60.7%). However, the bound and free phenolic acids were 10 (9.3%) and 32 (29.9%) compounds, respectively. The overlapped flavonoids (Figure 5C) were 155 (63.8%) whereas the unique bound and free were 39 (16%) and 49 (20.2%) compounds, respectively. The other polyphenols (Figure 5D) in bound and free unique compounds were 19 (12.5%) and 32 (21.1%), respectively. However, the overlapped compounds were 101 (66.4%). Harukaze et al. [51] reported higher bound phenolics than the free phenolics.

### 2.5. LC-ESI-QTOF-MS/MS Characterization of Phenolic Compounds

LCMS/MS has been widely used in the identification and characterization of the phenolic compound present in the marine seaweed [52]. Qualitative analysis of the phenolic compounds was performed via LC-ESI-QTOF-MS/MS in both positive and negative modes of ionization. Compounds with mass error < ±5 ppm and PCDL library score more than 80 were selected for further MS/MS identification and *m*/*z* characterization purposes. In the present work, the MS/MS was performed and 94 and 104 compounds were identified in extracts from ultrasonication and conventional methodology, respectively (Table 4 and Table 5), which is combined data for both free and bound phenolic compounds (Tables S5 and S6). The phenolic acids present in extracts from ultrasound and conventional methodology were 34 and 33 compounds, respectively. The flavonoids were 43 and 52 in ultrasonication and conventional, respectively. The anthocyanins, a sub-class of flavonoids, were only observed in conventional extraction methods. In other polyphenols, 17 and 19 compounds were identified after MS^2^ in ultrasonication and conventional, respectively. 

Gallic acid ([M – H]^−^, *m*/*z* 169.0154), 2-hydroxybenzoic acid ([M – H]^−^, *m*/*z* 137.0238) and 2,3-dihydroxybenzoic acid ([M – H]^−^, *m*/*z* 153.0190) were identified at product ions *m*/*z* 125, *m*/*z* 93 and at *m*/*z* 109 due to the corresponding loss of CO_2_ [53,54]. Biosynthesis of gallic acid is formed from 3-dehydroshikimate in the presence of shikimate dehydrogenase enzyme to produce 3,5-didehydroshikimate. Further, the 3,5-didehydroshikimate compound rearranges the structure spontaneously to form gallic acid [55]. In the ultrasonication method, *Cystophora* sp. (bound phenolics of ethyl acetate extract) detected the presence of 2,3-dihydroxybenzoic acid while for gallic acid was identified in free and bound forms of phenolics in *Phyllospora comosa* (70% acetone, 70% ethanol, 70% methanol extract), *Ecklonia radiata* (70% acetone, 70% ethanol, absolute ethyl acetate, 70% methanol extract), *Sargassum* sp. (absolute ethyl acetate extract), and *Cystophora* sp. (absolute ethyl acetate, 70% ethanol, 70% acetone extract), whereas 2-hydroxybenzoic acid was present in bound phenolics of *Sargassum* sp. (acetone, methanol extract). In conventional methodology, *Ecklonia radiata* detected the compounds 2-hydroxybenzoic acid and 2,3-dihydroxybenzoic acid in bound phenolics, while gallic acid was identified in *Phyllospora comosa*, *Ecklonia radiata*, *Durvillaea* sp., and *Sargassum* sp. in free form of phenolics. However, *Phyllospora comosa* (70% acetone extract) and *Sargassum* sp. (70% ethanol extract) detected gallic acid in bound form as well. Previously, gallic acid was detected in *Himanthalia elongata* (Phaeophyceae) and *Ulva intestinalis* (Chlorophyta) [56,57]. Seaweeds including *Gracilaria birdiae* and *Gracilaria cornea* (Rhodophyta) collected along the Brazilian shorelines detected the presence of gallic acid. Gallic acid was also detected in other plants including green teas, bearberry leaves, hazelnuts, evening primrose grape seeds [58], and fruit pulp of *Terminalia chebula* [59]. Gallic acid is known for its anticancer, anti-inflammatory, anti-melanogenic, and antioxidant properties [60]. The 2-hydroxybenzoic acid was previously detected from lucerne, hops, berries, Keitt and Kensington Pride mangoes [61]. The 2-hydroxybenzoic acid is a key ingredient in the skin care industry and is used to treat psoriasis, keratosis pilaris, acne, corns, calluses, and warts [62]. The 2,3-dihydroxybenzoic acid was previously detected in *Catharanthus roseus*, wild jujube fruit, wild olive fruit, wild common fig fruit, apple, grapes, kiwi fruit, nectarine, peach, orange, pineapple, plum, and passionfruit peels [63].

Ferulic acid was tentatively identified by precursor ions [M – H]^−^ *m*/*z* at 193.0516. The compound was confirmed by product ions at *m*/*z* 178, *m*/*z* 149, and *m*/*z* 134, indicating the loss of CH_3_, CO_2_, and CH_3_ with CO_2_ from the precursor ions, respectively [64]. Ferulic acid is an abundant hydroxycinnamic acid, available in free form but linked to the lignin. It acts as a precursor in the plant defence response for the production of phytoalexins, signaling molecules and antimicrobial compounds [65]. In our study, ferulic acid was identified in free form from 70% acetone ultrasonic extract of *Cystophora* sp. Previously, ferulic acid has been detected in some seaweed, including *Bifurcaria bifurcate*, *Ascophyllum nodosum,* and *Fucus vesiculosus* (Phaeophyceae) [66]. Another study detected that *Himanthalia elongata* collected from Ireland contained ferulic acid [67]. Similarly, seeds of coffee, artichoke, peanuts, bamboo shoots, eggplant, soybean, spinach, tomato, radish, broccoli, carrot, avocado, orange, banana, berries, and coffee [68] contain ferulic acid. Ferulic acid exhibits antioxidant, anti-inflammatory, antimicrobial, anti-allergic, anti-thrombosis, and anti-cancer activities [69]. *m*-Coumaric acid ([M – H]^−^ *m*/*z* at 163.0412), was identified at product ions *m*/*z* 119, due to the loss of CO_2_ (44 Da) [64]. In ultrasonication methodology, the compound was detected in bound form in *Durvillaea* sp. (70% acetone, 70% ethanol, 70% methanol), *Sargassum* sp. (70% acetone), and *Cystophora* sp. (70% acetone), whereas in conventional methodology, *Cystophora* sp. (absolute ethyl acetate) only identified the compound in free form. Coumaric acid is present in various berries such as strawberries, peanuts, beers, olive oil, and baru almonds [70]. *m*-Coumaric acid compound have antioxidant capacity [71]. Previously, a study on *m*-coumaric acid reported that the compound reduced glucose and glycated hemoglobin levels and enhanced antioxidant activity [72]. 

Quercetin 3′-*O*-glucuronide ([M – H]^−^ *m*/*z* at 477.0679) had product ion at *m*/*z* 301 in the MS^2^ spectrum due to the loss of glucuronide (176 Da) from the precursor [73]. Myricetin 3-*O*-arabinoside ([M – H]^−^ *m*/*z* at 449.0750) had peaks at *m*/*z* 317 (loss of pentose moiety, 132 Da) which confirmed the identity of myricetin 3-*O*-arabinoside [74]. Quercetin 3′-*O*-glucuronide was identified in samples ultrasonication methodology of bound form in *Durvillaea* sp. (70% acetone), and free form in *Ecklonia radiata* (70% methanol, 70% ethanol, 70% acetone, absolute ethyl acetate). Myricetin 3-*O*-arabinoside in *Durvillaea* sp. (70% ethanol and 70% acetone extract) and *Cystophora* sp. (70% methanol extract). The compounds quercetin 3′-*O*-glucuronide and myricetin 3-*O*-arabinoside were identified in free form in conventional methodology in seaweed samples of *Ecklonia radiata* (70% ethanol and 70% acetone extract). The biosynthesis of quercetin via hydroxylation reaction of dihydrokaempferol forms dihydroquercetin in the presence of the enzyme flavonol 3′-hydroxylase. In the following step of biosynthesis, dihydroquercetin catalyzes in the presence of enzyme flavonol synthase to form quercetin [75]. However, the compound myricetin 3-*O*-arabinoside was detected in American cranberry and highbush blueberry [76] in very limited studies on their biological properties. The compound quercetin was also identified and characterised in *Durvillaea* sp. [77]. 

Sativanone was identified in both modes of ionization and tentatively identified by the precursor ions at *m*/*z* 299.0914 and the product ions at *m*/*z* 284 (M – H – 15, loss of CH_3_ from B-ring) and at *m*/*z* 269 (M – H – 30, loss of two CH_3_) and at *m*/*z* 225 (M – H – 74, loss of two CH_3_ and CO_2_) [78]. It was identified by the samples *Sargassum* sp. (70% ethanol extract) and *Cystophora* sp. (70% methanol, 70% ethanol extract) in their free form by ultrasonication methodology while *Phyllospora comosa* (70% ethanol, 70% acetone, 70% methanol extract), *Ecklonia radiata* (70% ethanol extract), *Durvillaea* sp. (70% acetone extract) and *Cystophora* sp. (70% methanol extract), by conventional methodology in free and bound forms. Previously, restharrow root has been used in traditional medicine identified sativanone [79]. Ethanolic extracts of *Dalbergia odorifera*-treated mice when exposed to UVB significantly reduced ROS levels and the number of senescent cells in the skin [80]. Dalbergin compound was tentatively identified with [M – H]^−^ *m*/*z* at 267.0656 exhibited characteristic fragment ions at *m*/*z* 252 [M – H – CH_3_], *m*/*z* 224 [M – H – CH_3_ – CO] and *m*/*z* 180 [M – H – CH_3_ – CO – CO_2_] [78]. It was identified in *Durvillaea* sp., *Sargassum* sp. in both ultrasonication and conventional methodologies. Dalbergin was first isolated from *Dalbergia odorifera* [81]. Dalbergin is widely used traditionally as an anti-inflammatory, anti-pyretic, analgesic, antioxidant, anti-diabetic, antimicrobial, and anti-cancer agent [82].

Scopoletin ([M – H]^−^ *m*/*z* at 191.0347) was identified by the product ions at *m*/*z* 176 [M – H – CH_3_] and *m*/*z* 147 [M – H – CO_2_] [83]. It was identified in *Ecklonia radiata* (absolute ethyl acetate, 70% ethanol, 70% methanol, 70% acetone) and *Cystophora* sp. (70% ethanol and 70% acetone extract) in ultrasonication methodology whereas in conventional, it was identified in *Ecklonia radiata* (70% ethanol, 70% methanol extract), *Durvillaea* sp. (70% acetone), *Sargassum* sp. (70% ethanol, 70% methanol) and *Cystophora* sp. (70% ethanol, 70% methanol). This compound was previously detected in seaweeds, including *Codium* sp. (Chlorophyta), *Grateloupia* sp. (Rhodophyta), and *Sargassum* sp., (Phaeophyceae) [20]. Scopoletin was also detected in curry plant, cassava, candlenut tree, giant potato, sweet wormwood, chinaberry tree, sugar maple, perfume flower tree, and white mulberry [84]. Scopoletin has been shown to possess antimicrobial properties, reduce inflammations, and decrease cardiovascular diseases [85]. Scopoletin extracted from the *Hypochaeris radicata* demonstrated anti-inflammatory and antioxidant properties by suppressing the production of proinflammatory cytokines such as TNF-α, IL-1β, and IL-6 [86]. 

Resveratrol ([M – H]^−^ *m*/*z* at 227.0717) was detected in both ionization modes. Resveratrol observed the fragmentation ions at *m*/*z* 212 [M – H – CH_3_], *m*/*z* 185 [M – H – CHCOH], *m*/*z* 157 [M – H – CHCOH – CO], and *m*/*z* 143 [M – H – CHCOH – C_2_H_2_O] [87]. It was identified in the seaweeds *Sargassum* sp. (70% acetone, 70% ethanol) and *Cystophora* sp. (70% acetone) in ultrasonication. The compound was previously detected in red wine and contributes to the antioxidant potential and hence may play a role in the prevention of cardiovascular diseases [88].

### 2.6. Heatmap Analysis of Quantified Phenolics in Seaweeds 

**Figure 6:**
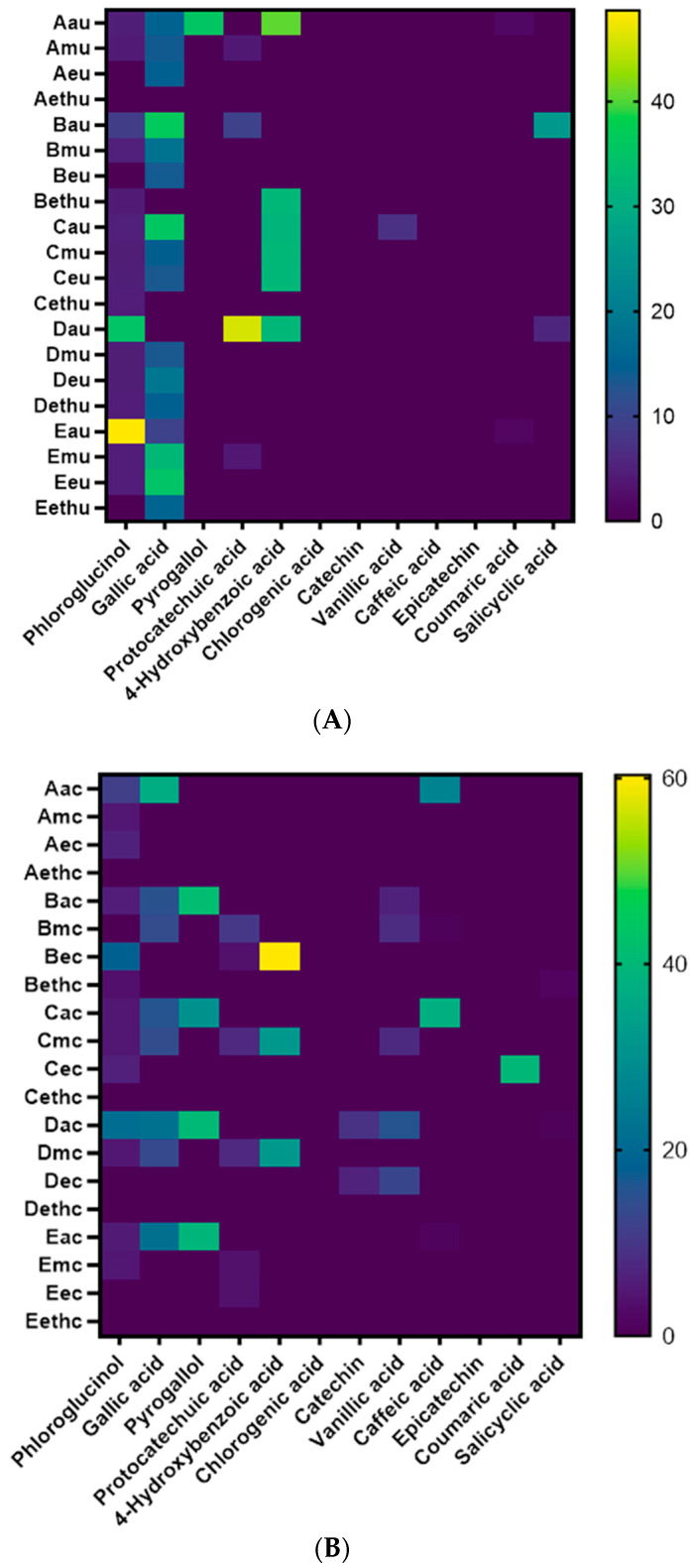
(**A**) Heatmap showing phenolic compounds’ distribution and concentration among five seaweed species extracted in four different solvents via ultrasonication extraction. (**B**) Heatmap showing phenolic compounds’ distribution and concentration among five seaweed species extracted in four different solvents via conventional extraction.

Quantification of phenolic compounds in seaweed has been a topic of research and discussion for many years. In this study, the phenolic compounds were quantified using high performance liquid chromatography connected to photodiode array detector (HPLC-PDA). This method quantifies individual compounds based on their retention time and UV absorption spectra. HPLC-PDA is very specific and accurate when compared to the in vitro assay estimation completed earlier in this study. The heat map (Figure 6A,B) was constructed based on the data of the combined results of both free and bound phenolics in Australian brown beach-cast seaweeds extracted via conventional and ultrasonication methodologies. In this study, twelve phenolics were quantified, including ten phenolic acids and two flavonoids. 

Phloroglucinol extracted via ultrasonication extraction was quantified and shown to be present in high amounts in *Cytosphora* sp. (70% acetone extract), and *Sargassum* sp. (70% acetone extract). However, in conventional extraction, the compound was more abundant in *Sargassum* sp. (70% acetone extract), followed by *Ecklonia radiata* (70% ethanol extract), but lower than obtained using ultrasonication extraction. The differences in phlorotannin levels are probably influenced by species, season, and the site of collection [89]. A study demonstrated that 70% acetone was the highest extracted level of phenolics from the seaweeds, which is consistent with our study [90]. In seaweeds, gallic acid is metabolised via dehydrogenation of 5-dehydroshikimic acid [91]. In our study, we quantified gallic acid and observed that ultrasonication extraction extracted a higher amount of the compound than conventional extraction. This might be due to increase in mass transfer in reduced extraction time in ultrasonication [12]. Pyrogallol was only identified and quantified in *Phyllosphora Comosa* (70% acetone extract) via the ultrasonication methodology, whereas this compound was identified and quantified in *Ecklonia radiata*, *Durvillaea* sp., *Sargassum* sp., and *Cytosphora* sp., extracted via the conventional methodologies. Ultrasonic extraction might partially degrade some targeted compounds due to shear force and temperature, resulting in lower yields for some compounds [92]. Therefore, the conventional extraction may be better for the recovery of compounds sensitive to the higher temperatures whereas shear stress is generated in ultrasonic extraction which results in low recovery of phenolic compounds. Protocatechuic acid is formed from the dehydration of the 3-dehydroshikimic acid in the metabolic pathway of the seaweeds [93]. The compound was higher in ultrasonication of the *Sargassum* sp. (70% acetone extract) when compared to other species and solvents. However, protocatechuic acid compound was not detected in the conventional extraction, illustrating an example where ultrasonic extraction is more effective.

## 3. Materials and Methods

### 3.1. Chemicals

The chemicals used for extraction were methanol, ethanol, ethyl acetate, acetone, and formic acid of analytical grade. For the in vitro assays, the standards used were gallic acid, phloroglucinol, ethylenediaminetetraacetic acid (EDTA), quercetin, 6-hydroxy-2,5,7,8-tetramethylchroman-2-carboxylic acid (Trolox), and catechin, obtained from Sigma Aldrich (St. Louis, MO, USA). The chemicals used for estimation of phenolic and antioxidant potential were 2,2′-diphenyl-1-picrylhydrazl (DPPH), 2,4,6-tripyridyl-s-triazine (TPTZ), Folin–Ciocalteu’s phenol reagent, vanillin, aluminium chloride hexahydrate, ferric (III) chloride anhydrous, potassium persulfate, 2-2′-azino-bis(3-ethylbenz-thiazoline-6-sulphonate) (ABTS), 3-ethylbenzothiazoline-6-sulphonic acid, sodium phosphate dibasic heptahydrate, iron (II) chloride, sodium phosphate monobasic monohydrate, iron (II) sulfate heptahydrate, 3-hydroxybenzoic acid, ferrozine, potassium ferricyanide, 2,4-dimethoxybenzaldehyde (DMBA), ferric ammonium sulfate, potassium ferricyanide, sodium tungstate, absolute ethanol and dodeca-molybdophosphoric acid, purchased from Sigma-Aldrich (Castle Hill, NSW, Australia). Methanol, sodium hydroxide pellets, sodium carbonate anhydrous, and hydrogen peroxide (30%) were purchased from Chem-Supply Pty Ltd. (Adelaide, SA, Australia). Ethyl acetate, sodium acetate (hydrated), formic acid 99%, and acetone were purchased from Chem-Supply Pvt Ltd. and Ajax Finecham, respectively (VIC, Melbourne, Australia). Glacial acetic acid was purchased from Thermo Fisher Scientific Inc (Waltham, MA, USA). Milli-Q water by Millipore Milli-Q Gradient Water Purification System (Darmstadt, Germany). The 98% sulphuric acid was procured from RCI Labscan Ltd. (Bangkok, Thailand).

### 3.2. Seaweed Collection and Identification of Seaweed Samples

**Figure 7:**
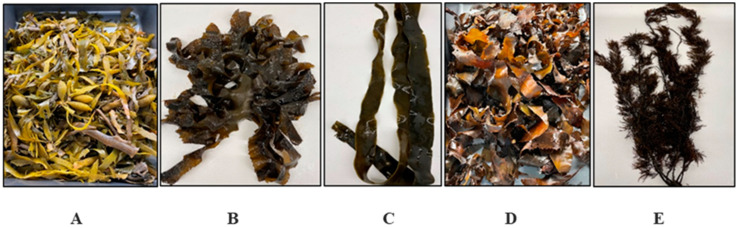
Seaweed samples used in our study, Sample (**A**) *Phyllospora comosa*, Sample (**B**) *Ecklonia radiata*, Sample (**C**) *Durvillaea* sp., Sample (**D**) *Sargassum* sp., Sample (**E**) *Cystophora* sp.

*Phyllospora comosa*, *Ecklonia radiata*, *Durvillaea* sp., *Sargassum* sp., and *Cystophora* sp. (Phaeophyceae) seaweeds (Figure 7) were abundantly found at Queenscliff Harbour (38°15′54.0″ S 144°40′10.3″ E), Victoria, Australia and collected in the month of February (summer). A randomised collection pattern was used without considering the age and size of the seaweed. These seaweed samples were identified at Deakin Marine Institute, Queenscliff, Victoria, Australia.

### 3.3. Sample Preparation

Fresh seaweed samples were thoroughly washed with tap water and subsequently with Milli-Q water to remove any external adhering salts, epiphytes, and other foreign impurities. The seaweed samples were cut manually into smaller pieces about 1–3 cm each with a stainless-steel food-grade knife. The fresh seaweed samples were freeze-dried. The seaweed samples were frozen to −70 °C for 24 h in a Thermo scientific freezer. The frozen samples were placed in the freezer dryer at −60 °C for 72 h as the procedure described in Badmus et al. [94]. The sample was ground using a grinder (Cuisinart Nut and Spice grinder 46302, Melbourne, VIC) to make it into a fine coarse powder. The dried samples were stored in the cold room.

### 3.4. Extraction Preparation

#### 3.4.1. Free Phenolics Extraction

The conventional and ultrasonication of free phenolics were performed and slightly modified as described by Čagalj et al. [95]. The seaweed samples, in triplicate, were extracted with 70% methanol, 70% ethanol, 70% acetone, and absolute ethyl acetate using two extraction methods. All the extraction solvents were added with 0.1% formic acid. The seaweed to solvent ratio was set at 1:20 for all extractions. The following extraction methods were applied: (i) shaking incubator for 16 h at 120 rpm at 10 °C (ZWYR-240 incubator shaker, Labwit, Ashwood, VIC, Australia) (ii) UAE performed with ultrasonicator at 40% amplitude for 5 min. After the extraction, the samples were centrifuged for 15 min at 5000 rpm under 4 °C using Hettich Refrigerated Centrifuge (ROTINA380R, Tuttlingen, Baden-Württemberg, Germany). The supernatant fluid was filtered via 0.45 µm syringe filter (Thermo Fisher Scientific Inc., Waltham, MA, USA) and collected as free phenolic extracts. The sample residues were air-dried for 72 h. The residues were washed with their solvents 3 times and the residue was then further analysed for bound phenolics. 

#### 3.4.2. Bound Phenolic Extraction

The conventional and ultrasonication of bound phenolics were performed and slightly modified as described by Gulsunoglu et al. [96]. The seaweed samples, in triplicate, were extracted with 70% methanol, 70% ethanol, 70% acetone, and absolute ethyl acetate. All solvents were added with 0.1% formic acid. The residue was added with 10 mL 2 N NaOH in a screw-capped test tube. For conventional extraction, the sample was neutralised (pH 7) with 2 N HCl and dosed with 10 mL of respective solvents. The samples were incubated in a shaking incubator for 16 h at 120 rpm at 4 °C (ZWYR-240 incubator shaker, Labwit, Ashwood, VIC, Australia). For ultrasonication, the sample was sonicated at 40% amplitude for 5 min and neutralised (pH 7) with 2 N HCl. Later, the sonicated sample was dosed with 10 mL of respective solvents. The samples were centrifuged for 15 min at 5000 rpm under 4 °C using Hettich Refrigerated Centrifuge (ROTINA380R, Tuttlingen, BadenWürttemberg, Germany). The supernatant fluid was filtered via 0.45 µm syringe filter (Thermo Fisher Scientific Inc., Waltham, MA, USA) and collected as bound phenolic extracts.

### 3.5. Estimation of Phenolic and Antioxidant Assays

The assays were performed according to the published methods of Subbiah et al. [97] and Suleria, Barrow, and Dunshea [63] for phenolic estimation of free and bound phenolics (TPC, TFC, DMBA, PBA, and FDA) and their total antioxidant potential (DPPH, FRAP, ABTS, ·OH-RSA, FICA, RPA, and TAC) extracted via conventional and ultrasonication methodologies. Multiskan^®^ Go microplate photometer (Thermo Fisher Scientific Inc., Waltham, MA, USA) were used to attain the absorption data.

#### 3.5.1. Estimation of Total Phenolic Compound

The total phenolic content of free and bound extracted through conventional and ultrasonication methodologies were estimated by Folin–Ciocalteu’s method as described in Mussatto et al. [98]. An amount of 25 µL extract, 25 µL Folin–Ciocalteu’s reagent solution (1:3 diluted with water) and 200 µL water were added to the 96-well plate (Costar, Corning, NY, USA). The 96-well plate was incubated in the darkroom for 5 min at room temperature (~25 °C). An amount of 25 µL of 10% (*w*:*w*) sodium carbonate was added to the reaction mixture and incubated at 25 °C for 60 min. Absorbance was measured at 765 nm using a spectrophotometer (Thermo Fisher Scientific, Waltham, MA, USA). Concentrations ranging from 0 to 200 µg/mL of gallic acid were prepared as a standard curve and the TPC content was expressed in mg of gallic acid equivalents per gram based on dry weight (mg GAE/g of the sample).

#### 3.5.2. Determination of Total Flavonoid Compounds (TFC)

The quantification of TFC was completed by using the aluminum chloride method with slight modification as described in Ali et al. [99]. The extract of the phenolic compounds were extracted through conventional and ultrasonication methodologies. An amount of 80 µL extract followed by 80 µL of aluminum chloride and 120 µL of 50 g/L sodium acetate solution were added to the 96-well plate. The 96-well plate was incubated for 2.5 h in the darkroom. Absorbance was measured at 440 nm. The concentration ranging from 0–50 µg/mL for the quercetin calibration curve was used to determine TFC and expressed in mg quercetin equivalents per gram of sample (mg QE/g _d.w._).

#### 3.5.3. Determination of Total Tannin Content (TTC)

The total tannin content was determined by the vanillin sulfuric acid method as described by Ali, Wu, Ponnampalam, Cottrell, Dunshea and Suleria [99] with slight modification. As previously mentioned, the phenolic compounds were extracted through conventional and ultrasonication methodologies. An amount of 25 µL of sample extract followed by 25 µL of 32% sulfuric acid and 150 µL of 4% vanillin solution was added to a 96-well plate and incubated for 15 min in the darkroom. The absorbance was measured at 500 nm. Catechin calibration curve with concentration from 0 to 1 mg/mL was used for estimation of TCT and expressed in mg catechin equivalents (CE) per g of sample weight (mg CE/g d.w.).

#### 3.5.4. 2,4-Dimethoxybenzaldehyde Assay (DMBA)

The total phlorotannin content was estimated using the 2,4-dimethoxybenzaldehyde (DMBA) assay as described in Vissers et al. [100]. An amount of 2% of DMBA was added in acetic acid (*m/v*) and 6% hydrochloric acid in acetic acid (*v/v*). Both solutions were mixed at equal volumes to make DMBA solution. An amount of 25 µL of sample and 125 µL of DMBA solution were added into the 96-well microplate. The reaction mixture was incubated in the dark at 25 °C for 60 min. The absorbance was read at 510 nm. The standard curve was prepared to estimate total phlorotannin of phloroglucinol (0–25 µg/mL) and expressed in mg phloroglucinol equivalents per gram (mg PGE/g _d.w_).

#### 3.5.5. Prussian Blue Assay (PBA)

To estimate the total phlorotannin content, the methodology was first suggested by Stern et al. [101] and modified according to Margraf et al. [102]. A diluted sample of 50 µL was added to 50 µL of ferric ammonium sulfate (0.1 M FeNH_4_(SO4)_2_ in 0.1 M HCl) and the reaction mixture was kept in dark for 2 min and an addition of 50 µL of potassium ferricyanide [0.008 M K_3_Fe(CN)_6_]. It was incubated in a dark room for 15 min and absorbance recorded at 725 nm. The standard curve was prepared to estimate total phlorotannin of phloroglucinol (0–3.125 µg/mL) and expressed in mg phloroglucinol equivalents per gram (mg PGE/g _d.w_).

#### 3.5.6. Folin–Denis Assay (FDA)

Phlorotannin assay was determined by Folin–Denis assay as described in Stern, Hagerman, Steinberg, Winter and Estes [101]. The reagent of Folin–Denis was prepared by dissolving 25 g of sodium tungstate (Na_2_WO_4_·2H_2_O) and 5 g of dodeca-molybdophosphoric acid (12MoO_3_·H_3_PO_4_·H_2_O) in 175 mL distilled water, adding 12.5 mL phosphoric acid to the solution, boiling under reflux for 2 h, and then makeup to 250 mL. An amount of 5 µL of the sample was mixed with 20 µL of Folin–Denis reagent, 40 µL of saturated sodium carbonate, and 125 µL of water. The reaction mixture was incubated in the dark for 2 h and the absorbance was read at 725 nm. The standard curve estimates the total phlorotannin of phloroglucinol (0–100 µg/mL) and is expressed in mg phloroglucinol equivalents per gram (mg PGE/g _d.w_). 

#### 3.5.7. 2,2′-Diphenyl-1-Picrylhydrazyl (DPPH) Assay

The estimation of free radical scavenging activity of the seaweed by the DPPH method was performed as described by Nebesny and Budryn [103] with slight modification. To prepare the DPPH radical solution, dissolve 4 mg of DPPH in 100 mL of analytical-grade methanol. An amount of 40 µL of extract and 260 µL of DPPH solution were added to a 96-well plate and vigorously shaken in the dark for 30 min at 25 °C. The absorbance was measured at 517 nm. Trolox standard curve with a concentration ranging from 0 to 200 µg/mL was used to determine the DPPH radical scavenging activity and expressed in mg of Trolox equivalent per gram (mg TE/g _d.w._) of the sample.

#### 3.5.8. Ferric Reducing Antioxidant Power (FRAP) Assay

This assay has been used to estimate the antioxidant capacity in marine seaweeds with some modifications as described by Benzie and Strain [104]. To prepare the FRAP dye, 20 mM Fe [III] solution, 10 mM TPTZ solution, and 300 mM sodium acetate solution were mixed at a ratio of 1:1:10. An amount of 20 µL of the extract and 280 µL prepared dye were added to a 96-well plate and incubated at 37 °C for 10 min. The absorbance was measured at 593 nm. Trolox standard curve with concentration ranging from 0 to 100 µg/mL was used to determine the FRAP values and expressed in mg of Trolox equivalent per gram of sample (mg TE/g _d.w_.).

#### 3.5.9. 2,2′-Azino-Bis-3-Ethylbenzothiazoline-6-Sulfonic Acid (ABTS) Assay

ABTS radical cation decolorization assay was used to determine the free radical scavenging activity of the marine seaweed samples with few modifications as described in Re et al. [105]. An amount of 88 µL of 140 mM potassium persulfate and 5 mL of 7 mM ABTS solution were added to prepare the ABTS^+^ stock solution and were incubated in the darkroom for 16 h. An amount of 10 µL of the extract and 290 µL dye solution were added to the 96-well plate and incubated at 25 °C for 6 min. The absorbance was measured at 734 nm. The antioxidant potential was calculated using the standard curve of Trolox with (0–500 µg/mL) and was expressed in Trolox (TE) in mg per gram of sample.

#### 3.5.10. Estimation of Hydroxyl Radical Scavenging Activity (OH-RSA)

The hydroxyl radical scavenging activity of marine seaweed was determined with the modification of the method Smirnoff and Cumbes [106]. An amount of 50 μL sample extract, 50 μL 6 mM hydrogen peroxide, and 50 μL 6 mM ferrous sulfate heptahydrate were injected into the plate and incubated at 25 °C for 10 min. To the reaction mixture, 50 μL of 6 mM 3-hydroxybenzoic acid was added. Trolox (0–400 μg/mL) was used for calibration and the absorbance was measured at 510 nm.

#### 3.5.11. Estimation of Ferrous Ion Chelating Activity (FICA)

FICA assay was performed as described by Dinis et al. [107] with slight modifications. An amount of 15 μL sample extract, 85 μL water, 50 μL 2 mM ferrous chloride, and 50 μL of 5 mM ferrozine were added to a 96-well plate. The reaction mixture was incubated for 10 min in the dark at 25 °C. The standard curve of EDTA (0–50 μg/mL) was prepared and the absorbance was measured at 562 nm. The results were expressed as mg EDTA equivalents per dry weight (mg EDTA/g _d.w_).

#### 3.5.12. Estimation of Reducing Power (RPA)

The RPA assay was modified and performed according to the method of Ferreira et al. [108]. An amount of 10 μL sample extract, 25 μL 1% potassium ferricyanide (III) solution, and 25 μL 0.2 M phosphate buffer (pH 6.6) were added to a 96-well plate. The reaction mixture was incubated for 20 min at 25 °C. To the reaction mixture, 25 μL of 10% trichloroacetic acid was added to stop the reaction followed by the addition of 85 μL water and 8.5 μL 0.1% ferric chloride solution. It was incubated for 15 min at 25 °C. A standard curve of Trolox (0–500 μg/mL) was used for the calibration curve and absorbance was measured at 750 nm and the results were expressed as mg TE/g ± SD.

#### 3.5.13. Total Antioxidant Capacity (TAC)

The total antioxidant capacity was estimated by the phosphomolybdate method as described in Prieto et al. [109]. An amount of 0.028 M sodium phosphate, sulphuric acid (0.6 M), and 0.004 M ammonium molybdate were mixed to form phosphomolybdate reagent. An amount of 40 µL extract and 260 µL of phosphomolybdate reagent were added to the 96-well plate. The reaction mixture was incubated at 90 °C for 90 min. The absorbance was measured at 695 nm upon the reaction mixture cooling down to room temperature. TAC was determined by using the Trolox standard curve (0–200 μg/mL) and expressed in mg Trolox equivalents (TE) per g of the dry sample weight.

### 3.6. Characterization of Phenolic Compounds by LC-ESI-QTOF-MS/MS Analysis

LC-ESI-QTOF-MS/MS carried out the extensive characterization of phenolic compounds using the method described by Allwood et al. [110] and Zhu et al. [111]. The phenolic compounds from five different species of Australian beach-cast seaweeds were extracted via conventional and ultrasonication methodologies. An Agilent 1200 series of HPLC (Agilent Technologies, Santa Clara, CA, USA) connected via electrospray ionization source (ESI) to the Agilent 6530 Accurate-Mass Quadrupole Time-of-Flight (Q-TOF) LC/MS (Agilent Technologies, Santa Clara, CA, USA). HPLC buffers were sonicated using a 5 L Digital Ultrasonic water bath (Power sonic 505, Gyeonggi-do, Republic of Korea) for 10 min at 25 °C. The separation was carried out using a Synergi Hydro-Reverse Phase 80 Å, LC column 250 × 4.6 mm, 4 µm (Phenomenex, Torrance, CA, 202 USA) with temperature 25 °C and sample temperature at 10 °C. The sample injected was 20 µL. Since the system was binary solvent: mobile phase A, 100% MilliQ water added with 0.1% formic acid, and mobile phase B, acetonitrile/MilliQ water/formic Acid (95:5:0.1), at a flow rate of 0.3 mL/min. The gradient was as follows: 0–2 min hold 2% B, 2–5 min 2–5% B, 5–25 min 5–45% B; 25–26 min 45–100% B, 26–29 min hold 100% B, 29–30 min 100–2% B, 30–35 min hold 2% B for HPLC equilibration. Both positive and negative modes were applied for peak identification. Nitrogen gas has been used as a nebulizer and drying gas at 45 psi, with a flow rate of 5 L/min at 300 °C. Capillary and nozzle voltage were placed at 3.5 kV and 500 V, respectively, and the mass spectra were obtained at the range of 50–1300 amu. Further, MS/MS analyses were carried out in automatic mode with collision energy (10, 15, and 30 eV) for fragmentation. Data acquisition and analyses were performed using Agilent LC-ESI-QTOF-MS/MS Mass Hunter workstation software (Qualitative Analysis, version B.03.01, Agilent).

### 3.7. HPLC-PDA Analysis

The targeted phenolic compounds present in seaweeds were quantified by Agilent 1200 series HPLC (Agilent Technologies, CA, USA) equipped with a photodiode array (PDA) detector according to our previously published protocol of Gu et al. [112] and Suleria, Barrow, and Dunshea [63]. The sample’s phenolic compounds were extracted by conventional and ultrasonication. Sample extracts were filtered by the 0.45 μm syringe filter (PVDF, Millipore, MA, USA). A Synergi Hydro-RP (250 × 4.6 mm i.d.) reversed-phase column with a particle size of 4 µm (Phenomenex, Lane Cove, NSW, Australia) was protected by a Phenomenex 4.0 × 2.0 mm i.d., C18 ODS guard column. The injection volume of the sample or standard was 25 μL. The mobile phase A and B were of water/acetic acid (98:2, *v*/*v*) and acetonitrile/water/acetic acid (50:50:2, *v*/*v*/*v*), respectively. The gradient profile was 90–10% B (0–20 min), 75–30% B (20–30 min), 65–35% B (30–40 min), 45–55% B (40–60 min), 90–10% B (60–61 min), 90–10% B (61–66 min). The flow rate was 0.8, and the column was operated at room temperature. The wavelengths of 280, 320, and 370 nm were simultaneously selected at the PDA detector. Empower Software (2010) was used for instrument control, data collection, and chromatographic processing.

### 3.8. Statistical Analysis

All the analyses were performed in triplicates and the results are presented as mean ± standard deviation (*n* = 3). The mean differences between different seaweed samples were analysed by one-way analysis of variance (ANOVA) and Tukey’s honestly significant differences (HSD) multiple rank test at *p* ≤ 0.05. ANOVA was carried out via Minitab 19.0 software for windows. For correlations between polyphenol content and antioxidant activities, Pearson’s correlation coefficient at *p* ≤ 0.05 and multivariate statistical analysis including a principal component analysis (PCA), XLSTAT-2019.1.3 were used by Addinsoft Inc., New York, NY, USA.

## 4. Conclusions

According to our study, it was found that among the five species of seaweed, *Cystophora* sp. displayed higher total phenolic and phlorotannin content, as well as antioxidant potential (DPPH, FRAP, ABTS, and TAC). On the other hand, *Durvillaea* sp. had high flavonoid content but less antioxidant potential. The ultrasonication had extracted higher phenolic and had high antioxidant potential in the solvents of 70% acetone and 70% methanol. The Venn diagram demonstrated high unique compounds in *Sargassum* sp., and solvent 70% ethanol extracted high unique compounds. In the present work, the MS/MS analysed 94 and 104 compounds in ultrasonication and conventional methodology. The ultrasound and conventional compounds had 72 common compounds whereas the bound and free phenolics had 49 common compounds. The quantification by HPLC showed the quantity of phenolic compounds present, the phenolic acids (phloroglucinol and gallic acid) were higher in ultrasonication when compared to conventional methodology. The findings of the phenolic compounds will further help us in quantifying the compounds and further analysis for the in vitro bio accessibility. 

## Figures and Tables

**Table 1:** Estimation of freeze-dried total phenolics of seaweed species extracted by the conventional and non-conventional method of ultrasonication.

Samples	Solvents	TPC (mg GAE/g)	TFC (mg QE/g)	TCT (mg CE/g)	DMBA (PGE mg/g)	PBA (PGE mg/g)	FDA (PGE mg/g)
Ultrasonication Extraction							
*Cystophora* sp.	70% ACE	19.35 ± 0.09 ^Ba^	2.28 ± 0.02 ^HIb^	0.67 ± 0.06 ^EFc^	1.96 ± 0.08 ^Fb^	2.61 ± 0.30 ^Je^	11.21 ± 0.7 ^Ba^
	70% MeOH	22.74 ± 0.28 ^Ba^	3.33 ± 0.02 ^E–Gb^	-	1.02 ± 0.04 ^Ia^	8.61 ± 0.08 ^Bb^	3.86 ± 0.1 ^Hc^
	70% EtOH	28.92 ± 1.33 ^Aa^	3.82 ± 0.07 ^DEa^	2.21 ± 0.01 ^Ba^	1.63 ± 0.02 ^Ga^	8.11 ± 0.61 ^BCa^	14.2 ± 0.63 ^Aa^
	EA	1.66 ± 0.09 ^Ob^	7.13 ± 0.9 ^Cb^	0.94 ± 0.07 ^DEa^	4.15 ± 0.01 ^Dc^	5.05 ± 0.25 ^Ha^	-
*Phyllospora comosa*	70% ACE	16.11 ± 0.85 ^Fb^	1.86 ± 0.01 ^IJc^	0.94 ± 0.07 ^DEb^	4.96 ± 0.44 ^Ca^	6.18 ± 0.29 ^DEFb^	8.57 ± 0.89 ^Cb^
	70% MeOH	16.85 ± 0.12 ^Db^	3.43 ± 0.06 ^D–Fb^	2.86 ± 0.12 ^Aa^	0.67 ± 0.01 ^JKb^	12.62 ± 0.26 ^Aa^	6.58 ± 0.17 ^Ea^
	70% EtOH	12.41 ± 0.03 ^Hc^	3.62 ± 0.03 ^D-Fb^	2.18 ± 0.06 ^Ba^	0.46± 0.01 ^LMd^	3.2 ± 0.22 ^Ic^	6.36 ± 0.41 ^Fb^
	EA	2.23 ± 0.04 ^Pd^	3.54 ± 0.10 ^D–Fc^	0.77 ± 0.06 ^Eb^	6.65 ± 0.67 ^Bb^	0.13 ± 0.6 ^Lc^	-
*Sargassum* sp.	70% ACE	14.6 ± 0.72 ^Gc^	0.67 ± 0.04 ^Le^	0.27 ± 0.02 ^Gd^	0.89 ± 0.05 ^Id^	6.5 ± 0.07 ^DEFa^	7.64 ± 0.25 ^Dc^
	70% MeOH	16.36 ± 0.34 ^Ec^	1.39 ± 0.01 ^JKc^	-	0.68 ± 0.04 ^JKb^	7.77 ± 0.16 ^Cc^	5.24 ± 0.37 ^Gb^
	70% EtOH	17.52 ± 0.57 ^Cb^	2.79 ± 0.02 ^GHd^	0.64 ± 0.03 ^EFc^	0.72 ± 0.11 ^Jc^	6.85 ± 0.19 ^Db^	3.39 ± 0.25 ^Jd^
	EA	0.34 ± 0.03 ^Qe^	2.6 ± 0.04 ^Hd^	-	3.24 ± 0.34 ^Ed^	1.18 ± 0.3 ^Kb^	-
*Ecklonia radiata*	70% ACE	11.19 ± 0.41 ^Id^	0.85 ± 0.01 ^KLd^	1.29 ± 0.01 ^CDa^	1.02 ± 0.1 ^Ic^	5.86 ± 0.25 ^FGc^	2.5 ± 0.04 ^Le^
	70% MeOH	12.19 ± 0.41 ^Id^	11.15 ± 0.12 ^Ba^	0.92 ± 0.09 ^DEc^	0.96 ± 0.07 ^a^	8 ± 0.37 ^BCc^	3.5 ± 0.1 ^IJd^
	70% EtOH	9.03 ± 0.11 ^Kd^	1.65 ± 0.04 ^Je^	-	1.18 ± 0.02 ^Hb^	6.13 ± 0.14 ^EFb^	0.71 ± 0.01 ^Me^
	EA	1.54 ± 0.01 ^Oc^	4.04 ± 0.01 ^Dc^	-	8.29 ± 0.47 ^Aa^	0.96 ± 0.13 ^Kb^	-
*Durvillaea* sp.	70% ACE	9.98 ± 0.68 ^Je^	3.35 ± 0.03 ^EFa^	1.04 ± 0.01 ^Db^	0.57 ± 0.02 ^KLe^	5.36 ± 0.14 ^GHd^	6.72 ± 0.26 ^Ed^
	70% MeOH	4.45 ± 0.09 ^Ne^	1.32 ± 0.03 ^JKc^	2.12 ± 0.07 ^Bb^	0.35 ± 0.02 ^Mc^	3.87 ± 0.47 ^Id^	2.82 ± 0.25 ^Ke^
	70% EtOH	8.6 ± 0.45 ^Le^	3.15 ± 0.01 ^FGc^	1.9 ± 0.05 ^Cb^	0.4 ± 0.01 ^Md^	6.63 ± 0.58 ^DEb^	3.63 ± 0.3 ^Ic^
	EA	7.26 ± 0.03 ^Ma^	18.23 ± 0.17 ^Aa^	-	8.22 ± 0.18 ^Aa^	0.48 ± 0.04 ^KLb^	-
Conventional Extraction							
*Cystophora* sp.	70% ACE	18.27 ± 1.06 ^Ba^	2.46 ± 0.03 ^Da^	0.15 ± 0.01 ^FGc^	0.57 ± 0.03 ^HIa^	0.48 ± 0.21 ^Ke^	7.17 ± 0.55 ^Db^
	70% MeOH	18.59 ± 0.54 ^Aa^	1.83 ± 0.01 ^Ea^	2.55 ± 0.07 ^Ba^	0.94 ± 0.26 ^Fb^	7.35 ± 0.1 ^Aa^	8.94 ± 0.42 ^Ba^
	70% EtOH	15.49 ± 0.59 ^Ca^	2.48 ± 0.04 ^Da^	1.40 ± 0.07 ^Da^	0.79 ± 0.14 ^Gb^	6.2 ± 0.25 ^Cb^	8.44 ± 0.55 ^Ca^
	EA	0.96 ± 0.05 ^La^	2.51 ± 0.15 ^Cc^	0.17 ± 0.02 ^FGb^	-	-	-
*Sargassum* sp.	70% ACE	13.93 ± 0.45 ^D^	0.34 ± 0.01 ^Ke^	0.28 ± 0.03 ^EFb^	0.65 ± 0.01 ^Ha^	4.74 ± 0.15 ^Ea^	3.05 ± 0.13 ^Gd^
	70% MeOH	10.37 ± 0.63 ^Fc^	1.38 ± 0.02 ^Kb^	0.43 ± 0.11 ^Ec^	0.54 ± 0.03 ^HIc^	7.03 ± 0.08 ^Bb^	2.91 ± 0.19 ^Hb^
	70% EtOH	8.89 ± 0.19 ^Gc^	0.96 ± 0.06 ^Kd^	0.13 ± 0.01 ^FGc^	0.58 ± 0.01 ^HIc^	3.7 ± 0.06 ^Fd^	0.69 ± 0.2 ^Kd^
	EA	0.53 ± 0.03 ^Mc^	6.35 ± 0.01 ^Bb^	0.02 ± 0.01 ^Gc^	6.32 ± 0.8 ^Bb^	0.12 ± 0.06 ^Lc^	-
*Phyllospora comosa*	70% ACE	13.72 ± 0.50 ^Db^	1.07 ± 0.02 ^Fb^	0.59 ± 0.01 ^Hd^	0.38 ± 0.01 ^JKb^	2.26 ± 0.35 ^Id^	1.87 ± 0.12 ^Je^
	70% MeOH	11.03 ± 0.81 ^Eb^	0.22 ± 0.01 ^Lc^	0.14 ± 0.05 ^EFd^	0.33 ± 0.01 ^Kd^	1.48 ± 0.22 ^Jd^	1.92 ± 0.01 ^Jd^
	70% EtOH	10.28 ± 0.59 ^Fb^	0.91 ± 0.02 ^Gb^	-	0.47 ± 0.03 ^IJd^	6.95 ± 0.09 ^Ba^	4.69 ± 0.2 ^Eb^
	EA	0.68 ± 0.01 ^Mc^	6.52 ± 0.03 ^Aa^	-	6.01 ± 0.01 ^Cc^	2.22 ± 0.2 ^Ia^	-
*Ecklonia radiata*	70% ACE	10.78 ± 0.31 ^Gc^	0.35 ± 0.03 ^Jd^	-	0.59 ± 0.03 ^HIa^	2.6 ± 0.05 ^Hc^	3.6 ± 0.3 ^Fc^
	70% MeOH	10.63 ± 0.38 ^Hd^	0.12 ± 0.04 ^Lc^	-	1.12 ± 0.12 ^Ea^	1.46 ± 0.1 ^Jd^	2.5 ± 0.04 ^Ic^
	70% EtOH	8.74 ± 0.21 ^Id^	0.34 ± 0.05 ^Gb^	-	0.91 ± 0.07 ^FGa^	4.94 ± 0.7 ^Dc^	2.82 ± 0.02 ^Hc^
	EA	-	0.11 ± 0.02 ^Aa^	27.54 ± 0.23 ^Aa^	6.57 ± 0.3 ^Aa^	0.46 ± 0.3 ^Kb^	-
*Durvillaea* sp.	70% ACE	8.13 ± 0.20 ^Hd^	0.99 ± 0.03 ^Ic^	0.8 ± 0.22 ^Da^	0.32 ± 0.07 ^Kb^	2.8 ± 0.09 ^Gb^	20.04 ± 0.23 ^Aa^
	70% MeOH	4.32 ± 0.15 ^Ke^	0.04 ± 0.01 ^Md^	1.61 ± 0.09 ^Cb^	0.29 ± 0.07 ^Kd^	6.19 ± 0.01 ^Cc^	0.19 ± 0.01 ^Le^
	70% EtOH	5.01 ± 0.33 ^Je^	0.78 ± 0.01 ^Hc^	0.53 ± 0.07 ^Eb^	0.39 ± 0.05 ^JKe^	4.8 ± 0.07D ^Ec^	2.91 ± 0.26 ^Hc^
	EA	0.09 ± 0.01 ^Nd^	6.35 ± 0.01 ^Bc^	-	0.9 ± 0.01 ^FGe^	-	-

All values are expressed as the mean ± SD and performed in triplicates. Different letters (^a,b,c,d,e^) within the same column are significantly different (*p* < 0.05) samples within the solvent whereas letters (^A–Q^) within the same column are significantly different (*p* < 0.05) samples within the species. Six species of seaweed are reported based on dry weight. CE (catechin equivalents), QE (quercetin equivalents), GAE (gallic acid equivalents), PGE (phloroglucinol equivalents). TFC (total flavonoids content), TPC (total phenolic content), TCT (total tannins content), DMBA (2,4-dimethoxybenzaldehyde assay), PBA (Prussian blue assay), FDA (Folin–Denis Assay). The abbreviation of solvents expressed are ACE (70% Acetone), MeOH (70% Methanol), EtOH (70% Ethanol), EA (Absolute Ethyl Acetate).

**Table 2:** Estimation of freeze-dried antioxidant potential of seaweed species extracted by the conventional and non-conventional method of ultrasonication.

Samples	Solvents	DPPH (mg TE/g)	FRAP (mg TE/g)	ABTS (mg TE/g)	FICA (mg EDTA/g)	·OH-RSA (mg TE/g)	TAC (mg TE/g)	RPA (mg TE/g)
Ultrasonication								
*Cystophora* sp.	70% ACE	50.33 ± 0.07 ^Aa^	45.62 ± 0.13 ^Aa^	64.15 ± 0.20 ^Aa^	1.76 ± 0.01 ^BCd^	13.26 ± 0.22 ^Gd^	76.61 ± 0.30 ^Aa^	17.21 ± 0.03 ^Gc^
	70% MeOH	43.6 ± 0.10 ^Ba^	26.08 ± 0.22 ^Ba^	58.65 ± 0.18 ^Ba^	0.96 ± 0.01 ^Fd^	62.67 ± 0.44 ^Bb^	29.23 ± 1.04 ^Ic^	16.11 ± 0.08 ^Hc^
	70% EtOH	7.26 ± 0.03 ^Hb^	25.00 ± 0.22 ^Ca^	44.24 ± 0.18 ^Db^	1.04 ± 0.01 ^Fa^	-	33.30 ± 1.20 ^Gb^	29.27 ± 0.14 ^Aa^
	EA	1.42 ± 0.8 ^Nb^	0.35 ± 0.01 ^Nc^	6.58 ± 0.06 ^Pb^	1.45 ± 0.9 ^BCb^	9.06 ± 0.55 ^I^	20.24 ± 0.6 ^Kc^	1.95± 0.35 ^Pb^
*Phyllospora comosa*	70% ACE	37.52 ± 0.07 ^Dc^	17.01 ± 0.12 ^Gc^	22.99 ± 0.85 ^Kb^	4.14 ± 0.11 ^Aa^	53.38 ± 1.10 ^Cb^	48.71 ± 0.76 ^Cc^	25.76 ± 0.09 ^Cb^
	70% MeOH	4.76 ± 0.03 ^Le^	7.80 ± 0.23 ^Ic^	33.22 ± 0.33 ^Fc^	1.91 ± 0.02 ^Ba^	65.42 ± 0.96 ^Ba^	33.52 ± 0.60 ^Ga^	20.46 ± 0.18 ^Da^
	70% EtOH	4.69 ± 0.01 ^Kd^	11.02 ± 0.19 ^Hd^	32.21 ± 0.11 ^Ge^	1.59 ± 0.01 ^B–Db^	15.38 ± 0.17 ^Fa^	40.94 ± 0.92 ^Da^	7.24 ± 0.04 ^Me^
	EA	2.1 ± 0.03 ^Ma^	0.53 ± 0.04 ^Nd^	9.03 ± 0.03 ^Oa^	1.02 ± 0.04 ^Fb^	33.23 ± 0.15 ^H^	-	2.90 ± 0.04 ^Oa^
*Sargassum* sp.	70% ACE	38.79 ± 0.06 ^Cb^	36.73 ± 0.27 ^Db^	45.16 ± 0.40 ^Cd^	1.45 ± 0.01 ^C–Ed^	16.73 ± 0.36 ^Ec^	56.01 ± 0.35 ^Bb^	28.47 ± 0.14 ^Ba^
	70% MeOH	7.89 ± 0.06 ^Ga^	35.60 ± 0.21 ^Eb^	30.13 ± 0.07 ^Hd^	1.11 ± 0.01 ^Fc^	3.66 ± 0.01 ^Fd^	32.58 ± 0.63 ^GHb^	15.53 ± 0.04 ^Id^
	70% EtOH	7.84 ± 0.03 ^Ga^	27.63 ± 0.16 ^Fb^	30.22 ± 0.75 ^Ha^	1.01 ± 0.01 ^Ga^	1.33 ± 0.05 ^Kd^	31.63 ± 0.46 ^Hc^	18.52 ± 0.19 ^Fb^
	EA	0.62 ± 0.01 ^Oc^	0.09 ± 0.01 ^Mb^	4.86 ± 0.05 ^Qd^	0.52 ± 0.02 ^Hc^	-	40.30 ± 1.26 ^Ea^	-
*Ecklonia radiata*	70% ACE	34.09 ± 0.05 ^Ed^	24.10 ± 0.12 ^Db^	24.92 ± 0.45 ^Jd^	1.87 ± 0.01 ^Bb^	134.49 ± 5.25 ^Aa^	37.82 ± 1.5 ^Fd^	11.90 ± 0.04 ^Jd^
	70% MeOH	5.01 ± 0.01 ^Ld^	20.80 ± 0.09 ^Eb^	40.91 ± 0.99 ^Eb^	0.53 ± 0.01 ^He^	11.94 ± 0.01 ^Cb^	15.35 ± 0.76 ^Ld^	19.10 ± 0.08 ^Eb^
	70% EtOH	5.51 ± 0.02 ^Ic^	19.20 ± 0.16 ^Fb^	21.09 ± 0.69 ^Lc^	1.87 ± 0.01 ^D–Fa^	3.23 ± 0.06 ^Jc^	26.43 ± 1.5 ^Jd^	8.61 ± 0.08 ^Ld^
	EA	-	0.84 ± 0.01 ^Mb^	6.75 ± 0.10 ^PQc^	0.66 ± 0.01 ^Ba^	-	33.79 ± 0.42 ^Hb^	1.88 ± 0.02 ^Qc^
*Durvillaea* sp.	70% ACE	22.49 ± 0.04 ^Fe^	3.58 ± 0.08 ^Ld^	28.42 ± 0.07 ^Ie^	1.06 ± 0.01 ^Fe^	13.12 ± 0.16 ^Gd^	18.50 ± 0.08 ^Ke^	4.68 ± 0.16 ^Ne^
	70% MeOH	5.24 ± 0.01 ^Jc^	6.39 ± 0.12 ^Jd^	17.67 ± 0.09 ^Ne^	1.16 ± 0.01 ^EFb^	18.22 ± 0.02 ^Dc^	7.01 ± 0.15 ^Ne^	11.99 ± 0.04 ^Je^
	70% EtOH	5.47 ± 0.01 ^Ic^	11.39 ± 0.08 ^Hc^	18.36 ± 0.40 ^Mc^	1.01 ± 0.01 ^Fa^	14.27 ± 0.09 ^Fb^	9.36 ± 0.15 ^Me^	11.65 ± 0.06 ^Kc^
	EA	2.01 ± 0.01 ^Ma^	0.25 ± 0.01 ^Ka^	6.40 ± 0.03 ^Qd^	1.92 ± 0.01 ^Ba^	-	5.86 ± 0.05 ^Od^	2.00 ± 0.03 ^Pb^
Conventional Extraction								
*Cystophora* sp.	70% ACE	46.73 ± 0.12 ^Aa^	32.54 ± 0.29 ^Ca^	42.50 ± 0.22 ^Aa^	0.24 ± 0.01	0.70 ± 0.73 ^Jc^	77.63 ± 0.17 ^Aa^	7.92 ± 0.03 ^Fd^
	70% MeOH	42.95 ± 0.12 ^Ca^	32.88 ± 0.12 ^Ba^	41.10 ± 0.15 ^Ba^	0.10 ± 0.01 ^Nc^	2.20 ± 0.74 ^Hc^	46.00 ± 0.54 ^Ca^	15.20 ± 0.14 ^Ba^
	70% EtOH	46.49 ± 0.08 ^Ba^	33.48 ± 0.23 ^Aa^	42.34 ± 0.32 ^Aa^	0.37 ± 0.01 ^Ke^	3.72 ± 0.05 ^Gc^	73.79 ± 0.30 ^Ba^	22.01 ± 0.10 ^Aa^
	EA	4.40 ± 0.06 ^Jb^	0.25 ± 0.01 ^Opb^	3.41 ± 0.04 ^Lb^	0.93 ± 0.01 ^Ee^	-	17.59 ± 0.35 ^Ha^	2.27 ± 0.09 ^La^
*Sargassum* sp.	70% ACE	32.94 ± 0.17 ^Ec^	30.35 ± 0.17 ^Db^	23.43 ± 0.27 ^Cb^	0.36 ± 0.02 ^Kc^	23.37 ± 0.74 ^Ca^	34.75 ± 0.52 ^Cb^	10.55 ± 0.08 ^Da^
	70% MeOH	5.97 ± 0.08 ^Hc^	17.83 ± 0.23 ^Fb^	23.34 ± 0.48 ^Cb^	0.46 ± 0.01 ^Jc^	3.60 ± 0.29 ^Gd^	8.85 ± 0.17 ^Dd^	7.84 ± 0.05 ^Hd^
	70% EtOH	5.87 ± 0.07 ^Hc^	16.96 ± 0.32 ^Gb^	21.53 ± 0.48 ^Eb^	0.48 ± 0.03 ^Jc^	0.39 ± 0.35 ^Jd^	14.77 ± 0.18 ^Ld^	8.91 ± 0.08 ^Fc^
	EA	3.44 ± 0.19 ^Kc^	0.40 ± 0.01 ^Oa^	2.88 ± 0.03 ^Mc^	0.35 ± 0.02 ^Kd^	-	16.64 ± 1.30 ^Ib^	0.43 ± 0.01 ^Mb^
*Phyllospora comosa*	70% ACE	35.09 ± 0.09 ^Db^	12.69 ± 0.23 ^Hd^	20.24 ± 0.87 ^Fd^	0.98 ± 0.01 ^Da^	5.80 ± 0.01 ^Eb^	18.01 ± 0.63 ^Jd^	8.27 ± 0.04 ^Gc^
	70% MeOH	4.99 ± 0.01 ^Id^	6.56 ± 0.12 ^Ld^	12.56 ± 0.10 ^Ijd^	0.78 ± 0.01 ^Fa^	26.63 ± 0.12 ^Aa^	13.71 ± 0.31 ^Ic^	9.94 ± 0.05 ^Eb^
	70% EtOH	5.12 ± 0.01 ^Hd^	9.18 ± 0.29 ^Kd^	12.66 ± 0.07 ^Ijd^	1.19 ± 0.01 ^Ba^	4.51 ± 0.13 ^Fb^	21.69 ± 0.09 ^Fa^	11.90 ± 0.12 ^Cb^
	EA	0.69 ± 0.01 ^Me^	0.17 ± 0.01 ^Opb^	2.85 ± 0.05 ^Ka^	1.02 ± 0.01 ^Cb^	-	-	0.44 ± 0.02 ^Mb^
*Ecklonia radiata*	70% ACE	41.96 ± 0.07 ^Fd^	20.88 ± 0.12 ^Ec^	25.35 ± 0.62 ^Dc^	0.66 ± 0.01 ^Ib^	2.17 ± 0.05 ^H^	27.91 ± 0.17 ^Fc^	8.71 ± 0.07 ^Ge^
	70% MeOH	31.59 ± 0.08 ^Fb^	12.32 ± 0.12 ^Ic^	19.62 ± 0.40 ^Hc^	0.37± 0.01 ^Ld^	6.75 ± 0.15 ^Dc^	28.09 ± 0.15 ^Eb^	10.17 ± 0.07 ^Fc^
	70% EtOH	29.41 ± 0.12 ^Gb^	14.06 ± 0.19 ^Hc^	13.75 ± 0.23 ^Ijc^	0.66 ± 0.01 ^Jd^	1.11 ± 0.65 ^Ie^	28.24 ± 0.23 ^Gc^	11.43 ± 0.04 ^Fc^
	EA	2.51 ± 0.04 ^Ld^	0.02 ± 0.01 ^Pc^	-	4.56 ± 0.07 ^Aa^	-	-	0.14 ± 0.01 ^Nd^
*Durvillaea* sp.	70% ACE	30.54 ± 0.06 ^Fe^	10.25 ± 0.08 ^Je^	12.86 ± 0.07 ^Ie^	0.27 ± 0.01 ^Md^	4.56 ± 0.14 ^Ec^	13.31 ± 0.09 ^Ke^	9.84 ± 0.04 ^Ec^
	70% MeOH	4.73 ± 0.02 ^Je^	6.20 ± 0.20 ^Me^	12.60 ± 0.07 ^Ijd^	0.56 ± 0.02 ^Hb^	16.80 ± 0.15 ^Bb^	4.09 ± 0.04 ^Ne^	5.12 ± 0.04 ^Je^
	70% EtOH	4.77 ± 0.02 ^Je^	5.83 ± 0.16 ^Ne^	12.29 ± 0.04 ^Je^	0.66 ± 0.01 ^Gb^	6.50 ± 0.23 ^Da^	5.34 ± 0.14 ^Me^	4.51 ± 0.03 ^Kd^
	EA	4.82 ± 0.03 ^Ja^	0.03 ± 0.01 ^Pc^	1.87 ± 0.04 ^Ne^	0.93 ± 0.01 ^Ec^	-	8.01 ± 0.53 ^Lc^	-

All values are expressed as the mean ± SD and performed in triplicates. Different letters (^a,b,c,d,e,j^) within the same column are significantly different (*p* < 0.05) samples within the solvent whereas letters (^A–Q^) within the same column are significantly different (*p* < 0.05) samples within the species. Six species of seaweed are reported based on dry weight. TE (Trolox equivalents), EDTA (ethylenediaminetetraacetic acid), FRAP (ferric reducing antioxidant power), DPPH (2,2′-diphenyl-1-picrylhydrazyl), TAC (total antioxidant capacity), ABTS (2,2′-azino-bis-3-ethylbenzothiazoline-6-sulfonic acid), RPA (reducing power assay), ·OH-RSA (hydroxyl radical scavenging activity), FICA (ferrous ion chelating activity). The abbreviation of solvents expressed are ACE (Acetone), MeOH (Methanol), EtOH (Ethanol), EA (Ethyl Acetate).

**Table 3:** Pearson’s correlation coefficients (*r*) of phenolic contents and the antioxidant capacity for ultrasonication and conventional methodologies.

Variables	TPC	TFC	TCT	DMBA	PBA	FDA	DPPH	FRAP	ABTS	FICA	OH-RSA	TAC
Ultrasonication												
**TFC**	−0.178											
**TCT**	0.222	−0.155										
**DMBA**	−0.469	0.491 ^b^	−0.414									
**PBA**	0.649 ^a^	−0.238	0.394	−0.663								
**FDA**	0.818 ^a^	−0.297	0.422	−0.421	0.432							
**DPPH**	0.505 ^b^	−0.349	−0.170	−0.236	0.178	0.498 ^b^						
**FRAP**	0.731 ^a^	−0.365	−0.145	−0.503	0.403	0.600 ^a^	0.657 ^a^					
**ABTS**	0.837 ^a^	−0.245	0.110	−0.571	0.510 ^b^	0.734 ^a^	0.690 ^a^	0.815 ^a^				
**FICA**	0.202	−0.067	0.118	0.150	0.114	0.279	0.406	0.111	0.016			
**OH-RSA**	0.165	−0.270	0.254	−0.164	0.311	0.024	0.474 ^b^	0.114	0.161	0.375		
**TAC**	0.448 ^b^	−0.442	−0.111	−0.193	0.110	0.571 ^a^	0.646 ^a^	0.650 ^a^	0.562 ^a^	0.326	0.120	
**RPA**	0.819 ^a^	−0.294	0.286	−0.458	0.682 ^a^	0.771 ^a^	0.480 ^b^	0.712 ^a^	0.699 ^a^	0.313	0.182	0.449 ^b^
Conventional												
**TFC**	−0.434											
**TCT**	−0.292	−0.205										
**DMBA**	−0.668	0.511 ^b^	0.488									
**PBA**	0.457 ^b^	−0.384	−0.196	−0.513								
**FDA**	0.478 ^b^	−0.195	−0.133	−0.393	0.262							
**DPPH**	0.799 ^a^	−0.262	−0.186	−0.445	0.167	0.561 ^b^						
**FRAP**	0.907 ^a^	−0.304	−0.217	−0.538	0.478 ^a^	0.447 ^b^	0.824 ^a^					
**ABTS**	0.921 ^b^	−0.280	−0.257	−0.567	0.457 ^a^	0.451 ^b^	0.809 ^a^	0.958 ^a^				
**FICA**	−0.460	−0.122	0.926 ^a^	0.537 ^b^	−0.298	−0.321	−0.376	−0.443	−0.480			
**OH-RSA**	0.247	−0.444	−0.153	−0.390	0.190	−0.40	−0.021	0.112	0.047	−0.168		
**TAC**	0.761 ^a^	0.055 ^b^	−0.220	−0.317	0.154	0.403	0.809 ^a^	0.837 ^a^	0.851 ^a^	−0.393	−0.063	
**RPA**	0.830 ^a^	−0.449	−0.273	−0.625	0.628 ^a^	0.541 ^b^	0.688 ^a^	0.785 ^a^	0.787 ^a^	−0.423	0.219	0.685 ^a^

^a^ Significant correlation with *p* ≤ 0.01; ^b^ Significant correlation with *p* ≤ 0.05.

**Table 4:** Characterization of phenolic compounds in ultrasound extracted seaweed samples by LC-ESI-QTOF-MS/MS.

No.	Proposed Compounds	Molecular Formula	RT (min)	Ionization (ESI^+^/ESI^−^)	Molecular Weight	Theoretical (*m*/*z*)	Observed (*m*/*z*)	Error (ppm)	MS^2^ Product Ions	Seaweed Samples
Phenolic acid										
Hydroxybenzoic acids										
1	4-Hydroxybenzaldehyde	C_7_H_6_O_2_	7.188	[M – H]^−^	122.0376	121.0303	121.0303	0.2	77	Dabu
2	Protocatechuic acid 4-*O*-glucoside	C_13_H_16_O_9_	14.994	[M – H]^−^	316.0786	315.0713	315.0702	−3.5	153	* Embu, Dmbu, Eeu
3	2,3-Dihydroxybenzoic acid	C_7_H_6_O_4_	25.802	[M – H]^−^	154.0262	153.0189	153.0190	0.7	109	Eethbu
4	Gallic acid 4-*O*-glucoside	C_13_H_16_O_10_	30.462	** [M – H]^−^	332.0768	331.0695	331.0691	−1.2	169, 125	* Amu, Dmu, Aeu, Aau, Cabu, Cebu, Dmbu
5	Gallic acid	C_7_H_6_O_5_	31.309	[M – H]^−^	170.0225	169.0152	169.0154	1.2	125	* Aabu, Aebu, Ambu, Babu, Bebu, Bethbu, Bmbu, Dethbu, Eabu, Eethbu, Eeu, Eau
6	2-Hydroxybenzoic acid	C_7_H_6_O_3_	32.019	[M – H]^−^	138.0310	137.0237	137.0238	0.7	93	* Dabu, Dmbu
Hydroxycinnamic acids										
7	Feruloyl tartaric acid	C_14_H_14_O_9_	4.933	[M – H]^−^	326.0652	325.0579	325.0575	−1.2	193, 149	* Eau, Cau
8	*m*-Coumaric acid	C_9_H_8_O_3_	5.228	[M – H]^−^	164.0487	163.0414	163.0412	−1.2	119	* Dabu, Cmbu, Debu, Dmbu, Eabu
9	Caffeoyl tartaric acid	C_13_H_12_O_9_	5.426	[M – H]^−^	312.0504	311.0431	311.0438	2.3	161	* Emu, Dmu, Amu
10	Ferulic acid	C_10_H_10_O_4_	5.539	[M – H]^−^	194.0585	193.0512	193.0516	2.1	178, 149, 134	Eau
11	Isoferulic acid 3-sulfate	C_10_H_10_O_7_S	5.608	[M – H]^−^	274.0129	273.0056	273.0054	−0.7	193, 178	* Aabu, Aethbu, Ambu, Bmbu, Cabu, Dabu, Amu
12	Caffeic acid 3-*O*-glucuronide	C_15_H_16_O_10_	6.388	** [M – H]^−^	356.0743	355.0670	355.0673	0.8	179	* Cebu, Ambu, Embu, Cethbu, Emu, Amu
13	Hydroxycaffeic acid	C_9_H_8_O_5_	7.205	[M – H]^−^	196.0368	195.0295	195.0299	2.1	151	Cebu
14	Cinnamic acid	C_9_H_8_O_2_	7.246	[M – H]^−^	148.0537	147.0464	147.0465	0.7	103	* Cebu, Aethbu, Bmbu, Cabu, Cmbu, Dabu, Eabu
15	Ferulic acid 4-*O*-glucoside	C_16_H_20_O_9_	13.750	[M – H]^−^	356.1106	355.1033	355.1025	−2.3	193, 178, 149, 134	* Aabu, Babu
16	Chlorogenic acid	C_16_H_18_O_9_	13.985	[M – H]^−^	354.0929	353.0856	353.0855	−0.3	253, 190, 144	* Dabu, Babu, Cabu, Cebu, Eabu, Eau, Aeu
17	1-Sinapoyl−2-feruloylgentiobiose	C_33_H_40_O_18_	14.898	** [M – H]^−^	724.2205	723.2132	723.2136	0.6	529, 499	* Bmbu, Cabu, Cethbu, Cmbu, Dmbu, Eebu, Aeu
18	Sinapic acid	C_11_H_12_O_5_	16.158	** [M – H]^−^	224.0666	223.0593	223.0595	0.9	205, 163	* Aabu, Cethbu, Dabu, Debu, Dmbu
19	*p*-Coumaroyl tartaric acid	C_13_H_12_O_8_	16.214	[M – H]^−^	296.0555	295.0482	295.0486	1.4	115	* Debu, Dmbu, Embu
20	*p*-Coumaroyl malic acid	C_13_H_12_O_7_	16.596	** [M – H]^−^	280.0596	279.0523	279.0512	−3.9	163, 119	* Dmbu, Cabu, Cebu, Cethbu, Dabu, Dmbu, Emu, Aeu
21	1,5-Dicaffeoylquinic acid	C_25_H_24_O_12_	17.017	[M – H]^−^	516.1233	515.1160	515.1172	2.3	353, 335, 191, 179	* Dabu, Eabu, Eau
22	Ferulic acid 4-*O*-glucuronide	C_16_H_18_O_10_	17.199	** [M – H]^−^	370.0895	369.0822	369.0836	3.8	193	* Eau, Bmu, Dabu, Debu, Embu
23	*p*-Coumaric acid 4-*O*-glucoside	C_15_H_18_O_8_	17.367	[M – H]^−^	326.0970	325.0897	325.0891	−1.8	163	* Eabu, Dabu
24	Rosmarinic acid	C_18_H_16_O_8_	18.249	[M – H]^−^	360.0850	359.0777	359.0775	−0.6	179	* Dmbu, Emu
25	Caffeoyl glucose	C_15_H_18_O_9_	18.549	[M – H]^−^	342.0951	341.0878	341.0890	3.5	179, 161	* Dmbu, Dabu, Eabu
26	3-*p*-Coumaroylquinic acid	C_16_H_18_O_8_	19.117	[M – H]^−^	338.0998	337.0925	337.0926	0.3	265, 173, 162	* Dabu, Cabu, Eabu, Eebu, Eau
27	*p*-Coumaric acid 4-*O*-glucoside	C_15_H_18_O_7_	21.240	[M – H]^−^	310.1024	309.0951	309.0951	0.1	163	Debu
28	5-5′-Dehydrodiferulic acid	C_20_H_18_O_8_	21.657	** [M – H]^+^	386.0985	385.0912	385.0913	0.3	369	* Eabu, Embu, Cebu
29	3-Sinapoylquinic acid	C_18_H_22_O_10_	23.306	[M – H]^−^	398.1177	397.1104	397.1109	1.3	233, 179	Eeu
30	1,2,2′-Triferuloylgentiobiose	C_42_H_46_O_20_	31.165	[M – H]^−^	870.2584	869.2511	869.2481	−3.5	693, 517	* Deu, Bau
Hydroxyphenyl acetic acids										
31	3,4-Dihydroxyphenylacetic acid	C_8_H_8_O_4_	6.661	[M – H]^−^	168.0432	167.0359	167.0350	−5.4	149, 123	* Deu
32	2-Hydroxy-2-phenylacetic acid	C_8_H_8_O_3_	7.182	[M – H]^−^	152.0475	151.0402	151.0403	0.7	136, 92	* Cebu, Aeu, Aau
Hydroxyphenylpropanoic acids										
33	Dihydroferulic acid 4-*O*-glucuronide	C_16_H_20_O_10_	3.119	** [M – H]^−^	372.1090	371.1017	371.1019	0.5	195	* Babu, Bebu, Bmbu, Dabu, Eabu, Embu, Cethbu
34	Dihydrocaffeic acid 3-*O*-glucuronide	C_15_H_18_O_10_	7.535	[M – H]^−^	358.0913	357.0840	357.0837	−0.8	181	* Bebu, Bmbu, Debu, Eabu, Eebu, Embu, Aabu
35	Dihydroferulic acid 4-sulfate	C_10_H_12_O_7_S	16.763	[M – H]^−^	276.0290	275.0217	275.0212	−1.8	195, 151, 177	Eebu
Flavonoids										
Flavanols										
36	Theaflavin	C_29_H_24_O_12_	3.082	[M – H]^−^	564.1254	563.1181	563.1198	3.0	545	* Bebu, Dabu, Eeu
37	(+)-Gallocatechin 3-*O*-gallate	C_22_H_18_O_11_	5.144	[M – H]^−^	458.0818	457.0745	457.0744	−0.2	305, 169	* Cebu, Debu
38	Procyanidin dimer B1	C_30_H_26_O_12_	17.032	[M – H]^−^	578.1385	577.1312	577.1292	−3.5	451	Babu
39	3′-*O*-Methylcatechin	C_16_H_16_O_6_	17.951	** [M – H]^−^	304.0959	303.0886	303.0894	2.6	271, 163	Babu
40	(+)-Catechin 3-*O*-gallate	C_22_H_18_O_10_	20.261	[M – H]^−^	442.0879	441.0806	441.0811	1.1	289, 169, 125	* Embu, Eeu
41	(+)-Catechin	C_15_H_14_O_6_	20.437	** [M – H]^−^	290.0786	289.0713	289.0716	1.0	245, 205, 179	* Eeu, Dabu
42	Theaflavin 3,3′-*O*-digallate	C_43_H_32_O_20_	24.533	[M – H]^−^	868.1448	867.1375	867.1373	−0.2	715, 563, 545	* Bmu, Beu, Bau
43	(−)-Epigallocatechin	C_15_H_14_O_7_	30.714	** [M – H]^−^	306.0737	305.0664	305.0670	2.0	261, 219	* Aau, Dau, Deu, Debu, Eabu, Embu
Flavones										
44	Apigenin 6-C-glucoside	C_21_H_20_O_10_	16.981	[M – H]^−^	432.1077	431.1004	431.1015	2.6	413, 341, 311	* Dabu, Dmbu
45	Isorhamnetin	C_16_H_12_O_7_	19.215	[M – H]^−^	316.0575	315.0502	315.0510	2.5	300, 271	Dmu
46	Apigenin 7-*O*-glucuronide	C_21_H_18_O_11_	22.686	** [M – H]^−^	446.0875	445.0802	445.0802	0.2	271, 253	* Aau, Amu
47	3-Methoxysinensetin	C_21_H_22_O_8_	30.813	[M – H]^−^	402.1301	401.1228	401.1234	1.5	388, 373, 355, 327	Eau
Flavanones										
48	Hesperetin 3′,7-*O*-diglucuronide	C_28_H_30_O_18_	4.806	[M – H]^−^	654.1424	653.1351	653.1356	0.8	477, 301, 286, 242	* Babu, Cmbu, Debu, Eabu
49	Narirutin	C_27_H_32_O_14_	5.264	[M – H]^−^	580.1827	579.1754	579.1756	0.3	271	* Cmbu, Cabu, Cebu
50	Hesperetin 3′-sulfate	C_16_H_14_O_9_S	7.389	[M – H]^−^	382.0354	381.0281	381.0277	−1.0	301, 286, 257, 242	* Aethbu, Ambu, Babu, Cmbu, Dmbu, Eabu
51	Hesperetin 3′-*O*-glucuronide	C_22_H_22_O_12_	13.741	** [M – H]^−^	478.1130	477.1057	477.1062	1.0	301, 175, 113, 85	* Eau, Emu
52	Naringin 4′-*O*-glucoside	C_33_H_42_O_19_	32.747	[M – H]^−^	742.2295	741.2222	741.2232	1.3	433, 271	Cethbu
Flavonols										
53	Kaempferol 3-*O*-glucosyl-rhamnosyl-galactoside	C_33_H_40_O_20_	4.719	[M – H]^−^	756.2135	755.2062	755.2075	1.7	285	Ceu
54	Quercetin 3′-*O*-glucuronide	C_21_H_18_O_13_	5.185	[M – H]^−^	478.0758	477.0685	477.0679	−1.3	301	* Cabu, Bmu, Beu, Bethu, Bau
55	Patuletin 3-*O*-glucosyl-(1->6)-[apiosyl(1->2)]-glucoside	C_33_H_40_O_22_	5.616	[M – H]^−^	788.1982	787.1909	787.1919	1.3	625, 463, 301, 271	* Ceu, Cau
56	Myricetin 3-*O*-arabinoside	C_20_H_18_O_12_	7.182	[M – H]^−^	450.0821	449.0748	449.0750	0.4	317	* Cebu, Emu, Cau
57	Quercetin 3-*O*-glucosyl-xyloside	C_26_H_28_O_16_	15.852	[M – H]^−^	596.1347	595.1274	595.1280	1.0	265, 138, 116 *	Eabu
58	Isorhamnetin 3-*O*-glucuronide	C_22_H_20_O_13_	15.938	[M – H]^−^	492.0897	491.0824	491.0821	−0.6	315, 300, 272, 255	Aabu`
59	Myricetin 3-*O*-galactoside	C_21_H_20_O_13_	16.921	** [M – H]^−^	480.0911	479.0838	479.0848	2.1	317	* Eeu, Deu, Beu, Bau
60	Myricetin 3-*O*-rhamnoside	C_21_H_20_O_12_	17.690	[M – H]^−^	464.0937	463.0864	463.0879	3.2	317	* Eeu, Beu
61	Quercetin 3-*O*-arabinoside	C_20_H_18_O_11_	20.258	** [M – H]^−^	434.0850	433.0777	433.0796	4.4	301	* Eeu, Dmu, Dmbu
62	6-Hydroxyluteolin 7-rhamnoside	C_21_H_20_O_11_	20.989	[M – H]^−^	448.0991	447.0918	447.0904	−3.1	301	* Eeu, Eau
63	Quercetin 3′-sulfate	C_15_H_10_O_10_S	31.909	[M – H]^−^	381.9980	380.9907	380.9905	−0.5	301	* Ceu, Cau, Beu, Bau, Aau
64	3-Methoxynobiletin	C_22_H_24_O_9_	34.064	[M + H]^+^	432.1461	433.1534	433.1534	0.1	403, 385, 373, 345	* Aethu
Dihydroflavonols										
65	Dihydromyricetin 3-*O*-rhamnoside	C_21_H_22_O_12_	15.652	** [M – H]^−^	466.1112	465.1039	465.1043	0.9	301	* Eau, Deu
66	Dihydroquercetin	C_15_H_12_O_7_	15.971	[M – H]^−^	304.0591	303.0518	303.0518	0.2	285, 275, 151	* Emu, Dabu
Dihydrochalcones										
67	3-Hydroxyphloretin 2′-*O*-glucoside	C_21_H_24_O_11_	4.593	[M – H]^−^	452.1354	451.1281	451.1280	−0.2	289, 273	Bau
Isoflavonoids										
68	6″-*O*-Acetylglycitin	C_24_H_24_O_11_	5.896	[M + H]^+^	488.1333	489.1406	489.1398	−1.6	285, 270	* Amu
69	2-Dehydro-*O*-desmethylangolensin	C_15_H_12_O_4_	5.898	[M – H]^−^	256.0751	255.0678	255.0679	0.4	135, 119	* Cmbu, Babu
70	2′-Hydroxyformononetin	C_16_H_12_O_5_	5.934	** [M – H]^−^	284.0687	283.0614	283.0619	1.8	270, 229	* Emu, Eeu, Dmu
71	Violanone	C_17_H_16_O_6_	5.937	[M – H]^−^	316.0932	315.0859	315.0850	−2.9	300, 285, 135	* Cmbu, Embu, Eeu
72	Sativanone	C_17_H_16_O_5_	16.707	** [M – H]^−^	300.0987	299.0914	299.0914	0.1	284, 269, 225	* Deu, Emu, Eau
73	Pseudobaptigenin	C_16_H_10_O_5_	19.160	** [M – H]^−^	282.0506	281.0433	281.0431	−0.7	263, 237	* Dau, Aau
74	Dalbergin	C_16_H_12_O_4_	21.505	[M – H]^−^	268.0717	267.0644	267.0656	4.5	252, 224, 180	* Deu, Ceu
75	6″-*O*-Malonyldaidzin	C_24_H_22_O_12_	22.473	[M + H]^+^	502.1085	503.1158	503.1149	−1.8	255	Bau
76	Genistein 4′,7-*O*-diglucuronide	C_27_H_26_O_17_	23.862	** [M – H]^−^	622.1174	621.1101	621.1122	3.4	269	Emu, Eeu, Dau, Cau, Bau, Aau
77	Glycitin	C_22_H_22_O_10_	30.888	[M – H]^−^	446.1206	445.1133	445.1124	−2.0	285	* Cethbu, Eabu
Other polyphenols										
Hydroxycoumarins										
78	Esculetin	C_9_H_6_O_4_	24.158	[M – H]^−^	178.0280	177.0207	177.0207	0.1	149, 133, 89	Bau
79	Scopoletin	C_10_H_8_O_4_	31.117	[M – H]^−^	192.0419	191.0346	191.0347	0.5	176	* Bethbu, Eeu, Eau, Bmu, Beu, Bau
Hydroxybenzoketones										
80	2-Hydroxy-4-methoxyacetophenone 5-sulfate	C_9_H_10_O_7_S	24.081	** [M – H]^−^	262.0155	261.0082	261.0081	−0.4	181, 97	* Aeu, Aau
Phenolic terpenes										
81	Carnosic acid	C_20_H_28_O_4_	32.549	** [M – H]^−^	332.2004	331.1931	331.1933	0.6	287, 269	* Dethbu, Cethbu, Emu, Eau, Dmu, Deu, Dau, Ceu, Cethu, Cau, Bmu, Beu, Bethu, Bau, Amu, Aau
Tyrosols										
82	3,4-DHPEA-AC	C_10_H_12_O_4_	24.521	[M – H]^−^	196.0736	195.0663	195.0662	−0.5	135	Amu
83	3,4-DHPEA-EDA	C_17_H_20_O_6_	29.370	[M – H]^−^	320.1270	319.1197	319.1192	−1.6	275, 195	* Aabu, Bmbu
Alkylmethoxyphenols										
84	Equol	C_15_H_14_O_3_	16.915	[M + H]^+^	242.0943	243.1016	243.1019	1.2	255, 211, 197	* Emu, Eau, Dau
Other polyphenols										
85	Salvianolic acid C	C_26_H_20_O_10_	16.686	[M – H]^−^	492.1026	491.0953	491.0976	4.7	311, 267, 249	Embu
86	Arbutin	C_12_H_16_O_7_	19.621	[M – H]^−^	272.0901	271.0828	271.0827	−0.4	109	* Ceu, Cau
Lignans										
87	Schisandrol B	C_23_H_28_O_7_	3.062	** [M – H]^−^	416.1824	415.1751	415.1749	−0.5	224, 193, 165	Aebu
88	7-Hydroxymatairesinol	C_20_H_22_O_7_	3.296	** [M – H]^−^	374.1381	373.1308	373.1311	0.8	343, 313, 298, 285	* Aebu, Eebu
89	Todolactol A	C_20_H_24_O_7_	16.242	[M – H]^−^	376.1546	375.1473	375.1469	−1.1	313, 137	* Ambu, Dmbu
90	Sesamin	C_20_H_18_O_6_	24.500	** [M – H]^−^	354.1138	353.1065	353.1068	0.8	338, 163	* Deu, Aabu, Babu
91	Arctigenin	C_21_H_24_O_6_	26.476	[M – H]^−^	372.1565	371.1492	371.1494	0.5	356, 312, 295	* Embu, Eebu, Dmu, Dau, Cau, Aeu, Aau
92	Secoisolariciresinol-sesquilignan	C_30_H_38_O_10_	31.907	[M – H]^−^	558.2434	557.2361	557.2387	4.7	539, 521, 509, 361	Amu
Stilbenes										
93	Resveratrol	C_14_H_12_O_3_	17.529	** [M – H]^−^	228.0787	227.0714	227.0717	1.3	212, 185, 157, 143	* Eau, Deu, Dabu
94	Resveratrol 5-*O*-glucoside	C_20_H_22_O_8_	33.742	[M – H]^−^	390.1283	389.1210	389.1214	1.0	227	Debu

* Compound was detected in more than one seaweed samples, data presented in this table are from asterisk sample. ** Compounds were detected in both negative [M – H]^−^ and positive [M + H]^+^ mode of ionization while only single mode data was presented. Seaweed samples were mentioned in abbreviations.

**Table 5:** Characterization of phenolic compounds in conventional extracted seaweed samples by LC-ESI-QTOF-MS/MS.

No.	Proposed Compounds	Molecular Formula	RT (min)	Ionization (ESI^+^/ESI^−^)	Molecular Weight	Theoretical (*m*/*z*)	Observed (*m*/*z*)	Error (ppm)	MS2 Product Ions	Seaweed Samples
Phenolic acid										
Hydroxybenzoic acids										
1	Protocatechuic acid 4-*O*-glucoside	C_13_H_16_O_9_	3.062	[M – H]^−^	316.0818	315.0745	315.0751	1.9	153	* Cethbc, Cebc, Debc, Dmbc
2	4-Hydroxybenzoic acid 4-*O*-glucoside	C_13_H_16_O_8_	7.687	[M – H]^−^	300.0839	299.0766	299.0773	2.3	255, 137	Dabc
3	4-Hydroxybenzaldehyde	C_7_H_6_O_2_	25.146	[M – H]^−^	122.0366	121.0293	121.0294	0.8	77	* Bethbc, Cmbc, Dabc, Dmbc
4	2,3-Dihydroxybenzoic acid	C_7_H_6_O_4_	25.793	[M – H]^−^	154.0259	153.0186	153.0185	−0.7	109	Bethbc
5	2-Hydroxybenzoic acid	C_7_H_6_O_3_	29.923	[M – H]^−^	138.0307	137.0234	137.0234	0.1	93	Bethbc
6	Gallic acid	C_7_H_6_O_5_	31.949	[M – H]^−^	170.0209	169.0136	169.0140	2.4	125	* Aec, Aac, Bac, Bec, Bethc, Bmc, Cec, Cac, Cethc, Dec, Dethc, Dmc, Aabc, Debc
7	3-*O*-Methylgallic acid	C_8_H_8_O_5_	33.658	[M – H]^−^	184.0355	183.0282	183.0280	−1.1	170, 142	* Dec, Dethc, Dmc
Hydroxycinnamic acids										
8	Chlorogenic acid	C_16_H_18_O_9_	3.078	[M – H]^−^	354.0969	353.0896	353.0902	1.7	253, 190, 144	* Cabc, Eabc
9	1-Sinapoyl-2,2′-diferuloylgentiobiose	C_43_H_48_O_21_	3.119	[M – H]^−^	900.2677	899.2604	899.2574	−3.3	613, 201	* Aebc, Debc
10	3-Feruloylquinic acid	C_17_H_20_O_9_	4.687	[M – H]^−^	368.1081	367.1008	367.1012	1.1	298, 288, 192, 191	Cmc
11	Ferulic acid 4-*O*-glucoside	C_16_H_20_O_9_	4.821	[M – H]^−^	356.1100	355.1027	355.1031	1.1	193, 178, 149, 134	Eac
12	Feruloyl tartaric acid	C_14_H_14_O_9_	4.958	[M – H]^−^	326.0641	325.0568	325.0562	−1.8	193, 149	* Dac, Dethc, Eac
13	Caffeic acid	C_9_H_8_O_4_	5.116	[M – H]^−^	180.0431	179.0358	179.0359	0.6	143, 133	Emc
14	Cinnamic acid	C_9_H_8_O_2_	7.400	** [M – H]^−^	148.0523	147.0450	147.0451	0.7	103	* Cmbc, Ambc
15	Caffeoyl glucose	C_15_H_18_O_9_	7.457	[M – H]^−^	342.0922	341.0849	341.0837	−3.5	179, 161	Eebc
16	*p*-Coumaric acid 4-*O*-glucoside	C_15_H_18_O_8_	15.736	[M – H]^−^	326.0993	325.0920	325.0920	0.1	163	Bebc
17	3-*p*-Coumaroylquinic acid	C_16_H_18_O_8_	16.364	[M – H]^−^	338.0974	337.0901	337.0900	−0.3	265, 173, 162	Dabc
18	*p*-Coumaroyl tartaric acid	C_13_H_12_O_8_	16.591	[M – H]^−^	296.0522	295.0449	295.0437	−4.1	115	Dmbc
19	Hydroxycaffeic acid	C_9_H_8_O_5_	17.250	[M – H]^−^	196.0376	195.0303	195.0304	0.5	151	* Aabc, Bethbc, Dabc, Dethbc
20	Chicoric acid	C_22_H_18_O_12_	17.787	[M – H]^−^	474.0826	473.0753	473.0739	−3.0	293, 311	Eac
21	Rosmarinic acid	C_18_H_16_O_8_	18.986	[M – H]^−^	360.0823	359.0750	359.0753	0.8	179	* Dmbc, Eabc, emc
22	Cinnamoyl glucose	C_15_H_18_O_7_	20.687	[M – H]^−^	310.1035	309.0962	309.0955	−2.3	147, 131, 103	* Dabc, Eabc
23	Sinapic acid	C_11_H_12_O_5_	22.780	** [M – H]^−^	224.0674	223.0601	223.0598	−1.3	205, 163	* Cabc, Aabc, Ambc, Babc, Bebc, Bmbc, Cebc, Eabc, Eac
24	1,5-Dicaffeoylquinic acid	C_25_H_24_O_12_	23.930	[M – H]^−^	516.1262	515.1189	515.1213	4.7	353, 335, 191, 179	Eec
25	Caffeic acid 3-*O*-glucuronide	C_15_H_16_O_10_	24.346	** [M – H]^−^	356.0756	355.0683	355.0674	−2.5	179	* Bmc, Bac, Dmc, Ambc, Dethbc
26	1,2,2′-Triferuloylgentiobiose	C_42_H_46_O_20_	30.541	[M – H]^−^	870.2536	869.2463	869.2486	2.6	693, 517	Aac
27	*m*-Coumaric acid	C_9_H_8_O_3_	32.821	[M – H]^−^	164.0477	163.0404	163.0408	2.5	119	Eethc
28	Ferulic acid 4-*O*-glucuronide	C_16_H_18_O_10_	33.763	[M + H]^+^	370.0902	371.0975	371.0974	−0.3	193	* Cebc, Dethbc
29	*p*-Coumaroyl malic acid	C_13_H_12_O_7_	34.094	[M + H]^+^	280.0580	281.0653	281.0650	−1.1	163, 119	* Aebc, Ambc, Debc
Hydroxyphenylacetic acids										
30	2-Hydroxy-2-phenylacetic acid	C_8_H_8_O_3_	14.463	[M – H]^−^	152.0473	151.0400	151.0401	0.7	136, 92	* Cmbc, Aabc, Cabc, Dabc, Eabc, Eethc, Dmc, Eac
31	3,4-Dihydroxyphenylacetic acid	C_8_H_8_O_4_	15.889	[M – H]^−^	168.0426	167.0353	167.0354	0.6	149, 123	* Aabc, Eethc
Hydroxyphenylpropanoic acids										
32	Dihydroferulic acid 4-*O*-glucuronide	C_16_H_20_O_10_	3.064	** [M – H]^−^	372.1073	371.1000	371.1004	1.1	195	* Aabc, Aebc, Cabc, Cebc, Cmbc, Debc, Dmbc, Eabc, Aethbc, Dethbc
33	Dihydrocaffeic acid 3-*O*-glucuronide	C_15_H_18_O_10_	3.077	** [M – H]^−^	358.0905	357.0832	357.0833	0.3	181	* Aabc, Aebc, Eabc. Eebc, Aabc, Bac, Bec, Cmc
Flavonoids										
Flavanols										
34	(+)-Gallocatechin 3-*O*-gallate	C_22_H_18_O_11_	7.625	[M – H]^−^	458.0830	457.0757	457.0755	−0.4	305, 169	* Babc, Bebc, Cebc, Debc
35	Prodelphinidin dimer B3	C_30_H_26_O_14_	16.094	[M + H]^+^	610.1318	611.1391	611.1366	−4.1	469, 311, 291	Aebc
36	Theaflavin	C_29_H_24_O_12_	16.799	[M – H]^−^	564.1257	563.1184	563.1190	1.1	545	* Dac, Emc, Embc
37	Procyanidin dimer B 1	C_30_H_26_O_12_	18.209	** [M – H]^−^	578.1444	577.1371	577.1378	1.2	451	* Cabc, Aebc, Dethbc
38	(−)-Epigallocatechin	C_15_H_14_O_7_	21.155	[M – H]^−^	306.0764	305.0691	305.0697	2.0	261, 219	* Aabc, Eabc
39	(+)-Catechin 3-*O*-gallate	C_22_H_18_O_10_	24.152	[M – H]^−^	442.0876	441.0803	441.0816	2.9	289, 169, 125	Cac
40	Theaflavin 3,3′-*O*-digallate	C_43_H_32_O_20_	26.891	[M – H]^−^	868.1512	867.1439	867.1439	0.1	715, 563, 545	Bac
41	4″-*O*-Methylepigallocatechin 3-*O*-gallate	C_23_H_20_O_11_	28.257	[M – H]^−^	472.1005	471.0932	471.0923	−1.9	169, 319	* Cac, Bec, Debc
42	4′-*O*-Methyl-(−)-epigallocatechin 7-*O*-glucuronide	C_22_H_24_O_13_	34.052	** [M – H]^−^	496.1186	495.1113	495.1117	0.8	451, 313	* Bmc, Bethc, Dmc, Cac, Dmc, Emc, Embc
Flavones										
43	Apigenin 7-*O*-glucuronide	C_21_H_18_O_11_	20.616	** [M + H]^+^	446.0826	445.0753	445.0755	0.4	271, 253	* Cac, Dmc, Emc, Aac, Dabc
44	Apigenin 7-*O*-(6″-malonyl-apiosyl-glucoside)	C_29_H_30_O_17_	20.668	[M – H]^−^	650.1509	649.1436	649.1445	1.4	605	* Dmc, Aac
45	Apigenin 6-C-glucoside	C_21_H_20_O_10_	21.777	[M – H]^−^	432.1045	431.0972	431.0980	1.9	413, 341, 311	* Dethc, Eec
46	Apigenin 7-*O*-apiosyl-glucoside	C_26_H_28_O_14_	22.663	[M – H]^−^	564.1524	563.1451	563.1459	1.4	296	Eec
47	3-Methoxysinensetin	C_21_H_22_O_8_	30.907	[M – H]^−^	402.1278	401.1205	401.1202	−0.7	388, 373, 355, 327	Dec
48	Apigenin 6,8-di-C-glucoside	C_27_H_30_O_15_	32.223	[M – H]^−^	594.1592	593.1519	593.1509	−1.7	503, 473	* Bac, Aec, Bec, Bethc
Flavanones										
49	Hesperetin 3′-sulfate	C_16_H_14_O_9_S	7.732	[M – H]^−^	382.0358	381.0285	381.0283	−0.5	301, 286, 257, 242	* Babc, Bebc, Cebc, Dmbc, Embc
50	Hesperetin 3′-*O*-glucuronide	C_22_H_22_O_12_	16.771	[M – H]^−^	478.1134	477.1061	477.1055	−1.3	301, 175, 113, 85	* Emc, Dac, Dmc, Dabc, Eabc, Eebc
51	Hesperetin 3′,7-*O*-diglucuronide	C_28_H_30_O_18_	18.725	[M – H]^−^	654.1415	653.1342	653.1356	2.1	477, 301, 286, 242	Embc
52	Naringin 4′-*O*-glucoside	C_33_H_42_O_19_	30.498	[M – H]^−^	742.2314	741.2241	741.2258	2.3	433, 271	Bec
53	Xanthohumol	C_21_H_22_O_5_	31.272	[M – H]^−^	354.1486	353.1413	353.1425	3.4	338, 309	Aethc
Flavonols										
54	Kaempferol 3-*O*-glucosyl-rhamnosyl-galactoside	C_33_H_40_O_20_	4.718	[M – H]^−^	756.2092	755.2019	755.2012	−0.9	285	Cmc
55	Kaempferol 3,7-*O*-diglucoside	C_27_H_30_O_16_	16.377	[M – H]^−^	610.1542	609.1469	609.1474	0.8	447, 285	Amc
56	Isorhamnetin 3-*O*-glucuronide	C_22_H_20_O_13_	16.474	[M – H]^−^	492.0928	491.0855	491.0872	3.5	315, 300, 272, 255	* Dac, Dmc
57	Myricetin 3-*O*-rhamnoside	C_21_H_20_O_12_	17.258	[M – H]^−^	464.0946	463.0873	463.0867	−1.3	317	* Dmc, Bac, Bec, Dmc, Eec, Emc
58	Quercetin 3-*O*-arabinoside	C_20_H_18_O_11_	18.213	[M – H]^−^	434.0837	433.0764	433.0772	1.8	301	* Dac, Dmc
59	Myricetin 3-*O*-arabinoside	C_20_H_18_O_12_	18.941	[M – H]^−^	450.0825	449.0752	449.0741	−2.4	317	Dmc
60	6-Hydroxyluteolin 7-rhamnoside	C_21_H_20_O_11_	19.191	[M – H]^−^	448.1003	447.0930	447.0933	0.7	301	* Emc, Cac, Dac, Dethc, Eec, Emc, Cmbc, Eebc
61	Quercetin 3-*O*-(6”-malonyl-glucoside)	C_24_H_22_O_15_	23.143	[M – H]^−^	550.0977	549.0904	549.0886	−3.3	303	Bac
62	Myricetin 3-*O*-galactoside	C_21_H_20_O_13_	24.356	[M – H]^−^	480.0909	479.0836	479.0837	0.2	317	* Emc, Bac, Bethc, Dmc
63	Quercetin 3-*O*-glucosyl-xyloside	C_26_H_28_O_16_	25.144	[M – H]^−^	596.1402	595.1329	595.1313	−2.7	265, 138, 116	Bac
64	Quercetin 3′-*O*-glucuronide	C_21_H_18_O_13_	31.165	[M – H]^−^	478.0785	477.0712	477.0717	1.0	301	* Bec, Bac
65	Quercetin 3′-sulfate	C_15_H_10_O_10_S	32.872	[M – H]^−^	382.0021	380.9948	380.9951	0.8	301	* Bmc, Aec, Bethc, Dec, Eac
66	Quercetin 3-*O*-xylosyl-rutinoside	C_32_H_38_O_20_	33.752	[M + H]^−^	742.1933	743.2006	743.2003	−0.4	479, 317	Debc
Dihydroflavonols										
67	Dihydroquercetin 3-*O*-rhamnoside	C_21_H_22_O_11_	19.734	[M – H]^−^	450.1147	449.1074	449.1077	0.7	303	* Eec, Dethc, Dmc, Emc, Eabc, Dabc, Eabc
68	Dihydromyricetin 3-*O*-rhamnoside	C_21_H_22_O_12_	23.443	[M – H]^−^	466.1111	465.1038	465.1047	1.9	301	* Emc, Dac, Dethc, Eec
Dihydrochalcones										
69	3-Hydroxyphloretin 2′-*O*-glucoside	C_21_H_24_O_11_	4.663	[M – H]^−^	452.1348	451.1275	451.1277	0.4	289, 273	* Aac, Aec, Bec, Cac, Eec
Anthocyanins										
70	Delphinidin 3-*O*-glucoside	C_21_H_21_O_12_	26.489	[M – H]^−^	465.1016	464.0943	464.0940	−0.6	303	Bac
71	Pelargonidin	C_15_H_11_O_5_	32.266	[M – H]^−^	271.0618	270.0545	270.0557	4.4	243, 197, 169, 141	Eac
72	Cyanidin 3,5-*O*-diglucoside	C_27_H_31_O_16_	32.278	[M + H]^+^	611.1632	612.1705	612.1711	1.0	449, 287	* Aethbc, Bebc, Debc
73	Peonidin 3-*O*-diglucoside-5-*O*-glucoside	C_34_H_43_O_21_	33.383	[M – H]^−^	787.2331	786.2258	786.2246	−1.5	625, 478, 317	* Cethc, Dec
Isoflavonoids										
74	6″-*O*-Acetyldaidzin	C_23_H_22_O_10_	4.703	[M – H]^−^	458.1218	457.1145	457.1139	−1.3	221	Bmc
75	5,6,7,3′,4′-Pentahydroxyisoflavone	C_15_H_10_O_7_	5.266	[M – H]^−^	302.0439	301.0366	301.0376	3.3	285, 257	Amc
76	2′,7-Dihydroxy-4′,5′-dimethoxyisoflavone	C_17_H_14_O_6_	6.882	[M – H]^−^	314.0767	313.0694	313.0698	1.3	300, 282	* Eac, Eec
77	6″-*O*-Malonylgenistin	C_24_H_22_O_13_	14.363	** [M + H]^+^	518.1069	517.0996	517.0997	0.2	271	* Eac, Eac
78	2-Dehydro-*O*-desmethylangolensin	C_15_H_12_O_4_	16.548	[M – H]^−^	256.0746	255.0673	255.0667	−2.4	135, 119	* Emc, Bmc
79	Sativanone	C_17_H_16_O_5_	16.563	** [M – H]^−^	300.0994	299.0921	299.0923	0.7	284, 269, 225	* Aec, Aac, Amc, Bec, Cac, Emc, Ambc
80	6″-*O*-Malonylglycitin	C_25_H_24_O_13_	18.919	[M – H]^−^	532.1215	531.1142	531.1145	0.6	285, 270, 253	* Emc, Eec
81	Formononetin 7-*O*-glucuronide	C_22_H_20_O_10_	21.977	[M – H]^−^	444.1048	443.0975	443.0972	−0.7	267, 252	* Cac, Eac
82	2′-Hydroxyformononetin	C_16_H_12_O_5_	22.421	[M + H]^+^	284.0694	285.0767	285.0764	−1.1	270, 229	* Dmc, Cac, Emc
83	Violanone	C_17_H_16_O_6_	24.296	[M – H]^−^	316.0921	315.0848	315.0842	−1.9	300, 285, 135	* Aebc, Ambc
84	Genistein 4′,7-*O*-diglucuronide	C_27_H_26_O_17_	25.445	[M – H]^−^	622.1114	621.1041	621.1067	4.2	269	* Aac, Bec, Dethc, Debc
85	6″-*O*-Malonyldaidzin	C_24_H_22_O_12_	33.752	** [M + H]^+^	502.1093	503.1166	503.1178	2.4	255	* Debc, Dabc, Embc, Dac, Cac, Eac, Emc
Other polyphenols										
Hydroxycoumarins										
86	Esculetin	C_9_H_6_O_4_	24.191	[M – H]^−^	178.0248	177.0175	177.0171	−2.3	149, 133, 89	Bec
87	Scopoletin	C_10_H_8_O_4_	31.153	[M – H]^−^	192.0407	191.0334	191.0335	0.5	176	* Babc, Aebc, Bebc, Bethbc, Bmbc, Dabc, Debc, Dmbc, Dmc, Bec, Bmc, Cac, Eec, Emc
Hydroxybenzaldehydes										
88	*p*-Anisaldehyde	C_8_H_8_O_2_	30.932	[M – H]^−^	136.0516	135.0443	135.0442	−0.7	122, 109	* Bethc, Eac, Eec, Eethc, Emc
89	3-Hydroxy-3-(3-hydroxyphenyl) propionic acid	C_9_H_10_O_4_	32.498	[M – H]^−^	182.0582	181.0509	181.0507	−1.1	163, 135, 119	Eac
Phenolic terpenes										
90	Carnosic acid	C_20_H_28_O_4_	32.545	[M – H]^−^	332.1985	331.1912	331.1906	−1.8	287, 269	* Bac, Aac, Aec, Aethc, Amc, Bac, Bec, Bethc, Bmc, Cec, Dac, Dethc, Eec, Emc, Dethbc, Eabc
Tyrosols										
91	3,4-DHPEA-AC	C_10_H_12_O_4_	4.682	[M – H]^−^	196.0727	195.0654	195.0654	0.1	135	Cac
92	Hydroxytyrosol 4-*O*-glucoside	C_14_H_20_O_8_	16.175	[M – H]^−^	316.1149	315.1076	315.1076	0.1	153, 123	Cmbc
93	3,4-DHPEA-EDA	C_17_H_20_O_6_	28.718	[M – H]^−^	320.1280	319.1207	319.1209	0.6	275, 195	Bethbc
Alkylmethoxyphenols										
94	Equol	C_15_H_14_O_3_	18.110	** [M + H]^+^	242.0943	243.1016	243.1014	−0.8	255, 211, 197	* Cabc, Amc, Aac, Aec, Eac, Eethc
Other polyphenols										
95	Salvianolic acid B	C_36_H_30_O_16_	16.693	[M – H]^−^	718.1517	717.1444	717.1421	−3.2	519, 339, 321, 295	Embc
96	Lithospermic acid	C_27_H_22_O_12_	17.324	** [M – H]^−^	538.1097	537.1024	537.1018	−1.1	493, 339, 295	* Eabc, Cabc, Cethbc, Dmc, Cec
97	Salvianolic acid C	C_26_H_20_O_10_	21.929	[M – H]^−^	492.1052	491.0979	491.0989	2.0	311, 267, 249	* Debc, Dmbc
Lignans										
98	Todolactol A	C_20_H_24_O_7_	16.368	[M – H]^−^	376.1539	375.1466	375.1470	1.1	313, 137	* Debc, Embc
99	7-Hydroxymatairesinol	C_20_H_22_O_7_	28.718	** [M – H]^−^	374.1379	373.1306	373.1311	1.3	343, 313, 298, 285	* Bethbc, Dabc, Eebc, aac
100	Arctigenin	C_21_H_24_O_6_	30.493	[M – H]^−^	372.1565	371.1492	371.1475	−4.6	356, 312, 295	Aethc
101	Secoisolariciresinol-sesquilignan	C_30_H_38_O_10_	30.674	[M – H]^−^	558.2463	557.2390	557.2390	0.1	539, 521, 509, 361	Aec, debc
102	7-Oxomatairesinol	C_20_H_20_O_7_	32.347	[M + H]^+^	372.1184	373.1257	373.1256	−0.3	358, 343, 328, 325	* Babc, Bebc, Bethbc, Cmbc
103	Sesamin	C_20_H_18_O_6_	32.442	[M – H]^−^	354.1124	353.1051	353.1061	2.8	338, 163	Eec
Stilbenes										
104	Resveratrol 5-*O*-glucoside	C_20_H_22_O_8_	16.382	[M – H]^−^	390.1310	389.1237	389.1234	−0.8	227	Embc

* Compound was detected in more than one seaweed samples, data presented in this table are from asterisk sample. ** Compounds were detected in both negative [M – H]^−^ and positive [M + H]^+^ mode of ionization while only single mode data was presented. Seaweed samples were mentioned in abbreviations.

## Data Availability

The data presented in this study are available in this article.

## Supplementary Materials

The following supporting information can be downloaded at: https://www.mdpi.com/article/10.3390/ph16050773/s1, Table S1: Estimation of freeze-dried free phenolics of seaweed species extracted by the conventional and non-conventional method; Table S2: Estimation of freeze-dried bound phenolics of seaweed species extracted by the conventional and non-conventional method; Table S3: Estimation of freeze-dried free phenolic’s antioxidant potential of seaweed species extracted by the conventional and non-conventional method; Table S4: Estimation of freeze-dried bound antioxidant potential of seaweed species extracted by the conventional and non-conventional method; Table S5: Characterization of free phenolic compounds in different seaweed samples with different extraction methods by LC-ESI-QTOF-MS/MS; Table S6: Characterization of bound phenolic compounds in different seaweed samples with different extraction methods by LC-ESI-QTOF-MS/MS.

## Abbreviations

Amu: *Phyllospora comosa* methanol ultrasound extract; Aeu: *Phyllospora comosa* ethanol ultrasound extract; Aau: *Phyllospora comosa* acetone ultrasound extract; Aethu: *Phyllospora comosa* ethyl acetate ultrasound extract; Bmu: *Ecklonia radiata* methanol ultrasound extract; Beu: *Ecklonia radiata* ethanol ultrasound extract; Bau: *Ecklonia radiata* acetone ultrasound extract; Bethu: *Ecklonia radiata* ethyl acetate ultrasound extract; Cmu: *Durvillaea* sp., methanol ultrasound extract; Ceu: *Durvillaea* sp., ethanol ultrasound extract; Cau: *Durvillaea* sp., acetone ultrasound extract; cethu: *Durvillaea* sp., ethyl acetate ultrasound extract; Dmu: *Sargassum* sp., methanol ultrasound extract; Deu: *Sargassum* sp., ethanol ultrasound extract; Dau: *Sargassum* sp., acetone ultrasound extract; Dethu: *Sargassum* sp., ethyl acetate ultrasound extract; Emu: *Cystophora* sp., methanol ultrasound extract; Eeu: *Cystophora* sp., ethanol ultrasound extract; Eau: *Cystophora* sp., acetone ultrasound extract; Eethu: *Cystophora* sp., ethyl acetate ultrasound extract; Ambu: *Phyllospora comosa* methanol bound ultrasound extract; Aebu: *Phyllospora comosa* ethanol bound ultrasound extract; Aabu: *Phyllospora comosa* acetone bound ultrasound extract; Aethbu: *Phyllospora comosa* ethyl acetate bound ultrasound extract; Bmbu: *Ecklonia radiata* methanol bound ultrasound extract; Bebu: *Ecklonia radiata* ethanol bound ultrasound extract; Babu: *Ecklonia radiata* acetone bound ultrasound extract; Bethbu: *Ecklonia radiata* ethyl acetate bound ultrasound extract; Cmbu: *Durvillaea* sp., methanol bound ultrasound extract; Cebu: *Durvillaea* sp., ethanol bound ultrasound extract; Cabu: *Durvillaea* sp., acetone bound ultrasound extract; Cethbu: *Durvillaea* sp., ethyl acetate bound ultrasound extract; Dmbu: *Sargassum* sp., methanol bound ultrasound extract; Debu: *Sargassum* sp., ethanol bound ultrasound extract; Dabu: *Sargassum* sp., acetone bound ultrasound extract; Dethbu: *Sargassum* sp., ethyl acetate bound ultrasound extract; Embu: *Cystophora* sp., methanol bound ultrasound extract; Eebu: *Cystophora* sp., ethanol bound ultrasound extract; Eabu: *Cystophora* sp., acetone bound ultrasound extract; Eethbu: *Cystophora* sp., ethyl acetate bound ultrasound extract.
