# Supplementary material for: Comparative Study on the Effect of Phenolics and Their Antioxidant Potential of Freeze-Dried Australian Beach-Cast Seaweed Species upon Different Extraction Methodologies
Source: Pharmaceuticals (Basel). 2023 May 22;16(5):773. doi: 10.3390/ph16050773 (PMC10224432; doi:10.3390/ph16050773)
Supplement: Supplementary file 1 [file pharmaceuticals-16-00773-s001.zip › pharmaceuticals-2395025-supplementary.pdf]

pharmaceuticals

Article

Comparative Study on the Effect of Phenolics and Their Antioxidant Potential of Freeze-Dried Australian Beach-Cast Seaweed Species upon Different Extraction Methodologies

Vigasini Subbiah [1,2], Faezeh Ebrahimi [2], Osman T. Agar [2], Frank R. Dunshea [2,3], Colin J. Barrow [1] and Hafiz A. R. Suleria [1,2,*]

[1] Centre for Sustainable Bioproducts, Deakin University, Waurn Ponds, VIC 3217, Australia; vsubbiah@deakin.edu.au (V.S.); colin.barrow@deakin.edu.au (C.J.B.)
[2] School of Agriculture, Food and Ecosystem Sciences, Faculty of Science, The University of Melbourne, Parkville, VIC 3010, Australia; ebrahimif@student.unimelb.edu.au (F.E.); osman.agar@unimelb.edu.au (O.T.A.); fdunshea@unimelb.edu.au (F.R.D.)
[3] Faculty of Biological Sciences, The University of Leeds, Leeds LS2 9JKT, UK
* Correspondence: hafiz.suleria@unimelb.edu.au; Tel.: +61-470-439-670

Abstract: Brown seaweeds are rich in phenolic compounds and have high health benefits. However, the phenolics present in Australian beach-cast seaweed are still unclear. This study investigated the effect of ultrasonication and conventional methodologies by four different solvents on free and bound phenolics of freeze-dried brown seaweed species obtained from the southeast Australian shoreline. The phenolic content and their antioxidant potential were determined by *in vitro* assays followed by identification and characterization by LC-ESI-QTOF-MS/MS and quantified by HPLC-PDA. The *Cystophora* sp., displayed high total phenolic content (TPC) and phlorotannin content (FDA) when extracted using 70% ethanol (ultrasonication method). *Cystophora* sp., also exhibited strong antioxidant potential in various assays, such as DPPH, ABTS, and FRAP in 70% acetone through ultrasonication. TAC is highly correlated to FRAP, ABTS, and RPA ($p < 0.05$) in both extraction methodologies. LC-ESI-QTOF-MS/MS analysis identified 94 and 104 compounds in ultrasound and conventional methodologies, respectively. The HPLC-PDA quantification observed phenolic acids to be higher in ultrasonication methodology. Our findings would facilitate the development of nutraceuticals, pharmaceuticals, and functional foods.

Keywords: seaweeds; freeze-drying; conventional extraction; ultrasonication; phenolic compounds; antioxidant activity; LC-ESI-QTOF-MS/MS, HPLC-PDA

Citation: Subbiah, V.; Ebrahimi, F.; Agar, O.T.; Dunshea, F.R.; Barrow, C.J.; Suleria, H.A.R. Comparative Study on the Effect of Phenolics and Their Antioxidant Potential of Freeze-Dried Australian Beach-Cast Seaweed Species upon Different Extraction Methodologies. *Pharmaceuticals* **2023**, *16*, 773. https://doi.org/10.3390/ph16050773

Academic Editors: Noelia Duarte and Isabel Ribeiro

Received: 27 April 2023
Revised: 17 May 2023
Accepted: 18 May 2023
Published: 22 May 2023

Copyright: © 2023 by the authors. Submitted for possible open access publication under the terms and conditions of the Creative Commons Attribution (CC BY) license (https://creativecommons.org/licenses/by/4.0/).

Table S1: Estimation of freeze-dried free phenolics of seaweed species extracted by the conventional and non-conventional method.

Samples	Solvents	TPC (mg GAE/g)	TFC (mg QE/g)	TCT (mg CE/g)	DMBA (PGE mg/g)	PBA (PGE mg/g)	FDA (PGE mg/g)
**Ultrasonication Extraction**							
Cystophora sp.	70% ACE	17.63 ± 0.09^{Aa}	1.60 ± 0.01^{Aa}	-	1.75±0.08^{Ea}	1.78±0.30^{Fe}	9.37±0.72^{Aa}
	70% MeOH	15.60 ± 0.02^{Ba}	0.40 ± 0.01^{Da}	-	0.84±0.04^{Ga}	2.85±0.06^{Ee}	1.79±0.04^{Ie}
	70% EtOH	14.91 ± 1.32^{Ba}	1.20 ± 0.01^{Ca}	2.21 ± 0.07^{Ba}	1.17±0.02^{Fa}	3.36±0.48^{Dd}	7.74±0.14^{Ca}
	EA	-	0.15 ± 0.01^{HIb}	-	4.04±0.01^{Bb}	4.95±0.25^{CDa}	-
Phyllospora comosa	70% ACE	14.60 ± 0.85^{BCb}	1.02 ± 0.01^{Kb}	-	0.39±0.2^{IJd}	5.28±0.29^{Cc}	8.24±0.90^{Bb}
	70% MeOH	13.39 ± 0.11^{Cb}	0.15 ± 0.01^{Hd}	0.70 ± 0.03^{Db}	0.47±0.01^{Ib}	11.55±0.26^{Aa}	6.19±0.17^{DEa}
	70% EtOH	11.28 ± 0.02^{Db}	0.57 ± 0.01^{Cb}	-	0.42±0.01^{Ib}	3.13±0.22^{De}	6.36±0.42^{Db}
	EA	0.38 ± 0.01^{Ib}	0.09 ± 0.01^{Jc}	-	4.82±0.67^{Aa}	-	-
Sargassum sp.	70% ACE	13.45 ± 0.72^{Cb}	0.31 ± 0.01^{Fc}	-	0.64±0.05^{Hc}	5.97±0.06^{BCa}	1.83±0.02^{He}
	70% MeOH	8.96 ± 0.33^{Ec}	0.36 ± 0.01^{Eb}	-	0.46±0.05^{Ib}	3.41±0.07^{Dc}	2.71±0.10^{GHc}
	70% EtOH	9.56 ± 0.56^{Eb}	0.41 ± 0.01^{Dc}	0.10 ± 0.03^{Ec}	0.42±0.11^{Ib}	5.66±0.17^{BCc}	0.28±0.01^{Jd}
	EA	0.34 ± 0.03^{Ib}	0.05 ±0.01^{Kd}	-	-	-	-
Ecklonia radiata	70% ACE	8.48 ± 0.17^{EFc}	0.23 ± 0.01^{Gd}	-	0.87±0.1^{Gb}	5.41±0.25^{Cb}	5.03±0.24^{EFd}
	70% MeOH	8.65 ± 0.41^{Ec}	0.23 ± 0.01^{Gc}	-	0.84±0.07^{Ga}	5.94±0.37^{BCb}	2.35±0.25^{Hd}
	70% EtOH	7.16 ± 0.10^{Fc}	0.24 ± 0.01^{Gd}	-	1.14±0.02^{Fa}	6.05±0.14^{BCb}	3.01±0.30^{Gc}
	EA	0.11 ± 0.01^{Ic}	0.28 ± 0.01^{Ga}	-	3.79±0.31^{Cc}	-	-
Durvillaea sp.	70% ACE	8.23 ± 0.67^{EFc}	0.04 ± 0.01^{Ke}	-	0.49±0.02^{Id}	5.16±0.14^{Cd}	5.45±0.24^{Ec}
	70% MeOH	4.13 ± 0.09^{Hd}	0.05 ± 0.01^{Ke}	2.73 ± 0.07^{Aa}	0.32±0.02^{IJc}	3.24±0.48^{Dd}	4.36±0.29^{Fb}
	70% EtOH	5.65 ± 0.44^{Gc}	0.12 ± 0.01^{Ie}	1.78 ± 0.04^{Cb}	0.39±0.01^{IJb}	6.63±0.58^{Ba}	3.01±0.25^{Gc}
	EA	4.89 ± 0.02^{GHa}	0.03 ±0.01^{Kd}	-	3.29±0.12^{Dd}	-	-
**Conventional Extraction**							
Cystophora sp.	70% ACE	14.77 ± 1.06 ^{Aa}	1.69 ± 0.01^{Aa}	-	0.48±0.04^{EFa}	0.09±0.21^{Je}	6.53±0.55^{Cb}
	70% MeOH	13.98 ± 0.58^{Aa}	0.55 ± 0.01^{Da}	2.33 ± 0.07^{Ba}	0.81±0.26^{DEb}	4.44±0.10^{Dc}	7.27±0.43^{BCa}
	70% EtOH	13.74 ± 0.58^{Aa}	1.47 ± 0.01^{Ba}	1.20 ± 0.07^{Ca}	0.67±0.14^{Eb}	5.66±0.25^{Cb}	8.10±0.55^{Ba}
	EA	-	0.04 ± 0.01^{JKc}	-	-	-	-
Sargassum sp.	70% ACE	10.24 ± 0.41^{Cc}	0.20 ± 0.01^{Gd}	-	0.50±0.01^{EFa}	3.96±0.13^{Ea}	2.13±0.10^{Fd}
	70% MeOH	8.82 ± 0.63^{Dc}	0.45 ± 0.01^{Eb}	-	0.48±0.03^{EFc}	6.06±0.15^{Ba}	2.38±0.1^{EFb}
	70% EtOH	6.49 ± 0.15^{Fc}	0.07 ± 0.01^{IJd}	-	0.47±0.01^{EFc}	3.00±0.70^{F}	-
	EA	-	-	-	4.91±0.75^{Bb}	0.24±0.06^{IJ}	-
Phyllospora comosa	70% ACE	13.04 ± 0.50^{Bb}	0.75 ± 0.01^{Cb}	-	0.33±0.01^{Fb}	2.28±0.35^{GHd}	1.42±0.12^{Ge}
	70% MeOH	10.42 ± 0.81^{Cb}	0.09 ± 0.01^{HId}	-	0.27±0.01^{FGc}	1.27±0.22^{Hde}	1.57±0.01^{Gd}
	70% EtOH	9.43± 0 .59^{Cb}	0.33 ± 0.01^{Fc}	-	0.40±0.01^{EFc}	6.69±0.10^{Aa}	4.15±0.21^{Db}

	EA	-	0.07 ± 0.01^{IJb}	-	4.24±0.04^{Cc}	2.39±0.20^{GH}	-
Ecklonia radiata	70% ACE	7.86 ± 0.30^{Ed}	0.10 ± 0.01^{He}	-	0.48±0.04^{EFa}	2.54±0.05^{Gc}	3.12±0.29^{Ec}
	70% MeOH	7.16 ± 0.37^{Fd}	0.09 ± 0.01^{HIc}	-	1.01±0.12^{Da}	0.54±0.11^{Ie}	2.01±0.19^{Fc}
	70% EtOH	5.60 ± 0.20^{Gcd}	0.23 ± 0.01^{Gcc}	-	0.78±0.08^{DEa}	4.65±0.08^{Dd}	1.35±0.01^{Gd}
	EA	-	0.09 ± 0.01^{HIa}	27.75 ± 0.23^{Aa}	5.08±0.31^{Aa}	0.10±0.34^{J}	-
Durvillaea sp.	70% ACE	7.37 ± 0.20^{Fd}	0.32 ± 0.03^{Fc}	-	0.29±0.08^{FGb}	2.72±0.10^{FGb}	19.64±0.24^{Aa}
	70% MeOH	3.83 ± 0.15^{Ie}	0.01 ± 0.01^{Ld}	1.17 ± 0.09^{Cb}	0.29±0.08^{FGc}	5.84±0.02^{BCb}	0.02±0.01^{He}
	70% EtOH	4.98 ± 0.33^{Hd}	0.04 ± 0.01^{JKe}	0.37 ± 0.07^{Db}	0.39±0.05^{Fc}	4.76±0.08^{Dc}	2.71±0.27^{DEc}
	EA	-	0.02 ± 0.01^{KLd}	-	0.49±0.01^{EFd}	-	-

All values are expressed as the mean ± SD and performed in triplicates. Different letters ($^{a, b, c, d, e}$) within the same column are significantly different ($p < 0.05$) samples within the solvent whereas letters ($^{A, B, C, D, E}$) within the same column are significantly different ($p < 0.05$) samples within the species. Six species of seaweed are reported based on dry weight. CE (catechin equivalents), QE (quercetin equivalents), GAE (gallic acid equivalents), PGE (phloroglucinol equivalents). TFC (total flavonoids content), TPC (total phenolic content), TCT (total tannins content), DMBA (2,4-dimethoxybenzaldehyde assay), PBA (Prussian blue assay), FDA (Folin-Denis Assay). The abbreviation of solvents expressed are ACE (Acetone), MeOH (Methanol), EtOH (Ethanol), EA (Ethyl Acetate).

Table S2: Estimation of freeze-dried bound phenolics of seaweed species extracted by the conventional and non-conventional method.

Samples	Solvents	TPC (mg GAE/g)	TFC (mg QE/g)	TCT (mg CE/g)	DMBA (PGE mg/g)	PBA (PGE mg/g)	FDA (PGE mg/g)
				**Ultrasonication Extraction**			
Cystophora sp.	ACE	1.72 ± 0.04^{GHb}	0.68 ± 0.05^{Hbc}	0.67 ± 0.06^{GHc}	0.21±0.01^{Fc}	0.83±0.06^{EFb}	1.84±0.17^{Cb}
	MeOH	7.14 ± 0.67^{Ca}	2.94 ± 0.14^{CDb}	-	0.18±0.01^{Fc}	5.76±0.07^{Aa}	2.07±0.10^{Ba}
	EtOH	14.01 ± 0.68^{Aa}	2.63 ± 0.22^{CDEb}	-	0.46±0.01^{Ea}	4.75±0.39^{Ba}	6.46±0.61^{Aa}
	EA	1.66 ± 0.03^{Jd}	6.99 ± 0.1^{EFGHc}	0.94 ± 0.18^{GHa}	0.11±0.01^{FGe}	0.10±0.01^{Hd}	-
Phyllospora comosa	ACE	1.51 ± 0.05^{Hb}	0.85 ± 0.03^{GHb}	0.94 ± 0.07^{DEb}	4.57±0.45^{Ba}	0.90±0.04^{EFa}	0.33±0.03^{GHe}
	MeOH	3.46 ± 0.12^{DEb}	3.29 ±0.18^{Cb}	2.06 ± 0.17^{Ba}	0.20±0.01^{Fb}	1.07±0.01^{Ed}	0.39±0.02^{Ge}
	EtOH	1.13 ± 0.03^{HId}	3.05 ± 0.11^{Ca}	2.18 ± 0.16^{Aa}	0.04±0.01^{Gc}	0.07±0.01^{Hc}	-
	EA	1.85 ± 0.13^{GHb}	3.46 ± 0.31^{Cbc}	0.77 ± 0.06^{EFb}	1.83±0.09^{Dd}	0.13±0.01^{GHd}	-
Sargassum sp.	ACE	1.15 ± 0.09^{Hc}	0.36 ± 0.03^{Hd}	0.27 ± 0.02^{Id}	0.25±0.02^{Fb}	0.53±0.04^{FGc}	2.20±0.09^{Ba}
	MeOH	7.41 ± 0.17^{BCa}	1.03 ± 0.04^{FGHc}	-	0.22±0.01^{Fa}	4.36±0.14^{BCb}	0.99±0.24^{Db}
	EtOH	7.97 ± 0.53^{Bb}	2.38 ± 0.05^{CDEb}	0.53 ± 0.04^{Ib}	0.30±0.01^{EFb}	1.19±0.10^{Eb}	0.38±0.05^{Gd}
	EA	-	2.55 ± 0.15^{CDEbc}	-	3.62±0.32^{Cc}	1.18±0.06^{Ea}	-
Ecklonia radiata	ACE	2.71 ± 0.08^{EFa}	0.61 ± 0.03^{Hc}	1.29 ± 0.01^{Ca}	0.15±0.01^{FGd}	0.45±0.04^{FGd}	0.70±0.03^{Ed}
	MeOH	3.54 ± 0.04^{Db}	10.92 ± 0.97^{Ba}	0.92 ± 0.09^{DEb}	0.12±0.01^{FGd}	2.06±0.02^{Cc}	0.85±0.04^{DEc}
	EtOH	1.87 ± 0.16^{GHd}	1.42 ± 0.13^{DEFGHc}	-	0.04±0.01^{Gd}	0.08±0.01^{Hc}	0.43±0.01^{FGc}
	EA	1.43 ± 0.07^{Hc}	3.76 ± 0.05^{Cb}	-	4.50±0.36^{Bb}	0.92±0.03^{EFb}	-
Durvillaea sp.	ACE	1.75 ± 0.15^{GHb}	3.31 ± 0.15^{Ca}	1.04 ± 0.07^{Db}	0.08±0.01^{Ge}	0.20±0.01^{Ge}	1.47±0.11^{CDc}
	MeOH	0.32 ± 0.01^{IJc}	1.27 ± 0.1^{EFGHc}	-	0.03±0.01^{GHe}	0.63±0.02^{Fe}	0.48±0.04^{Fd}
	EtOH	2.95 ± 0.19^{DEFc}	3.04 ± 0.04^{Ca}	0.13 ± 0.01^{HIb}	0.01±0.01^{GHd}	-	0.62±0.06^{EFb}
	EA	2.37 ± 0.20^{FGa}	18.20 ± 0.56^{Aa}	-	4.93±0.14^{Aa}	0.48±0.02^{FGc}	-
				**Conventional Extraction**			
Cystophora sp.	ACE	3.5 ± 0.34^{Cb}	0.78 ± 0.20^{Ga}	0.15 ± 0.01^{FGc}	0.1±0.01^{DEb}	0.39±0.02^{CDb}	0.64±0.13^{Dd}
	MeOH	4.61 ± 0.95^{Aa}	1.28 ± 0.03^{Ca}	0.22 ± 0.04^{EFc}	0.13±0.01^{Da}	2.91±0.22^{Aa}	1.67±0.29^{Aa}
	EtOH	1.75 ± 0.24^{Db}	1.01 ± 0.26^{EFab}	0.2 ± 0.05^{FGb}	0.35±0.02^{Ca}	0.54±0.1^{Cb}	0.34±0.01^{Ed}
	EA	0.96 ± 0.10^{EFa}	2.47 ± 0.45^{Bb}	0.17 ± 0.02^{FGa}	-	-	-
Sargassum sp.	ACE	3.7 ± 0.61^{Ba}	0.14 ± 0.03^{JKc}	0.28 ± 0.03^{DEb}	0.15±0.01^{Da}	0.75±0.19^{Ba}	0.92±0.30^{Cc}
	MeOH	1.55 ± 0.12^{Db}	0.93 ± 0.07^{EFc}	0.43 ± 0.11^{Cb}	0.06±0.01^{DEc}	0.98±0.09^{ABb}	0.53±0.08^{DEc}
	EtOH	2.4 ± 0.43^{Ca}	0.89 ± 0.18^{FGbc}	0.13 ± 0.01^{Gd}	0.11±0.02^{Db}	0.7±0.19^{BCa}	0.69±0.13^{Db}
	EA	0.52 ± 0.10^{EFGHc}	6.35 ± 0.05^{Aa}	-	1.41±0.01^{Bb}	-	-
Ecklonia radiata	ACE	2.92 ± 0.35^{Ec}	0.25 ± 0.01^{IJb}	-	0.05±0.01^{DEc}	-	0.45±0.08^{Ee}
	MeOH	3.47 ± 0.81^{EFGc}	0.03 ± 0.01^{CDb}	-	0.06±0.01^{DEc}	0.21±0.03^{Dd}	0.35±0.04^{Ed}
	EtOH	3.14± 0 .59^{EFGHc}	0.11 ± 0.01^{DEa}	-	0.03±0.01^{DEd}	0.26±0.07^{Dc}	0.54±0.06^{DEc}

	EA	-	0.02 ± 0.01^{Cc}	-	1.71 ± 0.01^{Aa}	-	-
Phyllospora comosa	ACE	0.68 ± 0.11^{EFGHd}	0.32 ± 0.06^{Ib}	0.02 ± 0.01^{Hd}	0.11 ± 0.03^{Da}	0.16 ± 0.01^{DEc}	1.44 ± 0.10^{Ba}
	MeOH	0.6 ± 0.09^{EFGHcd}	0.13 ± 0.02^{Kd}	0.59 ± 0.13^{Ba}	0.11 ± 0.01^{Db}	0.94 ± 0.08^{ABb}	1.52 ± 0.11^{ABb}
	EtOH	0.85 ± 0.16^{EFGc}	0.58 ± 0.04^{Hd}	0.14 ± 0.04^{Gd}	0.13 ± 0.01^{Dc}	0.3 ± 0.08^{CDc}	1.47 ± 0.09^{Ba}
	EA	0.68 ± 0.11^{EFGHb}	6.45 ± 0.09^{Aa}	-	1.49 ± 0.09^{Bb}	0.37 ± 0.01^{CDa}	-
Durvillaea sp.	ACE	0.8 ± 0.15^{EFGcd}	0.67 ± 0.04^{IJKbc}	0.8 ± 0.22^{Aa}	0.03 ± 0.01^{DEd}	0.09 ± 0.01^{Ed}	1.40 ± 0.02^{Bb}
	MeOH	0.48 ± 0.70^{GHIJd}	0.03 ± 0.01^{Kd}	0.44 ± 0.11^{Cb}	-	0.36 ± 0.04^{CDc}	0.51 ± 0.04^{DEc}
	EtOH	0.03 ± 0.01^{Jd}	0.74 ± 0.06^{Gcc}	0.17 ± 0.05^{FGc}	-	0.07 ± 0.01^{Ed}	0.20 ± 0.01^{Fe}
	EA	0.09 ± 0.01^{IJe}	6.33 ± 0.01^{Aa}	-	0.41 ± 0.03^{Cc}	0.02 ± 0.01^{Eb}	-

All values are expressed as the mean ± SD and performed in triplicates. Different letters ($^{a, b, c, d, e}$) within the same column are significantly different ($p < 0.05$) samples within the solvent whereas letters ($^{A, B, C, D, E}$) within the same column are significantly different ($p < 0.05$) samples within the species. Six species of seaweed are reported based on dry weight. CE (catechin equivalents), QE (quercetin equivalents), GAE (gallic acid equivalents), PGE (phloroglucinol equivalents). TFC (total flavonoids content), TPC (total phenolic content), TCT (total tannins content), DMBA (2,4-dimethoxybenzaldehyde assay), PBA (Prussian blue assay), FDA (Folin-Denis Assay). The abbreviation of solvents expressed are ACE (Acetone), MeOH (Methanol), EtOH (Ethanol), EA (Ethyl Acetate).

Table S3: Estimation of freeze-dried free phenolic's antioxidant potential of seaweed species extracted by the conventional and non-conventional method.

Samples	Solvents	DPPH (mg TE/g)	FRAP (mg TE/g)	ABTS (mg TE/g)	FICA (mg EDTA/g)	·OH-RSA (mg TE/g)	TAC (mg TE/g)	RPA (mg TE/g)
**Ultrasonication Extraction**								
Cystophora sp.	70% ACE	47.70 ± 0.07^{Aa}	38.46 ± 0.04^{Aa}	53.98 ± 0.20^{Aa}	0.8 ± 0.01^{C-Eb}	10.23 ± 0.21^{Ec}	73.74±0.30^{Aa}	8.60 ± 0.02^{Id}
	70% MeOH	41.03 ± 0.07^{Ba}	19.84 ± 0.22^{Fb}	48.54± 0.18^{Ba}	0.03 ± 0.01^{Fc}	8.95 ± 0.14^{Fc}	18.71 ± 1.04^{Ic}	10.51 ± 0.05^{Gc}
	70% EtOH	4.96 ± 0.01^{Ib}	16.81 ± 0.22^{Hc}	34.21 ± 0.18^{Ca}	0.22 ± 0.01^{EFb}	-	31.94 ± 1.20^{Fb}	23.64 ± 0.12^{Ba}
	EA	-	-	-	0.97 ± 0.01^{CDa}	-	-	-
Phyllospora comosa	70% ACE	37.52 ± 0.07^{Cb}	15.63 ± 0.12^{Id}	14.65 ± 0.84^{Gd}	3.09 ± 0.11^{Aa}	41.64 ± 1.10^{Ba}	48.38 ± 0.76^{Cc}	25.45 ± 0.09^{Aa}
	70% MeOH	4.76 ± 0.01^{HIc}	4.79 ± 0.23^{Kd}	24.36 ± 0.27^{Ec}	0.99 ± 0.01^{CDa}	53.07 ± 0.96^{Aa}	32.84 ± 0.60^{Fa}	18.22 ± 0.18^{Da}
	70% EtOH	4.69 ± 0.01^{Gd}	9.96 ± 0.19^{Jd}	24.18 ± 0.10^{Eb}	0.95 ± 0.01^{CDa}	4.93 ± 0.11^{Ga}	40.46 ± 0.92^{Da}	7.24 ± 0.04^{Ke}
	EA	0.02 ± 0.01^{Ja}	-	0.46 ± 0.01^{Ja}	0.01 ± 0.04^{Fb}	-	-	-
Sargassum sp.	70% ACE	35.40 ± 0.03^{Dc}	29.29 ± 0.23^{Bb}	35.03±0.40^{Cb}	0.53 ± 0.01^{D-Fbc}	13.72 ± 0.36^{Db}	53.79 ± 0.35^{Bbb}	22.47 ± 0.12^{Cb}
	70% MeOH	4.70 ± 0.01^{Ic}	26.62 ± 0.20^{Ca}	20.03±0.31^{Fd}	0.21 ± 0.01^{EFb}	-	30.64 ± 0.63^{FGb}	8.82 ± 0.03^{Ib}
	70% EtOH	4.68 ± 0.01^{Id}	21.12 ± 0.16^{Ea}	20.15 ± 0.75^{Fc}	0.21 ± 0.01^{EFb}	-	29.63 ± 0.46^{Gb}	14.10 ± 0.18^{Fb}
	EA	-	0.06 ± 0.01^{Ma}	0.27 ± 0.03^{Jb}	0.02 ± 0.02^{DEFb}	-	-	-
Ecklonia radiata	70% ACE	33.33 ± 0.05^{Ed}	21.83 ± 0.12^{Dc}	15.03 ± 0.45^{Gd}	0.87 ± 0.01^{C-Dbc}	41.16 ± 0.72^{Ba}	37.45 ± 1.51^{Ea}	10.34 ± 0.03^{GHc}
	70% MeOH	5.01 ± 0.01^{Gb}	16.66 ± 0.09^{Hc}	31.14 ± 0.99^{Db}	-	-	12.19 ± 0.76^{Jd}	16.56 ± 0.08^{Eb}
	70% EtOH	5.06 ± 0.02^{Ga}	17.44 ± 0.16^{Gb}	11.64 ± 0.68^{Hd}	0.32 ± 0.01^{D-Fb}	-	25.52 ± 1.51^{Hc}	8.02 ± 0.08^{Jd}
	EA	-	-	-	1.48 ± 0.01^{BCa}	-	-	-
Durvillaea sp.	70% ACE	20.54 ± 0.04^{Fe}	1.81 ± 0.08^{Le}	18.87 ± 0.07^{Fc}	0.42 ± 0.01^{D-Fc}	-	17.31 ± 0.08^{Ie}	0.79 ± 0.16^{Le}
	70% MeOH	4.69 ± 0.01^{Ic}	4.86 ± 0.12^{Kd}	9.94±0.02^{Ie}	0.21 ± 0.01^{EFb}	16.17 ± 0.01^{Cb}	5.44 ± 0.15^{Ke}	10.24 ± 0.04^{Hd}
	70% EtOH	4.82 ± 0.01^{Hc}	10.21 ± 0.08^{Jd}	11.08±0.40^{HId}	0.22 ± 0.01^{EFb}	4.12 ± 0.07^{Gb}	6.95 ± 0.15^{Kd}	10.25 ± 0.05^{GHc}
	EA	-	0.06 ± 0.01^{Ma}	-	1.81 ± 0.01^{Ba}	-	-	-
**Conventional Extraction**								
Cystophora sp.	70% ACE	45.65 ± 0.08^{Aa}	30.42 ± 0.29^{Aa}	39.09 ± 0.22^{Aa}	-	-	77.45 ± 0.17^{Aa}	6.80 ± 0.03^{Kd}
	70% MeOH	41.74 ± 0.07^{Ca}	29.97 ± 0.12^{Aa}	37.78 ± 0.13^{Ba}	0.01 ± 0.01^{Id}	-	40.46 ± 0.46^{Ca}	11.29 ± 0.02^{Ba}
	70% EtOH	45.34 ± 0.07^{Ba}	31.35 ± 0.23^{Ea}	38 ± 0.32^{Aa}	0.19 ± 0.01^{Hd}	-	73.44 ± 0.30^{Ba}	20.74 ± 0.09^{Aa}
	EA	3.15 ± 0.06^{Kb}	-	-	0.84 ± 0.01^{Dd}	-	-	-
Sargassum sp.	70% ACE	32.11 ± 0.16^{Ec}	28.00 ± 0.16^{Bb}	20.06 ± 0.27^{Cb}	0.17 ± 0.01^{Hc}	-	33.74 ± 0.52^{Db}	8.08 ± 0.07^{Fb}
	70% MeOH	4.86 ± 0.04^{Ic}	15.68 ± 0.23^{Db}	19.97 ± 0.48^{Bc}	0.19 ± 0.01^{Hc}	-	8.58 ± 0.17^{Jd}	6.75 ± 0.03^{Kd}
	70% EtOH	4.91 ± 0.03^{Ic}	14.85 ± 0.31^{Ea}	18.16 ± 0.46^{Db}	0.17 ± 0.01^{He}	-	13.99 ± 0.17^{Id}	7.66 ± 0.03^{Ad}
	EA	2.88 ± 0.19^{Lc}	-	-	0.21 ± 0.02^{He}	-	-	-
Phyllospora comosa	70% ACE	35.02 ± 0.09^{Db}	12.12 ± 0.23^{Fd}	17.37 ± 0.86^{DEc}	0.80 ± 0.01^{Da}	-	18.01 ± 0.63^{Hd}	7.41 ± 0.03^{Ic}
	70% MeOH	4.74 ± 0.01^{IJd}	6.02 ± 0.12^{Jd}	9.57 ± 0.03^{Fb}	0.56 ± 0.01^{Ea}	20.26 ± 0.08^{Aa}	13.81 ± 0.31^{Ic}	9.40 ± 0.04^{Eb}
	70% EtOH	4.93 ± 0.01^{Ic}	8.67 ± 0.29^{Ec}	9.93 ± 0.04^{Fc}	0.99 ± 0.01^{Ba}	3.32 ± 0.13^{Db}	21.78 ± 0.09^{Fb}	10.62 ± 0.02^{Hb}

	EA	-	0.05 ± 0.01^{La}	-	I0.92 ± 0.07^{Ca}	-	-	0.42 ± 0.02^{Na}
Ecklonia radiata	70% ACE	30.29 ± 0.07^{Fd}	16.84 ± 0.12^{Cc}	19.56 ± 0.58^{Cb}	0.39 ± 0.01^{Fc}	2.17 ± 0.01^{Ea}	21.91 ± 0.17^{Fc}	6.24 ± 0.02^{Le}
	70% MeOH	30.01 ± 0.07^{Gb}	10.31 ± 0.12^{Hc}	16.43 ± 0.40^{Ec}	0.18 ± 0.01^{Hb}	-	23.49 ± 0.15^{Eb}	7.04 ± 0.02^{Jc}
	70% EtOH	27.77 ± 0.12^{Hb}	11.17 ± 0.19^{Gb}	10.44 ± 0.23^{Fc}	0.33 ± 0.01^{FGc}	-	20.98 ± 0.23^{Gc}	7.89 ± 0.02^{Gc}
	EA	2.51 ± 0.04^{Md}	-	-	4.25 ± 0.07^{Ac}	-	-	-
Durvillaea sp.	70% ACE	30.29 ± 0.05^{Fd}	9.98 ± 0.08^{He}	9.86 ± 0.01^{Fd}	0.20 ± 0.01^{Hc}	-	13.31 ± 0.09^{Ie}	9.65 ± 0.03^{Da}
	70% MeOH	4.70 ± 0.02^{IJd}	4.91 ± 0.20^{Ke}	9.75 ± 0.02^{Fd}	0.30 ± 0.01^{Gb}	15.60 ± 0.14^{Bb}	4.09 ± 0.04^{Le}	4.17 ± 0.02^{Me}
	70% EtOH	4.78± 0.02^{IJc}	5.65 ± 0.16^{Jd}	9.75 ± 0.02^{Fc}	0.35 ± 0.01^{FGb}	6.50 ± 0.13^{Ca}	5.34 ± 0.09^{Ke}	4.20 ± 0.03^{Me}
	EA	4.57 ± 0.03^{Ja}	-	-	0.81 ± 0.01^{Db}	-	-	-

All values are expressed as the mean ± SD and performed in triplicates. Different letters ($^{a, b, c, d, e}$) within the same column are significantly different (p < 0.05) samples within the solvent whereas letteres ($^{A, B, C, D, E}$) within the same column are significantly different (p < 0.05) samples within the species. Six species of seaweed are reported based on dry weight. TE (Trolox equivalents), EDTA (ethylenediaminetetraacetic acid. FRAP (ferric reducing antioxidant power), DPPH (2,2′-diphenyl-1-picrylhydrazyl), TAC (total antioxidant capacity), ABTS (2,2′-azino-bis-3-ethylbenzothiazoline-6-sulfonic acid), RPA: reducing power assay; ·OH-RSA: hydroxyl radical scavenging activity; FICA: ferrous ion chelating activity. The abbreviation of solvents expressed are ACE (Acetone), MeOH (Methanol), EtOH (Ethanol), EA (Ethyl Acetate).

Table S4: Estimation of freeze-dried bound antioxidant potential of seaweed species extracted by the conventional and non-conventional method.

Samples	Solvents	DPPH (mg TE/g)	FRAP (mg TE/g)	ABTS (mg TE/g)	FICA (mg EDTA/g)	·OH-RSA (mg TE/g)	TAC (mg TE/g)	RPA (mg TE/g)
**Ultrasonication**								
Cystophora sp.	ACE	2.63 ± 0.14^{Bb}	7.15 ± 0.43^{Ca}	10.17 ± 0.04^{Aa}	0.96 ± 0.01 ^{ABCDbc}	3.03 ± 0.27^{Md}	2.87 ± 0.24^{EFa}	8.62 ± 0.17^{Aa}
	MeOH	2.58 ± 0.24^{Bb}	6.25 ± 0.1^{Db}	10.11 ± 0.03^{ABa}	0.94 ± 0.01^{BCDa}	53.73 ± 1.52^{Ba}	10.53 ± 0.28^{Da}	5.60 ± 0.19^{Cb}
	EtOH	2.33 ± 0.19^{BCb}	8.19 ± 0.24^{Ba}	10.03 ± 0.07^{ABa}	0.83 ± 0.01^{EFb}	-	1.36 ± 0.02^{Fc}	5.63 ± 0.57^{Ca}
	EA	1.42 ± 0.5^{Eb}	0.39 ± 0.05^{MNc}	6.58 ± 0.35^{Ib}	0.49 ± 0.03^{Hb}	9.06 ± 0.8^{Jb}	20.24 ± 0.75^{Cc}	1.95 ± 0.6^{GHIb}
Phyllospora comosa	ACE	-	1.38 ± 0.09^{HIJKc}	8.34 ± 0.45^{DEFc}	1.06 ± 0.06^{Aa}	11.96 ± 0.2^{Fc}	0.34 ± 0.03^{Fd}	0.32 ± 0.01^{JKe}
	MeOH	-	2.99 ± 0.04^{Fd}	8.84 ± 0.62^{CDc}	0.92 ± 0.07^{CDEa}	12.35 ± 0.14^{Eb}	0.68 ± 0.01^{Fd}	2.24 ± 0.12^{FGc}
	EtOH	-	1.06 ± 0.05^{JKLd}	8.03 ± 0.12^{EFc}	0.65 ± 0.06^{Gc}	10.45 ± 0.44^{Ha}	0.49 ± 0.05^{Fe}	-
	EA	2.06 ± 0.1^{CDa}	0.53 ± 0.05^{LMNb}	8.57 ± 0.10^{DEa}	1.02 ± 0.05^{ABa}	33.23 ± 2.25^{Ca}	-	2.90 ± 0.25^{Ea}
Sargassum sp.	ACE	3.38 ± 0.16^{Aa}	7.44 ± 0.46^{Ca}	10.13 ± 0.01^{DEa}	0.93 ± 0.01^{BCDc}	3.02 ± 0.09^{Ne}	2.21 ± 4.26^{Fb}	6.02 ± 0.29^{Cb}
	MeOH	3.19 ± 0.22^{Aa}	8.96 ± 0.27^{Aa}	10.10 ± 0.01^{ABa}	0.90 ± 0.01^{DEa}	3.66 ± 0.11^{Kd}	1.94 ± 0.09^{Fb}	6.72 ± 0.17^{Ba}
	EtOH	3.15 ± 0.08^{Aa}	6.50 ± 0.28^{Db}	10.08 ± 0.06^{ABa}	0.80 ± 0.01^{Fb}	1.13 ± 0.05^{Pd}	2.00 ± 0.02^{Fb}	4.43 ± 0.26^{Db}
	EA	0.62 ± 0.04^{Fc}	0.03 ± 0.01^{Ne}	4.59 ± 0.13^{Jc}	0.51 ± 0.02^{Hb}	-	40.30 ± 4.26^{Aa}	-
Ecklonia radiata	ACE	0.76 ± 0.07^{Fd}	2.24 ± 0.17^{Gb}	9.90 ± 0.12^{ABa}	1 ± 0.01^{ABCab}	93.33 ± 5.20^{Ra}	0.37 ± 0.01^{Fe}	1.57 ± 0.08^{HId}
	MeOH	-	4.10 ± 0.07^{Ec}	9.77 ± 0.18^{ABa}	0.66 ± 0.02^{Gb}	11.94 ± 0.71^{Gc}	3.16 ± 0.08^{Fd}	2.53 ± 0.12^{EFc}
	EtOH	0.45 ± 0.04^{Fc}	1.73 ± 0.03^{GHIc}	9.46 ± 0.25^{BCb}	0.96 ± 0.02^{ABCDa}	3.23 ± 0.05^{Lc}	0.91 ± 0.09^{Fd}	0.60 ± 0.01^{Jd}
	EA	-	0.84 ± 0.03^{KLMa}	6.75 ± 0.34^{HIb}	0.31 ± 0.02^{Ic}	-	33.79 ± 2.48^{Bb}	1.88 ± 0.02^{GHIb}
Durvillaea sp.	ACE	1.95 ± 0.11^{Dc}	1.76 ± 0.09^{GHbc}	9.55 ± 0.07^{ABb}	0.64 ± 0.01^{Gd}	13.12 ± 0.73^{Db}	1.20 ± 0.08^{Fc}	3.89 ± 0.12^{Dc}
	MeOH	0.55 ± 0.01^{Dc}	1.52 ± 0.03^{HIJe}	7.73 ± 0.28^{FGc}	0.96 ± 0.01^{BCDa}	2.05 ± 0.05^{Oe}	1.56 ± 0.07^{Fc}	1.75 ± 0.15^{GHId}
	EtOH	0.66 ± 0.01^{Fc}	1.17 ± 0.04^{IJKd}	7.29 ± 0.24^{GHd}	0.79 ± 0.05^{Fb}	10.16 ± 0.14^{Ib}	2.41 ± 0.14^{Fa}	1.4 ± 0.01^{Ic}
	EA	2.01 ± 0.03^{Fc}	0.19 ± 0.02Nd	6.40 ± 0.11^{Ib}	0.12 ± 0.01^{Jd}	-	5.86 ± 0.24^{Ed}	2 ± 0.07^{FGHb}
**Conventional**								
Cystophora sp.	ACE	1.08 ± 0.31^{BCa}	2.12 ± 0.16^{Cb}	3.41 ± 0.01^{Aa}	0.24 ± 0.06^{BCa}	0.7 ± 0.14^{Ed}	0.18 ± 0.05^{Dc}	1.12± 0.09^{B-Db}
	MeOH	1.21 ± 0.29^{ABa}	2.91 ± 0.24^{Aa}	3.33 ± 0.17^{ABa}	0.07 ± 0.01^{HId}	2.2 ± 0.47^{Ed}	5.54 ± 1.14^{Cb}	3.91 ± 0.71^{Aa}
	EtOH	1.15 ± 0.32^{ABa}	2.13 ± 0.07^{Ca}	3.4 ± 0.03^{Aa}	0.18 ± 0.03^{Eb}	3.72 ± 0.29^{Db}	0.35 ± 0.08^{Db}	1.27 ± 0.19^{Bb}
	EA	1.24 ± 0.03^{Ab}	0.25 ± 0.03^{GHb}	3.41 ± 0.07^{Aa}	0.09 ± 0.01^{HIc}	-	17.59 ± 2.03^{Aa}	2.75 ± 0.64^{Aa}
Sargassum sp.	ACE	0.84 ± 0.19^{DEb}	2.34 ± 0.31^{Ba}	3.37 ± 0.08^{ABab}	0.19 ± 0.03^{DEb}	23.37 ± 2.22^{Aa}	1.01 ±0.03^{Da}	2.47 ± 0.37^{Aa}
	MeOH	1.11 ± 0.28^{ABCa}	2.15 ± 0.09^{Cb}	3.37 ± 0.01^{Aa}	0.27 ± 0.01^{Ba}	3.6 ± 0.58^{Dc}	0.27 ± 0.07^{Db}	1.09 ± 0.18^{B-Db}
	EtOH	0.97 ± 0.25^{CDb}	2.12 ± 0.54^{Ca}	3.38 ± 0.07^{Aa}	0.31 ± 0.09^{Aa}	0.39 ± 0.05^{Gd}	0.77 ± 0.17^{Da}	1.24 ± 0.27^{BCa}
	EA	0.56 ± 0.10^{FGc}	0..4 ± 0.05^{FGa}	2.88 ± 0.16^{CDb}	0.14 ± 0.04^{Fa}	-	16.64 ± 4.63^{Da}	0.44 ± 0.12^{GHb}
Ecklonia radiata	ACE	11.67 ± 0.09^{GHc}	4.04 ± 0.23^{DEc}	5.79 ± 0.86^{BCabc}	0.27 ± 0.01^{Fc}	-	6 ± 0.63^{Dc}	2.47 ± 0.03^{BCb}
	MeOH	1.58 ± 0.01^{FGb}	2.01 ± 0.12^{Ec}	3.19 ± 0.03^{ABa}	0.19 ± 0.01^{Bc}	6.75 ± 0.08^{Cb}	4.6 ± 0.31^{Db}	3.13 ± 0.04^{BCDEb}

	EtOH	1.64 ± 0.01^{HIc}	2.89 ± 0.29^{Db}	3.31 ± 0.04^{ABa}	0.33 ± 0.01^{FGc}	1.11 ± 0.13^{Ca}	7.26 ± 0.09^{Dc}	3.54 ± 0.02^{EFb}
	EA	-	0.02 ± 0.01^{HIc}	-	0.31 ± 0.07^{Fa}	-	-	0.14 ± 0.02^{Ic}
Phyllospora comosa	ACE	0.06 ± 0.01^{KLMe}	0.57 ± 0.09^{Fd}	2.87 ± 0.57^{CDc}	0.18 ± 0.02^{Eb}	5.84 ± 0.54^{Bb}	-	0.86 ± 0.09^{Deb}
	MeOH	0.24 ± 0.01^{IJc}	0.54 ± 0.05^{Fd}	2.99 ± 0.30^{CDb}	0.22 ± 0.02^{CDb}	6.37 ± 0.34^{Ba}	-	0.54 ± 0.07^{FG}
	EtOH	0.2 ± 0.02^{IJKc}	0.51 ± 0.04^{Fc}	2.73 ± 0.45^{DEb}	0.19 ± 0.01^{DEb}	1.19 ± 0.09^{Fc}	-	1.28 ± 0.35^{Ba}
	EA	0.69 ± 0.03^{EFb}	0.13 ± 0.02^{Cc}	2.88 ± 0.11^{CDb}	0.1 ± 0.03^{GHbc}	-	-	0.01 ± 0.01^{Ic}
Durvillaea sp.	ACE	0.25 ± 0.07^{IJd}	0.26 ± 0.02^{GHe}	3 ± 0.22^{Cbc}	0.07 ± 0.01^{Id}	4.56 ± 0.76^{Cc}	-	0.19 ± 0.06^{Ic}
	MeOH	0.04 ± 0.01^{LMd}	1.29 ± 0.03^{Ec}	2.85 ± 0.21^{CDb}	0.26 ± 0.06^{Ba}	1.2 ± 0.09^{Fe}	-	0.95 ± 0.13^{CDEb}
	EtOH	-	0.18 ± 0.05^{HId}	2.54 ± 0.13^{Eb}	0.31 ± 0.01^{Aa}	-	-	0.31 ± 0.01^{GHc}
	EA	0.25 ± 0.07^{LMe}	0.03 ± 0.01^{Id}	1.87 ± 0.22^{Fd}	0.12 ± 0.03^{FGHab}	-	8.01 ± 1.55^{Bb}	-

All values are expressed as the mean ± SD and performed in triplicates. Different letters ($^{a, b, c, d, e}$) within the same column are significantly different ($p < 0.05$) samples within the solvent whereas letters ($^{A, B, C, D, E}$) within the same column are significantly different ($p < 0.05$) samples within the species. Six species of seaweed are reported based on dry weight. TE (Trolox equivalents), EDTA (ethylenediaminetetraacetic acid. FRAP (ferric reducing antioxidant power), DPPH (2,2′-diphenyl-1-picrylhydrazyl), TAC (total antioxidant capacity), ABTS (2,2′-azino-bis-3-ethylbenzothiazoline-6-sulfonic acid), RPA: reducing power assay; ·OH-RSA: hydroxyl radical scavenging activity; FICA: ferrous ion chelating activity. The abbreviation of solvents expressed are ACE (Acetone), MeOH (Methanol), EtOH (Ethanol), EA (Ethyl Acetate).

Table S5. Characterization of free phenolic compounds in different seaweed samples with different extraction methods by LC-ESI-QTOF-MS/MS

No.	Proposed compounds	Molecular Formula	RT (min)	Ionization (ESI^+/ESI^-)	Molecular Weight	Theoretical (*m/z*)	Observed (*m/z*)	Error (ppm)	MS^2 Product ions	Seaweed
					**Phenolic acid**					
					**Hydroxybenzoic acids**					
1	Protocatechuic acid 4-*O*-glucoside	$C_{13}H_{16}O_9$	22.261	$[M-H]^-$	316.0791	315.0718	315.0712	-1.9	153	*Eeu
2	Gallic acid 4-*O*-glucoside	$C_{13}H_{16}O_{10}$	30.462	**$[M-H]^-$	332.0768	331.0695	331.0691	-1.2	169, 125	*Amu, Dmu, Aeu, Aau
3	Gallic acid	$C_7H_6O_5$	31.949	$[M-H]^-$	170.0209	169.0136	169.0140	2.4	125	*Aec, Aac, Bac, Bec, Bethc, Bmc, Cec, Cac, Cethc, Dec, Dethc, Dmc, Eeu, Eau
4	3-*O*-Methylgallic acid	$C_8H_8O_5$	33.658	$[M-H]^-$	184.0355	183.0282	183.0280	-1.1	170, 142	*Dec, Dethc, Dmc
					**Hydroxycinnamic acids**					
5	3-Feruloylquinic acid	$C_{17}H_{20}O_9$	4.687	$[M-H]^-$	368.1081	367.1008	367.1012	1.1	298, 288, 192, 191	Cmc
6	Feruloyl tartaric acid	$C_{14}H_{14}O_9$	4.958	$[M-H]^-$	326.0641	325.0568	325.0562	-1.9	193, 149	*Dac, Dethc, Eac, Eau, Cau
7	Caffeic acid	$C_9H_8O_4$	5.116	$[M-H]^-$	180.0431	179.0358	179.0359	0.6	143, 133	Emc
8	Caffeoyl tartaric acid	$C_{13}H_{12}O_9$	5.426	$[M-H]^-$	312.0504	311.0431	311.0438	2.3	161	*Emu, Dmu, Amu
9	Ferulic acid	$C_{10}H_{10}O_4$	5.539	$[M-H]^-$	194.0585	193.0512	193.0516	2.1	178, 149, 134	Eau
10	Isoferulic acid 3-sulfate	$C_{10}H_{10}O_7S$	13.328	$[M-H]^-$	274.0167	273.0094	273.0097	1.1	193, 178	Amu
11	*p*-Coumaroyl malic acid	$C_{13}H_{12}O_7$	17.011	**$[M-H]^-$	280.0579	279.0506	279.0519	4.7	163, 119	*Emu, Aeu
12	Ferulic acid 4-*O*-glucuronide	$C_{16}H_{18}O_{10}$	17.199	**$[M-H]^-$	370.0895	369.0822	369.0836	3.8	193	*Eau, Bmu
13	Rosmarinic acid	$C_{18}H_{16}O_8$	17.543	$[M-H]^-$	360.0822	359.0749	359.0766	4.8	179	Emc, Emu
14	Chicoric acid	$C_{22}H_{18}O_{12}$	17.787	$[M-H]^-$	474.0826	473.0753	473.0739	-3.0	293, 311	Eac
15	Chlorogenic acid	$C_{16}H_{18}O_9$	19.896	$[M-H]^-$	354.0923	353.0850	353.0849	-0.3	253, 190, 144	*Eac, Eau, Aeu
16	3-*p*-Coumaroylquinic acid	$C_{16}H_{18}O_8$	20.108	$[M-H]^-$	338.1023	337.0950	337.0943	-2.1	265, 173, 162	Eau
17	3-Sinapoylquinic acid	$C_{18}H_{22}O_{10}$	23.306	$[M-H]^-$	398.1177	397.1104	397.1109	1.3	233, 179	Eeu
18	Caffeic acid 3-*O*-glucuronide	$C_{15}H_{16}O_{10}$	24.346	$[M-H]^-$	356.0756	355.0683	355.0674	-2.5	179	*Bmc, Bac, Dmc, Emu, Amu
19	1,5-Dicaffeoylquinic acid	$C_{25}H_{24}O_{12}$	24.736	$[M-H]^-$	516.1255	515.1182	515.1159	-4.5	353, 335, 191, 179	*Eec, Eau
20	1,2,2'-Triferuloylgentiobiose	$C_{42}H_{46}O_{20}$	31.165	$[M-H]^-$	870.2584	869.2511	869.2481	-3.5	693, 517	*Aac, Deu, Bau
21	*m*-Coumaric acid	$C_9H_8O_3$	32.821	$[M-H]^-$	164.0477	163.0404	163.0408	2.5	119	Eethc
22	Sinapic acid	$C_{11}H_{12}O_5$	32.135	$[M+H]^+$	224.0672	225.0745	225.0746	0.4	205, 163	Eac

23	1-Sinapoyl-2-feruloylgentiobiose	$C_{33}H_{40}O_{18}$	33.699	$[M+H]^+$	724.2146	725.2219	725.2246	3.7	529, 499	Aeu
					**Hydroxyphenylpropanoic acids**					
24	Dihydrocaffeic acid 3-*O*-glucuronide	$C_{15}H_{18}O_{10}$	30.855	$[M-H]^-$	358.0868	357.0795	357.0798	0.8	181	*Bac, Bec, Cmc
					**Flavonoids**					
					**Flavanols**					
25	Theaflavin	$C_{29}H_{24}O_{12}$	16.799	$[M-H]^-$	564.1257	563.1184	563.1190	1.1	545	*Dac, Emc, Eeu
26	(+)-Catechin	$C_{15}H_{14}O_6$	20.437	**$[M-H]^-$	290.0786	289.0713	289.0716	1.0	245, 205, 179	Eeu
27	(+)-Catechin 3-*O*-gallate	$C_{22}H_{18}O_{10}$	24.152	$[M-H]^-$	442.0876	441.0803	441.0816	3.0	289, 169, 125	*Cac, Eeu
28	Theaflavin 3,3'-*O*-digallate	$C_{43}H_{32}O_{20}$	24.533	$[M-H]^-$	868.1448	867.1375	867.1373	-0.2	715, 563, 545	*Bac, Bmu, Beu, Bau
29	4''-*O*-Methylepigallocatechin 3-*O*-gallate	$C_{23}H_{20}O_{11}$	28.257	[M-H]-	472.1005	471.0932	471.0923	-1.9	169, 319	*Cac, Bec
30	(-)-Epigallocatechin	$C_{15}H_{14}O_7$	30.714	**[M-H]-	306.0737	305.0664	305.0670	2.0	261, 219	*Aau, Dau, Deu
31	4'-*O*-Methyl-(-)-epigallocatechin 7-*O*-glucuronide	$C_{22}H_{24}O_{13}$	34.052	**[M-H]-	496.1186	495.1113	495.1117	0.8	451, 313	*Bmc, Bethc, Dmc, Cac, Dmc, Emc
					**Flavones**					
32	Isorhamnetin	$C_{16}H_{12}O_7$	19.215	$[M-H]^-$	316.0575	315.0502	315.0510	2.5	300, 271	Dmu
33	Apigenin 7-*O*-glucuronide	$C_{21}H_{18}O_{11}$	20.616	**$[M+H]^+$	446.0826	445.0753	445.0755	0.5	271, 253	*Cac, Dmc, Emc, Aac, Aau, Amu
34	Apigenin 7-*O*-(6''-malonyl-apiosyl-glucoside)	$C_{29}H_{30}O_{17}$	20.668	$[M-H]^-$	650.1509	649.1436	649.1445	1.4	605	*Dmc, Aac
35	Apigenin 6-C-glucoside	$C_{21}H_{20}O_{10}$	21.777	$[M-H]^-$	432.1045	431.0972	431.0980	1.9	413, 341, 311	*Dethc, Eec
36	Apigenin 7-*O*-apiosylglucoside	$C_{26}H_{28}O_{14}$	22.663	$[M-H]^-$	564.1524	563.1451	563.1459	1.4	296	Eec
37	3-Methoxysinensetin	$C_{21}H_{22}O_8$	30.813	$[M-H]^-$	402.1301	401.1228	401.1234	1.5	388, 373, 355, 327	*Dec, Eau
38	Apigenin 6,8-di-C-glucoside	$C_{27}H_{30}O_{15}$	32.223	$[M-H]^-$	594.1592	593.1519	593.1509	-1.7	503, 473	*Bac, Aec, Bec, Bethc
					**Flavanones**					
39	Chrysoeriol 7-*O*-glucoside	$C_{22}H_{22}O_{12}$	16.771	$[M-H]^-$	478.1134	477.1061	477.1055	-1.7	445, 427, 409, 381	*Emc, Dac, Dmc, *Eau, Emu
40	Naringin 4'-*O*-glucoside	$C_{33}H_{42}O_{19}$	30.498	$[M-H]^-$	742.2314	741.2241	741.2258	2.3	433, 271	Bec
41	Xanthohumol	$C_{21}H_{22}O_5$	31.272	$[M-H]^-$	354.1486	353.1413	353.1425	3.4	338, 309	Aethc
					**Flavonols**					
42	Kaempferol 3-*O*-glucosyl-rhamnosyl-galactoside	$C_{33}H_{40}O_{20}$	4.719	$[M-H]^-$	756.2135	755.2062	755.2075	1.7	285	*Cmc, Ceu

43	Kaempferol 3,7-*O*-diglucoside	$C_{27}H_{30}O_{16}$	16.377	[M-H]-	610.1542	609.1469	609.1474	0.8	447, 285	Amc
44	Isorhamnetin 3-*O*-glucuronide	$C_{22}H_{20}O_{13}$	16.474	[M-H]-	492.0928	491.0855	491.0872	3.5	315, 300, 272, 255	*Dac, Dmc
45	Myricetin 3-*O*-galactoside	$C_{21}H_{20}O_{13}$	16.921	**[M-H]-	480.0911	479.0838	479.0848	2.1	317	*Emc, Bac, Bethc, Dmc, Eeu, Deu, Beu, Bau
46	Myricetin 3-*O*-rhamnoside	$C_{21}H_{20}O_{12}$	17.258	[M-H]-	464.0946	463.0873	463.0867	-1.3	317	*Dmc, Bac, Bec, Eec, Emc
47	Quercetin 3-*O*-arabinoside	$C_{20}H_{18}O_{11}$	18.213	[M-H]-	434.0837	433.0764	433.0772	1.9	301	*Dac, Dmc, Eeu, Dmu
48	Myricetin 3-*O*-arabinoside	$C_{20}H_{18}O_{12}$	18.956	[M-H]-	450.0756	449.0683	449.0699	3.6	317	*Dmc, Emu, Cau
49	6-Hydroxyluteolin 7-rhamnoside	$C_{21}H_{20}O_{11}$	19.191	[M-H]-	448.1003	447.0930	447.0933	0.7	301	*Emc, Cac, Dac, Dethc, Eec, Emc, Eeu, Eau
50	Quercetin 3-*O*-(6"-malonyl-glucoside)	$C_{24}H_{22}O_{15}$	23.143	[M-H]-	550.0977	549.0904	549.0886	-3.3	303	Bac
51	Quercetin 3-*O*-glucosyl-xyloside	$C_{26}H_{28}O_{16}$	25.144	[M-H]-	596.1402	595.1329	595.1313	-2.7	265, 138, 116	Bac
52	Quercetin 3'-*O*-glucuronide	$C_{21}H_{18}O_{13}$	30.321	[M-H]-	478.0733	477.0660	477.0657	-0.6	301	*Bec, Bac, Bmu, Beu, Bethu, Bau
53	Quercetin 3'-sulfate	$C_{15}H_{10}O_{10}S$	32.872	[M-H]-	382.0021	380.9948	380.9951	0.8	301	*Bmc, Aec, Bethc, Dec, Eac, Ceu, Cau, Beu, Bau, Aau
54	3-Methoxynobiletin	$C_{22}H_{24}O_9$	34.064	[M+H]+	432.1461	433.1534	433.1534	0.1	403, 385, 373, 345	*Aethu
					**Dihydrochalcones**					
55	3-Hydroxyphloretin 2'-*O*-glucoside	$C_{21}H_{24}O_{11}$	4.663	[M-H]-	452.1348	451.1275	451.1277	0.4	289, 273	*Aac, Aec, Bec, Cac, Eec, Bau
					**Dihydroflavonols**					
56	Dihydroquercetin	$C_{15}H_{12}O_7$	15.971	[M-H]-	304.0591	303.0518	303.0518	0.1	285, 275, 151	*Emu
57	Dihydroquercetin 3-*O*-rhamnoside	$C_{21}H_{22}O_{11}$	19.734	[M-H]-	450.1147	449.1074	449.1077	0.7	303	*Eec, Dethc, Dmc, Emc
58	Dihydromyricetin 3-*O*-rhamnoside	$C_{21}H_{22}O_{12}$	23.443	[M-H]-	466.1111	465.1038	465.1047	1.9	301	*Emc, Dac, Dethc, Eec, Eau, Deu
					**Anthocyanins**					
59	Genistein 4',7-*O*-diglucuronide	$C_{27}H_{26}O_{17}$	23.862	**[M-H]-	622.1174	621.1101	621.1122	3.4	269	*Aac, Bec, Dethc, Emu, Eeu, Dau, Cau, *Bau, Aau
60	Delphinidin 3-*O*-glucoside	$C_{21}H_{21}O_{12}$	26.489	[M-H]-	465.1016	464.0943	464.0940	-0.7	303	Bac
61	Pelargonidin	$C_{15}H_{11}O_5$	32.266	[M-H]-	271.0618	270.0545	270.0557	4.4	243, 197, 169, 141	Eac

62	Peonidin 3-*O*-diglucoside-5-*O*-glucoside	$C_{34}H_{43}O_{21}$	33.383	[M-H]$^-$	787.2331	786.2258	786.2246	-1.5	625, 478, 317	*Cethc, Dec
	**Isoflavonoids**									
63	6''-*O*-Acetyldaidzin	$C_{23}H_{22}O_{10}$	4.703	[M-H]$^-$	458.1218	457.1145	457.1139	-1.3	221	Bmc
64	5,6,7,3',4'-Pentahydroxyisoflavone	$C_{15}H_{10}O_7$	5.266	[M-H]$^-$	302.0439	301.0366	301.0376	3.3	285, 257	Amc
65	6''-*O*-Acetylglycitin	$C_{24}H_{24}O_{11}$	5.896	[M+H]$^+$	488.1333	489.1406	489.1398	-1.6	285, 270	*Amu
66	2'-Hydroxyformononetin	$C_{16}H_{12}O_5$	5.934	**[M-H]$^-$	284.0687	283.0614	283.0619	1.8	270, 229	*Dmc, Cac, Emc, Emu, Eeu, Dmu
67	2',7-Dihydroxy-4',5'-dimethoxyisoflavone	$C_{17}H_{14}O_6$	6.882	[M-H]$^-$	314.0767	313.0694	313.0698	1.3	300, 282	*Eac, Eec
68	6''-*O*-Malonylgenistin	$C_{24}H_{22}O_{13}$	14.363	**[M+H]$^+$	518.1069	517.0996	517.0997	0.2	271	Eac
69	2-Dehydro-*O*-desmethylangolensin	$C_{15}H_{12}O_4$	16.548	[M-H]$^-$	256.0746	255.0673	255.0667	-2.4	135, 119	*Emc, Bmc
70	Sativanone	$C_{17}H_{16}O_5$	16.563	**[M-H]$^-$	300.0994	299.0921	299.0923	0.7	284, 269, 225	*Aec, Aac, Amc, Bec, Aec, Amc, Cac, Emc, Deu, Emu, Eau
71	Violanone	$C_{17}H_{16}O_6$	16.651	[M-H]$^-$	316.0933	315.086	315.0868	2.5	300, 285, 135	*Eeu
72	6''-*O*-Malonyldaidzin	$C_{24}H_{22}O_{12}$	16.73	[M-H]$^-$	502.1117	501.1044	501.1036	-1.6	255	*Dac, Cac, Eac, Emc, Bau
73	6''-*O*-Malonylglycitin	$C_{25}H_{24}O_{13}$	18.919	[M-H]$^-$	532.1215	531.1142	531.1145	0.6	285, 270, 253	*Emc, Eec
74	Pseudobaptigenin	$C_{16}H_{10}O_5$	19.16	**[M-H]$^-$	282.0506	281.0433	281.0431	-0.7	263, 237	*Dau, Aau
75	Dalbergin	$C_{16}H_{12}O_4$	21.505	[M-H]$^-$	268.0717	267.0644	267.0656	4.5	252, 224, 180	*Deu, Ceu
76	Formononetin 7-*O*-glucuronide	$C_{22}H_{20}O_{10}$	21.977	[M-H]$^-$	444.1048	443.0975	443.0972	-0.7	267, 252	*Cac, Eac
	**Other polyphenols**									
	**Hydroxycoumarins**									
77	Esculetin	$C_9H_6O_4$	24.158	[M-H]$^-$	178.0280	177.0207	177.0207	0.1	149, 133, 89	*Bau, Bec
78	Scopoletin	$C_{10}H_8O_4$	30.978	[M-H]$^-$	192.0406	191.0333	191.0336	1.6	176	*Dmc, Bec, Bmc, Cac, Eec, Emc, Eeu, Eau, Bmu, Beu, Bau
	**Hydroxybenzaldehydes**									
79	*p*-Anisaldehyde	$C_8H_8O_2$	30.932	[M-H]$^-$	136.0516	135.0443	135.0442	-0.7	122, 109	*Bethc, Eac, Eec, Eethc, Emc
80	3-Hydroxy-3-(3-hydroxyphenyl) propionic acid	$C_9H_{10}O_4$	32.498	[M-H]$^-$	182.0582	181.0509	181.0507	-1.1	163, 135, 119	Eac
81	2-Hydroxy-2-phenylacetic acid	$C_8H_8O_3$	33.492	[M-H]$^-$	152.0465	151.0392	151.0395	2.0	136, 92	*Eethc, Dmc, Eac, Aeu, Aau
	**Hydroxybenzoketones**									
82	2-Hydroxy-4-methoxyacetophenone 5-sulfate	$C_9H_{10}O_7S$	24.081	**[M-H]$^-$	262.0155	261.0082	261.0081	-0.4	181, 97	*Aeu, Aau

				**Alkylmethoxyphenols**						
83	Equol	$C_{15}H_{14}O_3$	16.947	$[M+H]^+$	242.0952	243.1025	243.1027	0.8	255, 211, 197	*Amc, Aac, Aec, Eac, Eethc, Emu, Eau, Dau
				**Phenolic terpenes**						
84	Carnosic acid	$C_{20}H_{28}O_4$	32.446	$[M-H]^-$	332.1984	331.1911	331.1912	0.3	287, 269	*Emc, Emu, Eau, Dmu, Deu, Dau, Ceu, Cethu, Cau, Bmu, Beu, Bethu, Bau, Amu, Aau
				**Tyrosols**						
85	3,4-DHPEA-AC	$C_{10}H_{12}O_4$	24.521	$[M-H]^-$	196.0736	195.0663	195.0662	-0.5	135	*Amu, Cac
				**Other polyphenols**						
86	Arbutin	$C_{12}H_{16}O_7$	19.621	$[M-H]^-$	272.0901	271.0828	271.0827	-0.4	109	*Ceu, Cau
87	Lithospermic acid	$C_{27}H_{22}O_{12}$	22.962	**$[M-H]^-$	538.1142	537.1069	537.1075	1.1		*Dmc, Cec
				**Lignans**						
88	Arctigenin	$C_{21}H_{24}O_6$	4.853	$[M-H]^-$	372.1544	371.1471	371.1467	-1.1	493, 339, 295	*Aethc, Dmu, Dau, Cau, Aeu, Aau
89	Sesamin	$C_{20}H_{18}O_6$	24.500	**$[M-H]^-$	354.1138	353.1065	353.1068	0.9	338, 163	*Eec, Deu, Aeu
90	7-Hydroxymatairesinol	$C_{20}H_{22}O_7$	31.735	$[M-H]^-$	374.1335	373.1262	373.1265	0.8	343, 313, 298, 285	Aac
91	Secoisolariciresinol-sesquilignan	$C_{30}H_{38}O_{10}$	31.907	$[M-H]^-$	558.2434	557.2361	557.2387	4.7	539, 521, 509, 361	*Aec, Amu

*Compound was detected in more than one seaweed samples, data presented in this table are from asterisk sample. **Compounds were detected in both negative $[M-H]^-$ and positive $[M+H]^+$ mode of ionization while only single mode data was presented. Seaweed samples were mentioned in abbreviations.

Table S6. Characterization of bound phenolic compounds in different seaweed samples with different extraction methods by LC-ESI-QTOF-MS/MS

No.	Proposed compounds	Molecular Formula	RT (min)	Ionization (ESI^+/ESI^-)	Molecular Weight	Theoretical (*m/z*)	Observed (*m/z*)	Error (ppm)	MS^2 Product ions	Seaweed
					**Phenolic acid**					
					**Hydroxybenzoic acids**					
1	Protocatechuic acid 4-*O*-glucoside	$C_{13}H_{16}O_9$	3.062	$[M-H]^-$	316.0818	315.0745	315.0751	1.9	153	*Cethbc, Cebc, Debc, Dmbc, Dmbu, Embu
2	Gallic acid 4-*O*-glucoside	$C_{13}H_{16}O_{10}$	7.404	$[M-H]^-$	332.0737	331.0664	331.0670	1.8	169, 125	*Cabu, Cebu, Dmbu
3	4-Hydroxybenzoic acid 4-*O*-glucoside	$C_{13}H_{16}O_8$	7.687	$[M-H]^-$	300.0839	299.0766	299.0773	2.3	255, 137	Dabc
4	4-Hydroxybenzaldehyde	$C_7H_6O_2$	25.146	$[M-H]^-$	122.0366	121.0293	121.0294	0.8	77	*Bethbc, Cmbc, Dabc, Dabu, Dmbc
5	2,3-Dihydroxybenzoic acid	$C_7H_6O_4$	25.802	$[M-H]^-$	154.0262	153.0189	153.0190	0.7	109	*Bethbc, Eethbu
6	Gallic acid	$C_7H_6O_5$	31.309	$[M-H]^-$	170.0225	169.0152	169.0154	1.2	125	*Aabu, Aabc, Aebu, Ambu, Babu, Bebu, Bethbu, Bmbu, Debc, Dethbu, Eabu, Eethbu
7	2-Hydroxybenzoic acid	$C_7H_6O_3$	32.019	$[M-H]^-$	138.0310	137.0237	137.0238	0.7	93	*Bethbc, Dabu, Dmbu
					**Hydroxycinnamic acids**					
8	1-Sinapoyl-2,2'-diferuloylgentiobiose	$C_{43}H_{48}O_{21}$	3.119	[M-H]-	900.2677	899.2604	899.2574	-3.3	613, 201	*Aebc, Debc
9	*m*-Coumaric acid	$C_9H_8O_3$	5.228	$[M-H]^-$	164.0487	163.0414	163.0412	-1.2	119	*Dabu, Cmbu, Debu, Dmbu, Eabu
10	Isoferulic acid 3-sulfate	$C_{10}H_{10}O_7S$	5.608	$[M-H]^-$	274.0129	273.0056	273.0054	-0.7	193, 178	*Aabu, Aethbu, Ambu, Bmbu, Cabu, Dabu
11	Caffeic acid 3-*O*-glucuronide	$C_{15}H_{16}O_{10}$	6.388	**$[M-H]^-$	356.0743	355.0670	355.0673	0.8	179	*Ambc, Dethbc, Cebu, Ambu, Embu, Cethbu
12	Cinnamic acid	$C_9H_8O_2$	7.246	**$[M-H]^-$	148.0537	147.0464	147.0465	0.7	103	*Ambc, Cmbc, Cebu, Aethbu, Bmbu, Cabu, Dmbu, Dabu, Eabu

13	Ferulic acid 4-*O*-glucoside	$C_{16}H_{20}O_9$	13.750	[M-H]-	356.1106	355.1033	355.1025	-2.3	193, 178, 149, 134	*Aabu, Babu
14	Chlorogenic acid	$C_{16}H_{18}O_9$	13.985	[M-H]-	354.0929	353.0856	353.0855	-0.3	253, 190, 144	*Cabc, Dabu, Babu, Cabu, Cebu, Eabu, Eabc
15	1-Sinapoyl-2-feruloylgentiobiose	$C_{33}H_{40}O_{18}$	14.898	[M-H]-	724.2205	723.2132	723.2136	0.6	529, 499	*Bmbu, Cabu, Cethbu, Cmbu, Dmbu, Eebu
16	Ferulic acid 4-*O*-glucuronide	$C_{16}H_{18}O_{10}$	14.994	[M-H]-	370.0886	369.0813	369.0811	-0.5	193	**Cebc, Dethbc, Dabu, Debu, Embu
17	*p*-Coumaroyl tartaric acid	$C_{13}H_{12}O_8$	16.214	[M-H]-	296.0555	295.0482	295.0486	1.4	115	*Dmbc, Debu, Dmbu, Embu
18	*p*-Coumaroyl malic acid	$C_{13}H_{12}O_7$	16.596	**[M-H]-	280.0596	279.0523	279.0512	-3.9	163, 119	*Aebc, Ambc, Debc, Dmbu, Cabu, Cebu, Cethbu, Dabu, Dmbu
19	1,5-Dicaffeoylquinic acid	$C_{25}H_{24}O_{12}$	17.017	[M-H]-	516.1233	515.1160	515.1172	2.3	353, 335, 191, 179	*Dabu, Eabu
20	Hydroxycaffeic acid	$C_9H_8O_5$	17.250	[M-H]-	196.0376	195.0303	195.0304	0.5	151	*Aabc, Bethbc, Dabc, Dethbc, Cebu
21	*p*-Coumaric acid 4-*O*-glucoside	$C_{15}H_{18}O_8$	17.367	[M-H]-	326.0970	325.0897	325.0891	-1.8	163	*Bebc, Eabu, Dabu
22	Caffeoyl glucose	$C_{15}H_{18}O_9$	18.549	[M-H]-	342.0951	341.0878	341.0890	3.5	179, 161	*Dmbu, Dabu, Eabu, Eebc
23	Rosmarinic acid	$C_{18}H_{16}O_8$	18.986	[M-H]-	360.0823	359.0750	359.0753	0.8	179	*Dmbc, Eabc, Dmbu
24	3-*p*-Coumaroylquinic acid	$C_{16}H_{18}O_8$	19.117	[M-H]-	338.0998	337.0925	337.0926	0.3	265, 173, 162	*Dabc, Dabu, Cabu, Eabu, Eebu
25	Cinnamoyl glucose	$C_{15}H_{18}O_7$	20.687	[M-H]-	310.1035	309.0962	309.0955	-2.3	147, 131, 103	*Dabc, Debu, Eabc
26	5-5'-Dehydrodiferulic acid	$C_{20}H_{18}O_8$	21.657	**[M-H]+	386.0985	385.0912	385.0913	0.3	369	*Eabu, Embu, Cebu
					**Hydroxyphenylacetic acids**					
27	3,4-Dihydroxyphenylacetic acid	$C_8H_8O_4$	15.889	[M-H]-	168.0426	167.0353	167.0354	0.6	149, 123	Aabc
					**Hydroxyphenylpentanoic acids**					
28	Sinapic acid	$C_{11}H_{12}O_5$	22.780	**[M-H]-	224.0674	223.0601	223.0598	-1.3	205, 163	*Cabc, Aabc, Ambc, Babc, Bebc, Bmbc, Cebc, Eabc, Aabu, Cethbu, Dabu, Debu, Dmbu
					**Hydroxyphenylpropanoic acids**					
29	Dihydroferulic acid 4-*O*-glucuronide	$C_{16}H_{20}O_{10}$	3.119	**[M-H]-	372.1090	371.1017	371.1019	0.5	195	*Aabc, Aebc, Cabc, Cebc, Cmbc, Debc, Dmbc, Eabc, Aethbc, Dethbc, Babu,

										Bebu, Bmbu, Dabu, Eabu, Embu, Cethbu
30	Dihydrocaffeic acid 3-*O*-glucuronide	$C_{15}H_{18}O_{10}$	7.535	**$[M-H]^-$	358.0913	357.0840	357.0837	-0.8	181	*Bebu, Bmbu. Debu, Eabu, Eebu, Embu, Aabu,
31	Dihydroferulic acid 4-sulfate	$C_{10}H_{12}O_7S$	16.763	$[M-H]^-$	276.0290	275.0217	275.0212	-1.8	195, 151, 177	Eebu
					**Flavonoids**					
					**Flavanols**					
32	Theaflavin	$C_{29}H_{24}O_{12}$	3.082	$[M-H]^-$	564.1254	563.1181	563.1198	3.0	545	*Embc, Bebu, Dabu
33	4''-*O*-Methylepigallocatechin 3-*O*-gallate	$C_{23}H_{20}O_{11}$	7.467	**$[M-H]^-$	472.1028	471.0955	471.0972	3.6	169, 319	Debc
34	(+)-Gallocatechin 3-*O*-gallate	$C_{22}H_{18}O_{11}$	7.625	$[M-H]^-$	458.0830	457.0757	457.0755	-0.4	305, 169	*Babc, Bebc, Cebc, Debc, Cebu, Debu
35	(+)-Catechin	$C_{15}H_{14}O_6$	14.525	$[M-H]^-$	290.0767	289.0694	289.0697	1.0	245, 205, 179	Dabu
36	Prodelphinidin dimer B3	$C_{30}H_{26}O_{14}$	16.094	$[M+H]^+$	610.1318	611.1391	611.1366	-4.1	469, 311, 291	Aebc
37	3'-*O*-Methylcatechin	$C_{16}H_{16}O_6$	17.951	**$[M-H]^-$	304.0959	303.0886	303.0894	2.6	271, 163	Babu
38	Procyanidin dimer B1	$C_{30}H_{26}O_{12}$	18.209	**$[M-H]^-$	578.1444	577.1371	577.1378	1.2	451	*Cabc, Aebc, Dethbc, Babu
39	(+)-Catechin 3-*O*-gallate	$C_{22}H_{18}O_{10}$	20.261	$[M-H]^-$	442.0879	441.0806	441.0811	1.1	289, 169, 125	Embu
40	4'-*O*-Methyl-(-)-epigallocatechin 7-*O*-glucuronide	$C_{22}H_{24}O_{13}$	20.795	$[M-H]^-$	496.1187	495.1114	495.1117	0.6	451, 313	Embc
41	(-)-Epigallocatechin	$C_{15}H_{14}O_7$	21.240	$[M-H]^-$	306.0749	305.0676	305.0674	-0.7	261, 219	**Aabc, Eabc, Debu, Eabu, Embu
					**Flavones**					
42	Genistein 4',7-O-diglucuronide	$C_{27}H_{26}O_{17}$	7.415	$[M-H]^-$	622.1149	621.1076	621.1088	1.9	269	Debc
43	3-Methoxysinensetin	$C_{21}H_{18}O_{11}$	16.159	$[M-H]^-$	446.0883	445.0810	445.0802	-1.8	271, 253	Dabc
44	Apigenin 6-C-glucoside	$C_{21}H_{20}O_{10}$	16.981	$[M-H]^-$	432.1077	431.1004	431.1015	2.6	413, 341, 311	*Dabu, Dmbu
					**Flavanones**					
45	Hesperetin 3',7-*O*-diglucuronide	$C_{28}H_{30}O_{18}$	4.806	$[M-H]^-$	654.1424	653.1351	653.1356	0.8	477, 301, 286, 242	*Embc, Babu, Cmbu, Debu, Eabu
46	Narirutin	$C_{27}H_{32}O_{14}$	5.264	$[M-H]^-$	580.1827	579.1754	579.1756	0.3	271	*Cmbu, Cabu, Cebu

47	Hesperetin 3'-sulfate	$C_{16}H_{14}O_9S$	7.389	[M-H]⁻	382.0354	381.0281	381.0277	-1.0	301, 286, 257, 242	*Babc, Bebc, Cebc, Dmbc, Embc, Aethbu, Ambu, Babu, Cmbu, Dmbu, Eabu
48	Hesperetin 3'-*O*-glucuronide	$C_{22}H_{22}O_{12}$	20.774	[M-H]⁻	478.1106	477.1033	477.1027	-1.3	301, 175, 113, 85	*Dabc, Eabc, Eebc
49	Naringin 4'-*O*-glucoside	$C_{33}H_{42}O_{19}$	32.747	[M-H]⁻	742.2295	741.2222	741.2232	1.3	433, 271	Cethbu
					**Flavonols**					
50	Quercetin 3'-*O*-glucuronide	$C_{21}H_{18}O_{13}$	5.185	[M-H]⁻	478.0758	477.0685	477.0679	-1.3	301	Cabu
51	6-Hydroxyluteolin 7-rhamnoside	$C_{21}H_{20}O_{11}$	5.667	[M-H]⁻	448.0964	447.0891	447.0888	-0.7	301	*Cmbc, Eebc
52	Myricetin 3-*O*-arabinoside	$C_{20}H_{18}O_{12}$	7.182	[M-H]⁻	450.0821	449.0748	449.0750	0.4	317	Cebu
53	Quercetin 3-*O*-glucosyl-xyloside	$C_{26}H_{28}O_{16}$	15.852	[M-H]⁻	596.1347	595.1274	595.1280	1.0	265, 138, 116	Eabu
54	Isorhamnetin 3-*O*-glucuronide	$C_{22}H_{20}O_{13}$	15.938	[M-H]⁻	492.0897	491.0824	491.0821	-0.6	315, 300, 272, 255	Aabu
55	Quercetin 3-*O*-arabinoside	$C_{20}H_{18}O_{11}$	16.516	[M-H]⁻	434.0851	433.0778	433.0784	1.4	301	Dmbu
56	Quercetin 3-*O*-xylosyl-rutinoside	$C_{32}H_{38}O_{20}$	33.752	[M+H]⁺	742.1933	743.2006	743.2003	-0.4	479, 317	Debc
					**Dihydroflavonols**					
57	Dihydroquercetin	$C_{15}H_{12}O_7$	7.469	[M-H]⁻	304.0599	303.0526	303.0511	-4.9	285, 275, 151	Dabu
58	Dihydroquercetin 3-*O*-rhamnoside	$C_{21}H_{22}O_{11}$	20.314	[M-H]⁻	450.1166	449.1093	449.1084	-2.0	303	*Eabc, Dabc, Eabc
					**Anthocyanins**					
59	Cyanidin 3,5-*O*-diglucoside	$C_{27}H_{31}O_{16}$	32.278	[M+H]⁺	611.1632	612.1705	612.1711	1.0	449, 287	*Aethbc, Bebc, Debc
					**Isoflavonoids**					
60	2-Dehydro-*O*-desmethylangolensin	$C_{15}H_{12}O_4$	5.898	[M-H]⁻	256.0751	255.0678	255.0679	0.4	135, 119	*Cmbu, Babu
61	Violanone	$C_{17}H_{16}O_6$	5.937	[M-H]⁻	316.0932	315.0859	315.0850	-2.9	300, 285, 135	**Aebc, Ambc, Cmbu, Embu
62	Sativanone	$C_{17}H_{16}O_5$	16.691	[M+H]⁺	300.0994	301.1067	301.1066	-0.3	284, 269, 225	Ambc
63	Glycitin	$C_{22}H_{22}O_{10}$	30.888	[M-H]⁻	446.1206	445.1133	445.1124	-2.0	285	*Cethbu, Eabu
64	6''-*O*-Malonyldaidzin	$C_{24}H_{22}O_{12}$	33.752	**[M+H]⁺	502.1093	503.1166	503.1178	2.4	255	*Debc, Dabc, Embc

					**Other polyphenols**					
					**Hydroxycoumarins**					
65	Scopoletin	$C_{10}H_8O_4$	31.153	[M-H]$^-$	192.0407	191.0334	191.0335	0.5	176	*Babc, Aebc, Bebc, Bethbc, Bmbc, Dabc, Debc, Dmbc, Bethbu
					**Hydroxybenzaldehydes**					
66	2-Hydroxy-2-phenylacetic acid	$C_8H_8O_3$	14.463	[M-H]$^-$	152.0473	151.0400	151.0401	0.7	136, 92	*Cmbc, Aabc, Cabc, Dabc, Eabc, Cebu
					**Hydroxyphenylpropenes**					
					**Alkylmethoxyphenols**					
67	Equol	$C_{15}H_{14}O_3$	18.110	[M+H]$^+$	242.0943	243.1016	243.1014	-0.8	255, 211, 197	Cabc
					**Phenolic terpenes**					
68	Carnosic acid	$C_{20}H_{28}O_4$	32.549	**[M-H]-	332.2004	331.1931	331.1933	0.6	287, 269	*Dethbc, Eabc, Dethbu, Cethbu
					**Tyrosols**					
69	Hydroxytyrosol 4-*O*-glucoside	$C_{14}H_{20}O_8$	16.175	[M-H]-	316.1149	315.1076	315.1076	0.1	153, 123	Cmbc
70	3,4-DHPEA-EDA	$C_{17}H_{20}O_6$	29.370	[M-H]-	320.127	319.1197	319.1192	-1.6	275, 195	*Bethbc, Aabu, Bmbu
					**Other polyphenols**					
71	Salvianolic acid B	$C_{36}H_{30}O_{16}$	16.693	[M-H]$^-$	718.1517	717.1444	717.1421	-3.2	519, 339, 321, 295	Embc
72	Lithospermic acid	$C_{27}H_{22}O_{12}$	17.324	**[M-H]$^-$	538.1097	537.1024	537.1018	-1.1	493, 339, 295	*Eabc, Cabc, Cethbc
73	Salvianolic acid C	$C_{26}H_{20}O_{10}$	21.929	[M-H]$^-$	492.1052	491.0979	491.0989	2.0	311, 267, 249	*Debc, Dmbc, Embu
					**Lignans**					
74	Schisandrol B	$C_{23}H_{28}O_7$	3.062	**[M-H]$^-$	416.1824	415.1751	415.1749	-0.5	224, 193, 165	Aebu
75	Sesamin	$C_{20}H_{18}O_6$	13.750	[M-H]$^-$	354.1120	353.1047	353.1037	-2.8	338, 163	*Aabu, Babu
76	Todolactol A	$C_{20}H_{24}O_7$	16.242	[M-H]$^-$	376.1546	375.1473	375.1469	-1.1	313, 137	*Debc, Embc, Ambu, Dmbu
77	Arctigenin	$C_{21}H_{24}O_6$	26.476	[M-H]$^-$	372.1565	371.1492	371.1494	0.5	356, 312, 295	*Embu, Eebu
78	7-Hydroxymatairesinol	$C_{20}H_{22}O_7$	28.718	**[M-H]$^-$	374.1379	373.1306	373.1311	1.3	343, 313, 298, 285	*Bethbc, Dabc, Eebc, Aebu, Eebu
79	Secoisolariciresinol-sesquilignan	$C_{30}H_{38}O_{10}$	31.577	[M-H]$^-$	558.2471	557.2398	557.2372	-4.7	539, 521, 509, 361	Debc

80	7-Oxomatairesinol	$C_{20}H_{20}O_7$	32.347	$[M+H]^+$	372.1184	373.1257	373.1256	-0.3	358, 343, 328, 325	*Babc, Bebc, Bethbc, Cmbc
					**Stilbenes**					
81	Resveratrol	$C_{14}H_{12}O_3$	13.985	$[M-H]^-$	228.0806	227.0733	227.0739	2.6	212, 185, 157, 143	Dabu
82	Resveratrol 5-*O*-glucoside	$C_{20}H_{22}O_8$	33.742	$[M-H]^-$	390.1283	389.1210	389.1214	1.0	227	*Embc, Debu

*Compound was detected in more than one seaweed samples, data presented in this table are from asterisk sample. **Compounds were detected in both negative $[M-H]^-$ and positive $[M+H]^+$ mode of ionization while only single mode data was presented.
